# Protein Structuromics Reveals a Loop‐Controlled Half‐Open Active Pocket Conformation Throughout Fe(II)/α‐ketoglutarate‐Dependent Dioxygenase Catalytic Cycle

**DOI:** 10.1002/advs.75853

**Published:** 2026-05-26

**Authors:** Lunjie Wu, Huan Liu, Songyin Zhao, Lei Qin, Jiarui Li, Jun Wang, Zixuan Dai, Jie Gu, Yan Xu, Feiran Li, Yao Nie

**Affiliations:** ^1^ Laboratory of Brewing Microbiology and Applied Enzymology School of Biotechnology and Key Laboratory of Industrial Biotechnology Ministry of Education Jiangnan University Wuxi China; ^2^ Institute of Biopharmaceutical and Health Engineering Tsinghua Shenzhen International Graduate School Tsinghua University Shenzhen China; ^3^ Key Laboratory of Biocatalysis & Chiral Drug Synthesis of Guizhou Province Generic Drug Research Center of Guizhou Province Green Pharmaceuticals Engineering Research Center of Guizhou Province School of Pharmacy Zunyi Medical University Zunyi China; ^4^ Key Laboratory of Industrial Synthetic Biology of Jiangsu Province Jiangnan University Wuxi China

**Keywords:** Fe(II)/α‐ketoglutarate‐dependent dioxygenases, half‐open active pocket, multifaceted loop, protein structuromics

## Abstract

The Fe(II)/α‐ketoglutarate‐dependent dioxygenase (αKGD) superfamily enables various C−H functionalization supported by their active pocket architectures composed of one or more loop elements. However, their conserved architectural features and broader functional roles across the catalytic cycle remain incompletely defined, although such loops have been implicated in substrate recognition or proton transfer in specific catalytic processes. Here, we applied protein structuromics analysis to the PF10014 family within the αKGD superfamily, which contains a single long active‐pocket loop and therefore provides a tractable model for dissecting loop‐associated structure–function relationships. This analysis identified a conserved structural motif termed the half‐open active pocket, which is proposed as a hallmark feature of the αKGD superfamily. Furthermore, enhanced sampling simulations and mutagenesis experiments targeting isoleucine dioxygenase, a representative member of the PF10014 family, revealed the multifaceted role of the loop that constitutes the half‐open active pocket architecture throughout the catalytic cycle, encompassing the modulation of half‐open active pocket conformation, substrate recognition and anchoring, as well as participation in molecular transport. These findings provide a broader structure–function landscape in which the flexible loop within this family‐conserved half‐open active pocket serves as a key catalytic element with multiple functions.

## Graphical abstract

This study reveals a conserved half‐open active pocket in Fe(II)/α‐ketoglutarate‐dependent dioxygenases through protein structuromics, distinguished by structural features comprising highly flexible loop elements. Enhanced sampling simulations‐guided mutagenesis experiments elucidated how this flexible loop regulates pocket open/closed conformations, guides substrate binding, and assists molecular transport. These insights clarify how loop dynamics support catalysis and inform future enzyme engineering.

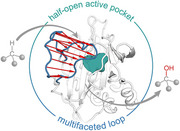

## 1 Introduction

The Fe(II)/α‐ketoglutarate‐dependent dioxygenase (αKGD) superfamily plays pivotal roles in diverse biological processes, including cellular metabolism, oxygen sensing, and nucleic acid repair [1]. The remarkable functional diversity exhibited by the αKGD superfamily arises from their exceptional oxygen‐transfer capability, which enables various C–H functionalization reactions such as hydroxylation, demethylation, epoxidation, and ring rearrangement [1, 2]. Owing to their outstanding catalytic function, αKGDs have been extensively harnessed for the biosynthesis of high‐value compounds ranging from non‐canonical amino acids [3, 4, 5] to natural products [6, 7, 8, 9].

**FIGURE 1:**
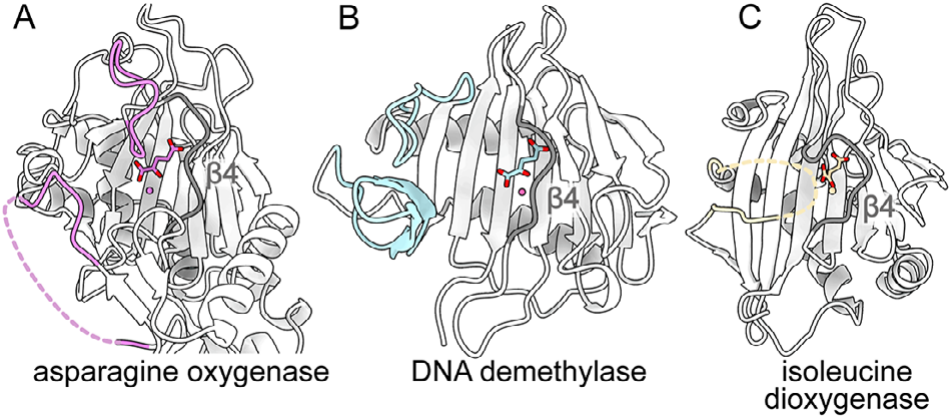
Loop profiles composing active pockets in different αKGDs members. Loop profile of asparagine hydroxylase (A, PDB: 2OG5), DNA demethylase (B, PDB: 4MGT), and isoleucine dioxygenase (C, PDB: 6LNH). Fe^II^ and αKG are shown as stick style, and dashed lines indicate residue missing.

Interestingly, despite the diverse reaction types catalyzed by different αKGD members, their active pocket architectures supporting these reactions share similar structural compositions, generally comprising a β‐sheet (β4) from the family‐conserved DSBH fold and one or more loops [10]. Given the pronounced rigidity of the β‐sheet, the functional diversification among various members is likely associated with the flexible loops. For example, the recently reported crystal structure of TraH, a natural product biosynthesis‐related enzyme in the αKGD superfamily, revealed an N‐terminal loop that acts as a “lid”, undergoes substrate‐dependent dynamic conformational rearrangement, and further mediates proton transfer upon substrate binding [11]. Crystallography of the αKGD asparagine hydroxylase revealed that its active pocket comprises two loops, whose conformations exhibit significant differences between the substrate‐bound and unbound states (Figure 1A) [12]. Similarly, the active pocket architecture of AlkB, an αKGD member possessing alkylation repair activity, comprises two loops that anchor alkylated substrates in optimal catalytic orientations through their conformational changes (Figure 1B) [13, 14]. By contrast, the active pocket of isoleucine dioxygenase (IDO), another αKGD member, contains only a single long loop, yet this loop likewise undergoes conformational rearrangements to facilitate substrate anchoring (Figure 1C) [15]. To date, accumulating evidence underscores the importance of loop elements in the evolution of enzyme function [16, 17]. Although these loops have been implicated in individual enzymes to participate in discrete functions, such as substrate recognition or proton transfer, their conserved architectural features and broader functional roles across the catalytic cycle remain incompletely explored. Consequently, elucidation of the structural and functional landscape of the loops constituting the active pocket is expected to provide deeper insight into the functional evolution of the αKGD superfamily.

Although the function of loops constituting the active pocket has been noted in studies of different αKGD members, whether these loops are broadly conserved across the αKGD superfamily and whether they perform additional roles throughout the catalytic cycle remain to be elucidated in detail. The rise of structural biology has greatly facilitated the acquisition of structure‐based functional insights, yet inadequate availability of crystallographic data for αKGDs continues to constrain exploring of their structural and functional landscape. Offering a potential solution to this problem, the recent proliferation of AI‐based protein structure prediction tools, such as AlphaFold [18], ESMfold [19], and RoseTTAFold [20], has enabled rapid and facile access to massive sets of protein structures. Specifically, tools for the retrieval of structural homologs (such as Foldseek [21]) allow the exploration of an extensive protein structure universe, whereas programs for structural comparison (such as TM‐align [22]) can provide insight into the structural relationships within the retrieved protein sets. This analytical framework for exploring protein structural landscapes at the omics level has been termed “protein structuromics” [23, 24]. However, although exploring the structural features of αKGDs is crucial to understanding their catalytic functions, the protein structuromics approach has not yet been applied to this enzyme superfamily.

In this study, we aimed to investigate the functional roles of loop elements forming the active pocket within the αKGD superfamily throughout the catalytic cycle. The PF10014 family (represented by IDO) within αKGD superfamily possesses active pockets containing only a single long loop [25], providing a readily investigable system for studying the structure‐function relationship of the active pocket in αKGD superfamily. Therefore, this enzyme family was subjected to the protein structuromics analysis, through which a structural motif termed the half‐open active pocket was identified. This structural motif comprises a rigid β‐sheet coupled with flexible loops exhibiting extensive conformational changes. Subsequently, site‐directed mutagenesis guided by enhanced sampling molecular dynamics simulations revealed the multifaceted role of the loop element within the half‐open active pocket of IDO throughout the catalytic cycle, encompassing conformation regulation of the active pocket, substrate anchoring, and participation in substrate/product transport, and identified the key residues involved in these functions. These findings strengthen functional insights into the loop elements composing the αKGD half‐open active pocket and highlight the potential of protein structuromics for elucidating structure‐function relationships across diverse protein families.

## 2 Results

### 2.1 The PF10014 Family Exhibits High Structural Conservation Despite Low Sequence Conservation

**FIGURE 2:**
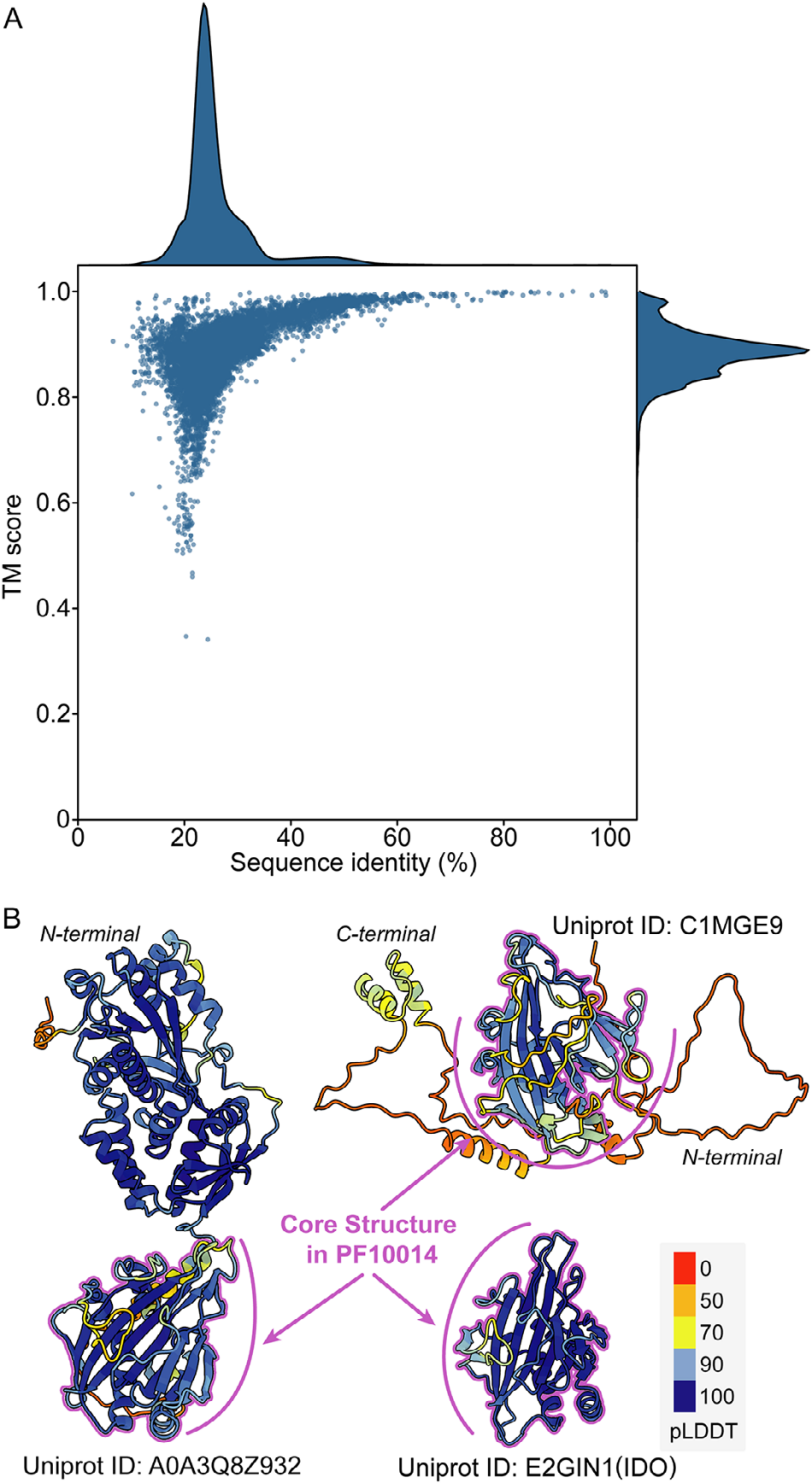
Sequence and structural similarity across the PF10014 family. (A) TM‐score, indicative of structural similarity, versus sequence identity among PF10014 family members. The larger TM‐score in the result of structural pairwise comparisons was selected for analysis. (B) Schematic structures of 3 representative PF10014 family members with diverse N‐/C‐terminal domains. Residues are colored based on confidence (pLDDT), and their core structural domain are outlined in purple.

Sequence‐ and structure‐based bioinformatics analyses represent two typical approaches for investigating protein function. Here, the PF10014 family, to which IDO belongs and which is characterized by an active pocket containing only a single loop [25], was selected as the focus, and their sequence and structural conservation were first determined. On the sequence level, protein sequences belonging to PF10014 were extracted from the InterPro database [26] (Uniprot IDs detailed in Table S1). Sequence alignment revealed that sequence identity for nearly all sequence pairs fell below 40%, with more than 90% pairs ranging between 15% and 35%, indicating considerable sequence differences among PF10014 family members. Turning to the structural aspect, structural models corresponding to these sequences were obtained from the AlphaFold database (AFDB) when available or were predicted using the AlphaFold‐based ColabFold in cases where predicted structures were absent from the AFDB. Subsequent pairwise comparisons of these structures by TM‐align indicated significant structural conservation among family members, with more than 99% of the structure pairs exhibiting TM‐scores between 0.6 and 1 (Figure 2A). Further structural visualizations demonstrated that these members possess domains analogous to those in the IDO structure, although the N‐terminal or C‐terminal domains of these proteins differ greatly among the enzymes in PF10014 family (Figure 2B). Overall, these findings suggest that the structures of PF10014 family members are more conserved than their sequences, and that IDO contains the core structural domains conserved in this family and thus represents a typical family member.

### 2.2 Protein Structuromics‐Based Identification of the Structural Features of αKGDs

**FIGURE 3:**
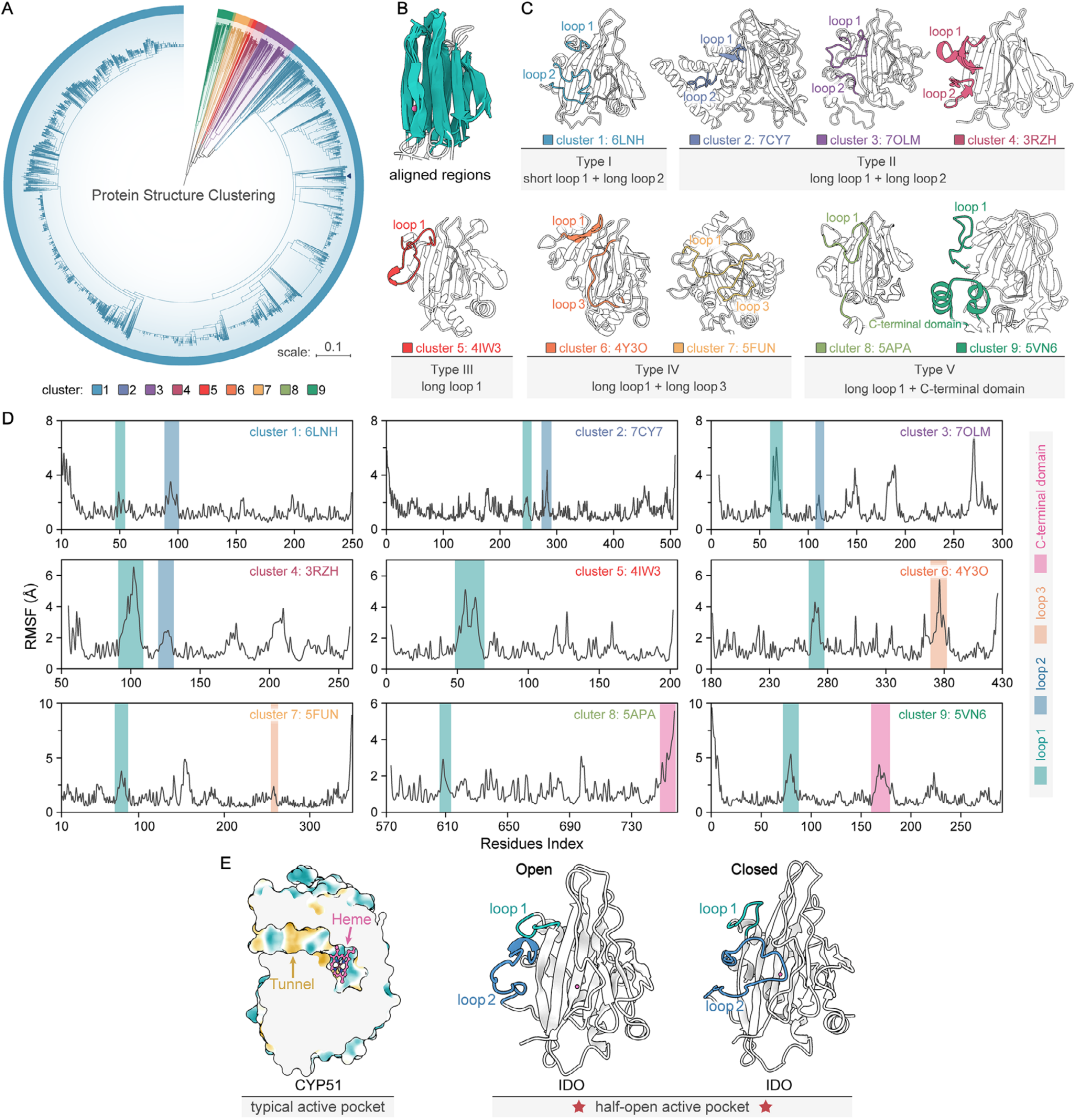
Protein structure clustering reveals structural features of the half‐open active pocket in αKGDs. (A) Structural classification of αKGDs. Members of the PF10014 family, to which IDO belongs, along with IDO homologs, were pooled for structural clustering analysis. Proteins were divided into different structural clusters based on protein structure comparison and are labeled by color. The steel blue triangle indicates IDO. (B) Schematic representation of the aligned structures. For clarity, only a few structures are shown, and unaligned structural domains outside of the conserved DSBH fold are hidden. The conserved DSBH fold and Fe^II^ center are colored green and pink, respectively. (C) Structures of representatives of the 9 clusters. Loops constituting the active pocket are colored distinctly across different structures, whereas the conserved β‐strand comprising the active site is uniformly colored in dark grey across all structures. The grey text below the structures denotes active pocket types distinguished by different structural features of loops. (D) Root mean square fluctuation (RMSF) analyses of 9 representative structures, each labeled with its corresponding PDB ID. The loops forming the active pocket are highlighted in color. (E) Structural features of different active pocket types. P450‐type enzymes are represented by CYP51 (PDB: 2WUZ), which is shown as a hydrophobic surface model, with the haem shown in pink as a ball‐and‐stick model. IDO (PDB: 6LNH) is shown in cartoon, with loop 1, loop 2, and ferrous ions colored green, blue, and pink, respectively. The open and closed conformations of IDO were extracted from previous work [15].

The above findings confirmed high structural conservation within the PF10014 family. To further investigate the structural similarities and differences between this family members and other αKGDs, we implemented a protein structuromics [24] analysis approach that integrates AI‐assisted protein structure prediction, structural alignment, and clustering methodologies to classify the structures and describe their relationships. Specifically, the structures of all PF10014 family (to which IDO belongs) members were predicted using AlphaFold as described in the previous section, while homologous αKGD structures of IDO were retrieved through Foldseek. This search revealed 495 structures that belong to the αKGD superfamily but are not currently listed in the PF10014 entry (PDB IDs detailed in Table S1). Then, the structures obtained from two different approaches were merged into a single protein set, totaling 4,981 structures, and subjected to pairwise structural comparisons via TM‐align, generating a distance matrix reflecting structural similarities. Next, on the basis of this distance matrix, the αKGD structures were classified into 9 distinct structural clusters using the unweighted pairwise grouping method and arithmetic mean (UPGMA) (Figure 3A). Notably, all members of the PF10014 family were grouped into the same large cluster (Cluster 1). The TM‐scores for all structural comparisons primarily range from 0.45 to 0.65. Specifically, structural similarity among the 9 clusters was attributed primarily to the conserved DSBH fold inherent to the αKGD superfamily (Figure 3B). In contrast, structural differences among clusters were mainly localized to the region opposite the active site, where loops are typically enriched, with 1–3 loops of variable lengths present across different structures (Figure 3C, colored regions). These loops, together with the β4 involved in Fe^II^ chelation (Figure 3C, dark grey region), constitute the active pocket.

When focusing on the loops comprising the active pockets, αKGDs were categorized into 5 distinct structural types based on the loop composition of their active pocket (Figure 3C). Molecular dynamics (MD) simulations of representative structures revealed that these loops were more flexible than their respective global structures (Figure 3D). Given the importance of loop dynamics for the evolution of enzymatic function [16, 17], these highly flexible loops constituting the active pocket were hypothesized to be related to the catalytic function of the αKG superfamily. To further investigate this possibility, the prevalence of residue missing within loops constituting the active pocket was surveyed across crystal structures of IDO's αKGD homologs. Statistical analysis revealed that a substantial proportion (42%) of the experimental structures exhibited missing residues in these loops (Figure S1A), and enzymes exhibiting such residue missing were associated with various reactions (Figure S1B). These statistical findings further hint at the potentially vital role of the loops comprising the active pocket in αKGD catalysis.

When the perspective shifts to the entire active pocket, its structural feature differs from those typically observed. In detail, the active site of an enzyme is typically buried deep inside the protein architecture and connected to the external environment through one or more pathways [27]. For example, in another famous iron‐containing enzyme, cytochrome P450, the haem active site is entirely encompassed inside the structure and is accessible only by a single tunnel (Figure 3E). In contrast, the active pocket structure of IDO markedly deviates from this typical feature (Figure 3E). Specifically, the region anchoring the Fe^II^ center exhibits rigidity (Figure 3D), whereas loop 2 undergoes conformational changes to govern active pocket accessibility (Figure 3E and Figure S2). Concurrently, owing to the amorphous nature of the active pocket architecture, IDO lacks the well‐defined tunnel analogous to that observed in P450 enzymes. This architecture, characterized by complementary rigid and flexible components, is defined as a half‐open active pocket. Given the analogous structural features of the active pockets across different cluster members (Figure 3C), here, we suggest that the half‐open active pocket configuration is common to αKGDs.

### 2.3 Identification of “Gate Buckle” Residues in the Half‐Open Active Pocket Conformation

Because the loop comprising the half‐open active pocket was suggested to function through conformation change, the capability of conventional molecular dynamics (cMD) simulations and the enhanced‐sampling approach velocity‐scaling replica exchange molecular dynamics (vsREMD) simulations to explore the conformational distribution of IDO was comparatively evaluated, with the aim of identifying key residues regulating these conformational changes. The results (Figure S3) showed that the conformational space sampled by cMD was limited to an Rg range of 1.71–1.78 nm and an RMSD range of 0.05–0.35 nm. In contrast, vsREMD sampled a substantially broader conformational distribution, with Rg values ranging from 1.72 to 1.84 nm and RMSD values ranging from 0.07 to 0.60 nm. These results indicated that vsREMD was more suitable for exploring the large‐scale conformational changes associated with the loop element.

To further evaluate the sampling efficacy of vsREMD for IDO, the temperature history and potential energy distribution of the replicas were initially extracted. The statistical results demonstrated that the temperature history of the replicas effectively covered the predefined temperature range (Figure S4A). Subsequently, the potential energy of each replica exhibited typical Boltzmann distributions, displaying both adequate distinction and visible overlap (Figure S4B), with an average acceptance probability of 27.5% between the replicas.

Additionally, the free‐energy landscapes of the root mean square deviation (RMSD) versus the radius of gyration (R_g_) were subsequently calculated based on the vsREMD simulation trajectories. RMSD and R_g_ were selected because the major conformational changes of IDO were predominantly associated with the loop element forming the half‐open active pocket, whereas the remaining structural regions remained relatively stable. Moreover, conformational changes in the loop significantly affect the overall structural compactness of IDO, allowing RMSD and R_g_ to more directly capture the loop‐dependent conformational distribution. Then, the free‐energy landscapes at varying temperatures revealed a progressive expansion in the distribution range of the RMSD and R_g_ values with increasing temperature (Figure S4C), indicating that IDO could cross energy barriers between distinct conformational populations, thereby enabling comprehensive exploration of the conformational space. These results demonstrate that the vsREMD parameters were set appropriately to allow adequate sampling of the conformational space for IDO.

**FIGURE 4:**
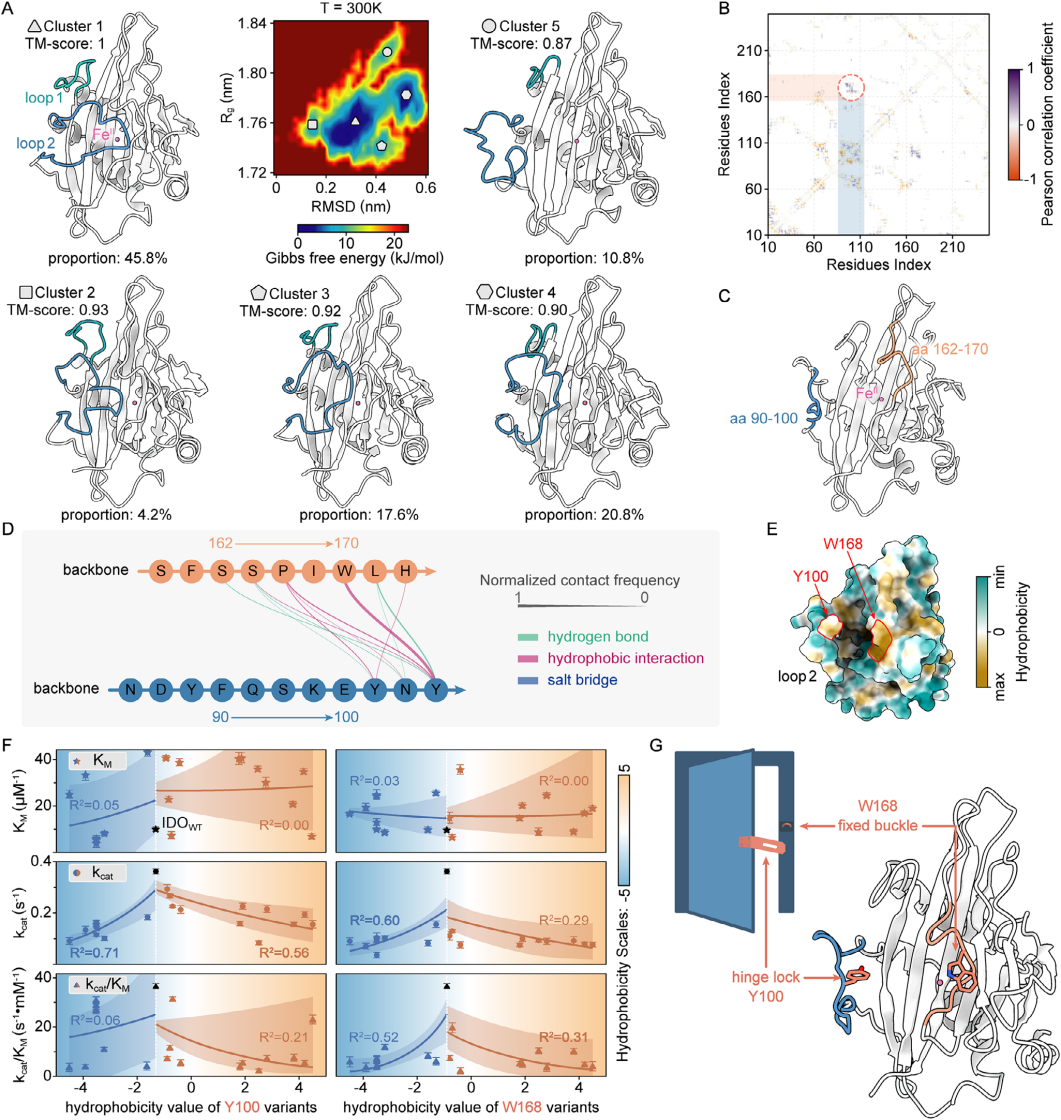
Computational identification and functional characterization of “gate buckle” residues. (A) Free‐energy landscape and corresponding clustered structures of IDO conformations at 300 K. Free‐energy landscape as a function of RMSD and R_g_ from the vsREMD trajectory, where conformational clusters are labeled with different grey shapes. The representative structures of conformational clusters are displayed as cartoons, and loop 1 and loop 2 are shown in light sea green and steel blue respectively. (B) Correlation between the inter‐residue distance and R_g_. Correlation analysis was performed with CONAN [29]. The regions exhibiting a strong positive correlation are highlighted with a pink circle, the data for residues 90–100 and 162–170 are indicated as light blue and light orange bars, respectively. (C) Cartoon representation of the IDO structure, with residues 90–100 in steel blue, residues 162–170 in salmon, and the ferrous ion in pink. (D) Contact frequencies between the 90–100 and 162–170 regions, extracted from the vsREMD trajectory. Normalized contact frequencies are represented by the width of the lines, with hydrogen bonds indicated in green, hydrophobic interactions in purple, and salt bridges in blue. Salt bridges were not detected between these two regions. (E) The hydrophobic surface of the IDO structure. (F) Exponential fitting of kinetic parameters to the hydrophobicity of variants at positions 100 and 168. The hydrophobicity data for the amino acids are shown in Table S2. (G) Structure‒function relationships of “gate buckle” residues. Loop 2 is colored steel blue, and β4 is colored salmon. The “gate buckle” residues Y100 and W168 are shown in stick representation and colored dark salmon.

The conformational landscape of IDO was analyzed in detail to identify critical residues regulating conformational changes in the active pocket loop. At 300 K, the IDO conformations were grouped into 5 distinct clusters (Figure 4A), with the primary distinguishing feature being the conformation of loop 2, which forms the half‐open active pocket (Figure 4A and Movie S1). Among these, Clusters 1, 2, and 3 exhibited few conformational differences, representing a closed conformation of loop 2, with Cluster 1 dominating the population. In contrast, Clusters 4 and 5 primarily corresponded to open loop 2 conformation (Figure 4A). Pearson correlation analysis revealed that distances between residues 90–100 (part of loop 2) and 162–170 (part of β4) were significantly correlated with R_g_, a parameter characterizing structural compactness (Figure 4B,C). This finding indicates that conformational changes in loop 2 are closely associated with alterations in the spatial relationship between residues 90–100 and 162–170. Statistical analysis of the noncovalent interactions further revealed that Y100 within residues 90–100 and W168 within residues 162–170 presented the highest contact frequency among all residues in the two regions (Figure 4D), suggesting the important role of these specific residues in loop 2 conformational changes.

Given the substantial contribution of hydrophobic interactions, hydrophobicity analysis was performed to provide further insights into these two regions. The region from residues 90–100 was predominantly hydrophilic, with Y100 exhibiting clear hydrophobicity. In contrast, the region comprising residues 162–170 contained a distinctly hydrophobic segment, with W168 near the active site contributing substantially to this hydrophobicity (Figure 4E). The hydrophobicity analysis results, together with the observed conformational changes of the loop forming the half‐open active pocket (Figure 4A), suggest that the hydrophobicity of Y100 and W168 is associated with conformational changes within the half‐open active pocket.

To further investigate the specific effects of residues Y100 and W168 in the conformational changes in loop 2, single‐site saturation mutagenesis was performed at Y100 and W168. After confirming the expression and purification of the mutants (Figure S5), kinetic parameters were determined for the Y100 and W168 variants. The hydrophobicity of the mutated residues at these two positions was subsequently fitted against the corresponding kinetic parameters (*K*_M_, *k*_cat_, *k*_cat_/*K*_M_), as hydrophobic interactions in critical regions have been reported to modulate the activation or inactivation conformations of enzyme function [28]. Exponential fitting revealed no correlation between the hydrophobicity of residues at positions 100 and 168 and their *K*_M_ values (Figure 4F). In contrast, the hydrophobicity of both sites, especially residue 100, showed a strong correlation with the respective *k*_cat_ values. Specifically, with increasing or decreasing hydrophobicity, the *k*_cat_ values of the variants at both positions displayed an exponential decline (Figure 4F). Additionally, the hydrophobicity of variants, primarily at residue 168, exhibits a certain negative correlation with *k*_cat_/*K*_M_ (Figure 4F), that is likely attributable to contributions from *k*_cat_.

Integrating the extensive conformational changes of loop 2 with the little positional fluctuations of β4 (Movie S1) and the fitting results (Figure 4F), loop 2 could be conceptualized as a “gate”, with β4 serving as its “frame”. In this concept, Y100 and W168 function analogously to a “gate buckle”, wherein Y100 serves as the hinge lock, and W168 acts as the fixed buckle (Figure 4G). When the hydrophobic interaction between Y100 and W168 is weakened, the conformational freedom of loop 2 increases, making it difficult to maintain the closed conformation even though it can be easily accessed. This hinders stable substrate binding and efficient catalysis. Conversely, strengthening the hydrophobic interaction between Y100 and W168 results in a tightly locked “gate buckle”, reducing the conformational freedom of loop 2. While this benefits the closed conformation, it simultaneously hampers the rapid turnover of substrates and products, thereby lowering overall catalytic efficiency.

In summary, either weakening or strengthening of the hydrophobic interactions between the “gate buckle” residues Y100 and W168 resulted in markedly reduced catalytic efficiency. Their interactions must be maintained at an appropriate strength to ensure a subtle balance between the open and closed conformations of loop 2 in the half‐open active pocket, thereby supporting the efficient catalytic cycle as observed in wild‐type IDO.

### 2.4 Anchoring Residues in Loop 2 Regulate Substrate Recognition and Catalytic Activity

The results from the preceding section indicate that the conformational changes of loop 2 modulate *K*_M_, suggesting its involvement in substrate recognition and anchoring. To test this hypothesis, potential substrate‐anchoring residues within loop 2 were identified and verified through computational simulation and mutation studies.

**FIGURE 5:**
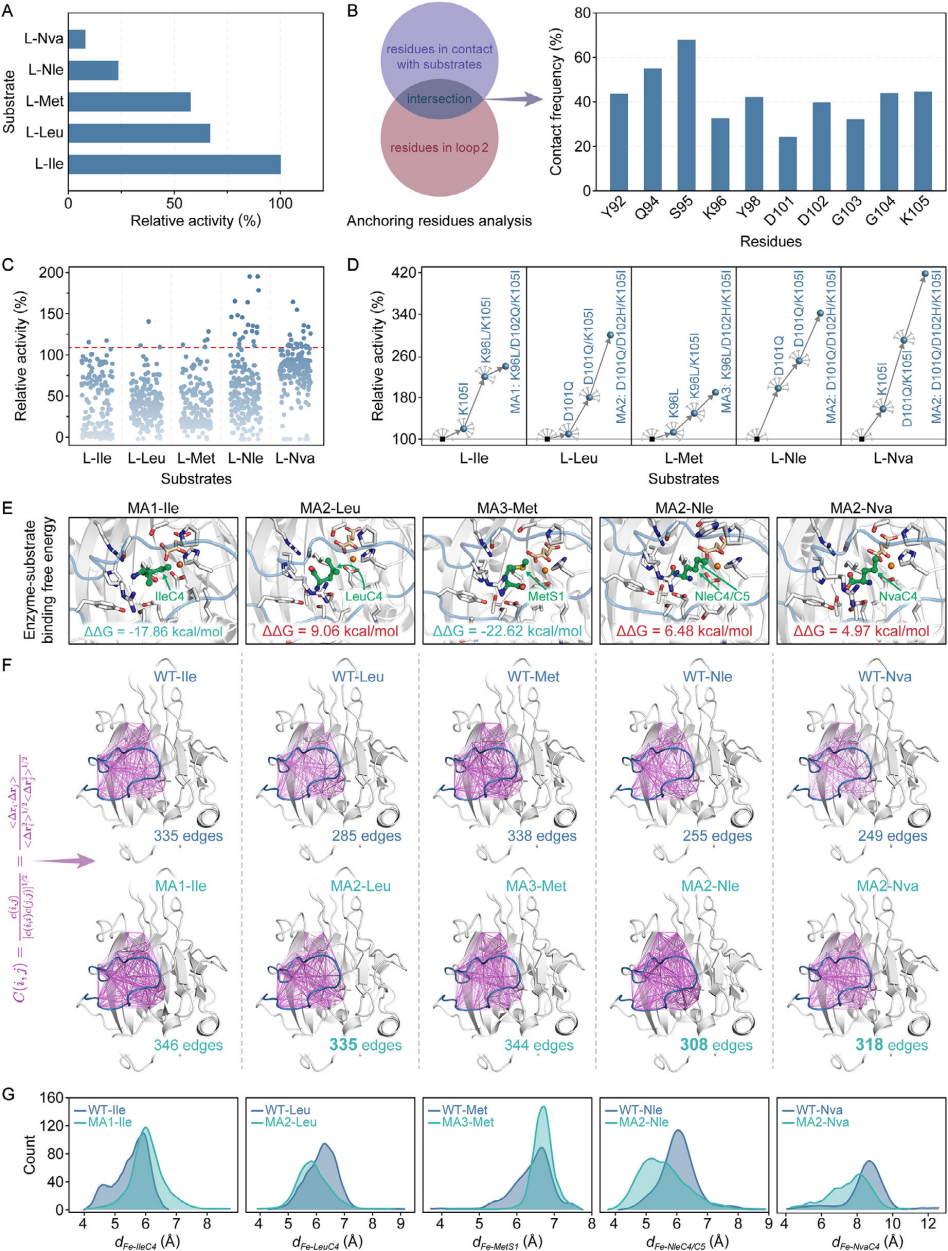
Mutation of substrate‐anchoring residues in loop 2 enhances the catalytic activity of IDO. (A) Hydroxylation activity of IDO toward different substrates. L‐Nva: L‐norvaline; L‐Nle: L‐norleucine; L‐Met: L‐methionine; L‐Leu: L‐leucine; L‐Ile: L‐isoleucine. (B) Identification of anchoring residues within loop 2. Contact analysis was performed using PyContact [31], and the contact frequency is defined as the proportion of frames forming contacts to the total number of frames. (C) Activity screening of single‐site saturation variants at 10 selected sites. (D) Iterative mutagenesis toward different substrates. (E) Binding mode and binding free energy of enzyme‒substrate complexes. Loop 2 is shown as a sky blue cartoon, and the ligand is shown as a green ball‐and‐stick model, with the attacked atom indicated by an arrow. (F) Cross‐correlation networks within 5 Å of the substrate in different complexes. The cross‐correlation coefficient was calculated from the formula [32] on the left and is shown as the purple line in the cartoon. (G) Distance statistics between Fe^II^ and the attacked substrate atom in different complexes.

First, substrate analysis confirmed that IDO can hydroxylate 5 hydrophobic aliphatic amino acids (L‐Nva: L‐norvaline; L‐Nle: L‐norleucine; L‐Met: L‐methionine; L‐Leu: L‐leucine; L‐Ile: L‐isoleucine, Figure S6), among which the highest catalytic activity was observed toward L‐Ile (Figure 5A), with a specific activity reaching 4.95 U·mg^−1^. Next, the substrates were docked into the closed conformation of IDO (Figure 4A, Cluster 1), and all of the resulting complexes exhibited a “handlebar” binding mode, whereby the substrates were predominantly bound via strong interactions between their amino/carboxyl groups and the anchoring residues (Figure S7). Subsequent MD simulations were performed on 5 complexes, from which the residues that interact with substrates were extracted. The intersection of these interacting residues and those in loop 2 yielded a set of 10 potential anchoring residues. Each filtered residue displays a contact frequency with the substrates exceeding 20% (Figure 5B), suggesting their significant contribution to substrate anchoring.

Site‐saturation mutagenesis of these 10 anchoring residues revealed reduced catalytic activity for the vast majority of the variants (Figure 5C), confirming the importance of these anchoring residues in substrate binding. Notably, a few variants presented increased activity, suggesting the potential for optimizing substrate binding. Consequently, when 110% relative activity was used as a screening threshold (Figure 5C, red dashed line), 20 distinct single‐site variants (Q94R, K96L, K96I, K96F, D101M, D101T, D101Y, D101N, D101Q, D102T, D102Y, D102N, D102Q, D102R, D102H, G103T, G104R, K105V, K105I, and K105Y) with increased activity were identified. Thus, iterative mutations were conducted on these identified variants to further optimize the enzyme activity toward different substrates (Figure 5D). Specifically, in the first round, the most significant enhancements in activity were observed for K96L toward L‐Met, D101Q toward both L‐Leu and L‐Nle, and K105I toward L‐Ile and L‐Nva. In the second round, these 3 optimal single‐site variants were each combined with the remaining single‐site variants. Of the resulting double‐site variants, the best performing variants were K96L/K105I and D101Q/K105I, with the former showing the greatest improvement in activity toward L‐Ile and L‐Met, and the latter exhibiting the highest activity increases toward L‐Leu, L‐Nle, and L‐Nva. In the third round, the increases in activity of 2.41‐fold for K96L/D102Q/K105I (MA1) toward L‐Ile; 3.02‐, 3.42‐, and 4.16‐fold for D101Q/D102H/K105I (MA2) toward L‐Leu, L‐Nle, and L‐Nva; and 1.91‐fold for K96L/D102H/K105I (MA3) toward L‐Met relative to the activity of the wild‐type enzyme were achieved. No further improvements were observed in the fourth round of iterative mutagenesis, indicating that these triple‐site variants within the sequence of loop 2 had achieved optimized activity for these 5 substrates.

The kinetic parameters of the purified optimal triple‐site variants (Figure S8) were characterized to determine whether the increased activity was due to improved substrate binding or turnover. Specifically, variants incorporating K96L (MA1 and MA3) exhibited improved catalytic efficiency, which was predominantly attributed to reduced *K*_M_ values (Table S3). Whereas the increased activity of the variant harboring D101Q (MA2) principally originated from an elevated turnover number (*k*_cat_, Table S3). Computational analyses revealed that the decreases in *K*_M_ for MA1 and MA3 compared to the wild‐type enzyme were due mainly to optimization of the binding free energy, with ΔΔG values of −17.86 and −22.62 kcal/mol, respectively (Figure 5E). However, the ΔΔG of MA2 was greater than that of the wild type (Figure 5E), indicating that its enhanced catalytic performance was not driven by the binding free energy. Thus, the cross‐correlation networks of enzyme‒substrate complexes were investigated, because enzymes adjust their dynamic networks to access productive conformations upon binding substrates [30]. The results revealed a marked increase in both the numbers and their correlation of edges within the cross‐correlation network among residues located within 5 Å of the substrate in the MA2 variant compared to the wild‐type (Figure 5F). This suggested that the residue mutations in the MA2 variant enhance the flexibility of its half‐open active pocket, thereby facilitating substrate access to productive conformations. This result was further confirmed by the most frequent distance (MFD) statistic: the MFD values between Fe^II^ and the attacked atoms of substrates were shorter in MA2 than those in the wild‐type enzyme (Figure 5G), implying an increased probability of Fe^II^ attacking substrates. These results are consistent with the increased *k*_cat_ for MA2 compared with the wild‐type enzyme (Table S3).

Overall, mutations of most anchoring residues within loop 2 in IDO resulted in diminished activity, whereas iterative mutagenesis of the activity‐enhanced variants optimized their catalytic efficiency toward different substrates. Specifically, among these optimal variants, the increases in catalytic activity of MA1 and MA3 were mainly driven by the increased binding free energy, whereas that of MA2 was mainly attributed to an increased probability of attacking the substrate supported by enhanced cross‐correlation networks. These results provide a functional understanding of the role of loop 2 in substrate anchoring.

### 2.5 Involvement of Tunnel Residues in Loop 2 in Molecular Transport

**FIGURE 6:**
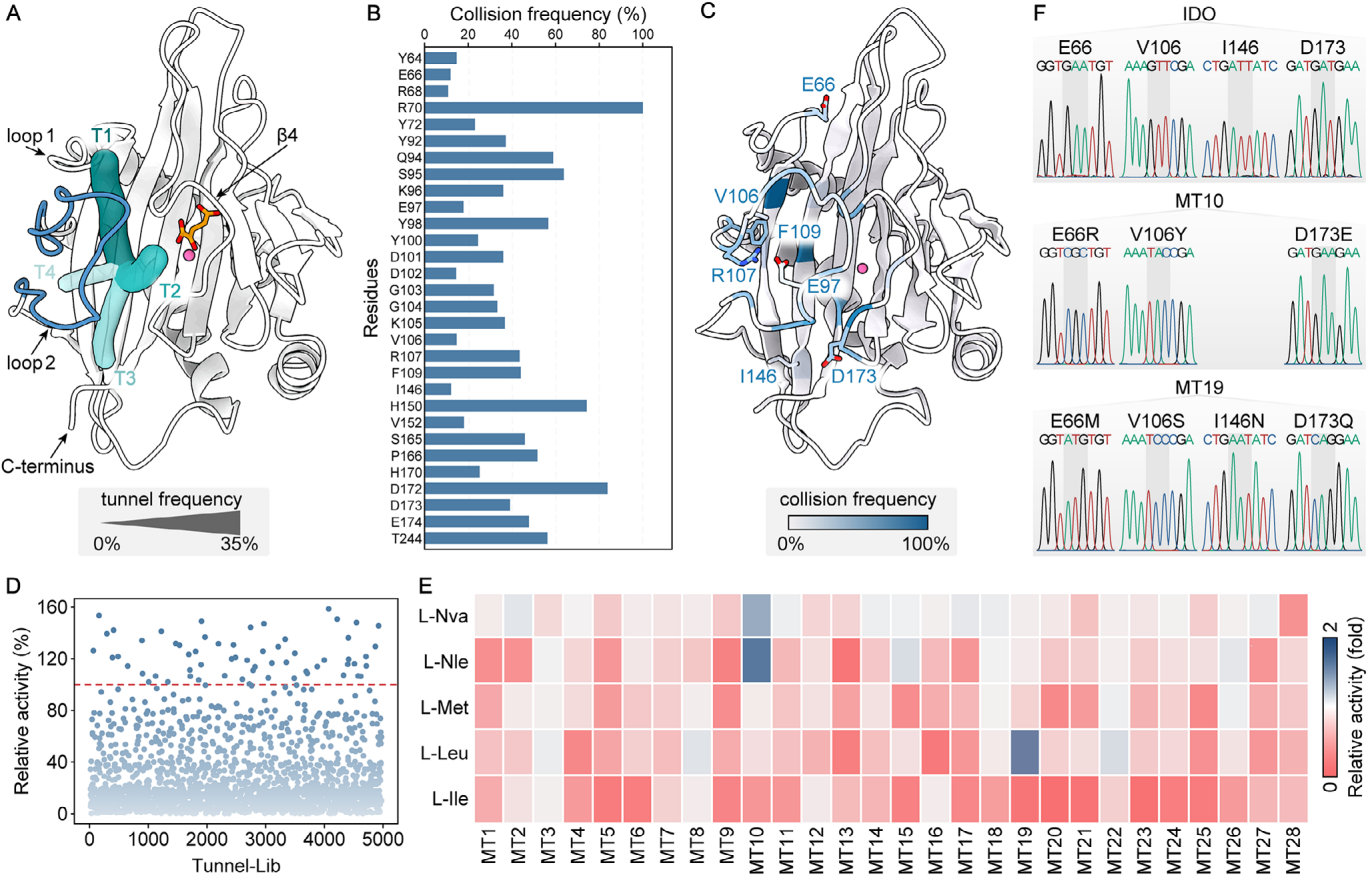
Tunnel‐associated residues in loop 2 are involved in the regulation of molecular transport. (A) Possible tunnels for molecular transport between the active site of IDO and the external space shown as surface mode. Loop 2 is colored in steel blue in the structural representation. Tunnel frequency is defined as the percentage of repeated RAMD trajectories in which the small molecule successfully exits the active site through a specific tunnel. (B) Collision frequency between residues in IDO and substrates from the RAMD trajectories of all protein–ligand complexes. (C) Spatial arrangement of candidate mutation sites among the tunnel residues in IDO. (D) High‐throughput screening of the combinatorial mutation library Tunnel‐Lib. The combinatorial mutation information of Tunnel‐Lib is detailed in Table S4. 5000 variants were screened to cover the theoretical number of variants (1728) in Tunnel‐Lib as much as possible. (E) Activity verification of the dominant mutants. (F) Identification of the mutations in MT10 and MT19 by DNA sequencing.

The enzymatic catalysis process encompasses substrate entry, a chemical reaction, and product release [33, 34]. Given that loop 2 governs half‐open active pocket accessibility through extensive conformational changes (Figure 4A), resulting in the absence of a well‐defined substrate/product tunnel in IDO, loop 2 is also speculated to potentially participate in the transport of substrates or products. To explore potential molecular transport pathways as comprehensively as possible, random accelerated molecular dynamics (RAMD) simulations were performed on enzyme‐substrate/product complexes to enhance the sampling of ligand transport events, with IDO in 5 distinct conformations selected as the receptor (Figure 4A and Figure S9). The RAMD simulation trajectories of all protein–ligand complexes revealed four possible tunnels to support substrate/product transport between the active site and the exterior (Figure 6A). Specifically, tunnels oriented toward loop 1 (T1) and directed toward the apex of loop 2 (T2) were predominant. Moreover, tunnels connecting the active site and the C‐terminus (T3) and traversing directly through loop 2 from the active site (T4) were also identified. Notably, these potential tunnels all involve residues within loop 2.

Since external forces are applied to the ligand in the protein–ligand complexes during RAMD simulations, the distance between the ligand and residues in IDO may have become excessively close (e.g., <3 Å), forming repulsive forces that resemble collision phenomena at the macroscopic scale. To evaluate the likelihood of such a situation, the collision frequencies of the tunnel residues were quantified from the RAMD trajectories of all protein–ligand complexes. Using a 10% collision frequency threshold, 30 potential tunnel residues were filtered, including most of the residues in loop 2 (Figure 6B). Among the residues within loop 2 involved in tunnel formation, E97, V106, R107, and F109 were selected as candidate sites for mutagenesis after excluding those that had been studied in the previous sections (Figure 6C). Additionally, residues E66, I146, and D173, which spatially co‐form tunnels with the aforementioned candidate residues, were also considered as mutagenesis sites (Figure 6C).

Direct detection of product formation in IDO‐catalyzed reactions by HPLC is time‐consuming and thus challenging for high‐throughput applications. To balance screening efficiency and experimental throughput, a combinatorial mutation library targeting these candidate sites (Tunnel‐Lib) was designed (Table S4). Concurrently, high‐throughput screening of Tunnel‐Lib was performed using a luminescence‐based assay targeting succinic acid generation, as the product and by‐product (succinic acid) are generated in equimolar stoichiometry in the IDO‐catalyzed reaction (Figure S6). The high‐throughput screening, conducted following the workflow shown in Figure S10, revealed a substantial decrease in the relative catalytic activity of most variants (Figure 6D), underscoring the functional significance of these residues. Moreover, 60 variants exhibited enhanced catalytic activity relative to that of wild‐type IDO (Figure 6D, red dashed line); among these variants, 28 variants demonstrated a >1.2‐fold improvement. Further activity validation of the purified 28 filtered variants by HPLC revealed that most variants did not replicate the increase in relative activity (which may be mainly due to the use of a crude enzyme solution during high‐throughput screening), with only two variants exhibiting a confirmed increase in relative activity (Figure 6E). Specifically, MT10 displayed 1.73‐ and 1.43‐fold increased activity toward L‐Nle and L‐Nva, respectively, whereas MT19 achieved a 1.57‐fold increase in activity toward L‐Leu. Determination of the kinetic parameters indicated that the improved catalytic activities of both MT10 and MT19 were attributable to an increase in turnover number (*k*_cat_, Table S5). Sequencing identified MT10 as E66R/V106Y/D173E and MT19 as E66M/V106S/I146N/D173Q (Figure 6F). As the majority of substitutions occurred at residues distant from the substrate (Figure S7), the increased *k*_cat_ observed in these two preferred variants was thought to be associated with optimization of molecular transport. Altogether, the substantial number of negative mutations involving residues within loop 2 distant from the substrate (Figure 6D), coupled with the result that both preferred variants harbor substitutions at position V106 within loop 2 (Figure 6F), collectively support the proposition that loop 2 participates in molecular transport.

## 3 Conclusion

The active pocket of the αKGD superfamily typically incorporates loops, yet these loops exhibit diverse functions across different reports. In this work, the structural features of the active pocket within the αKGD superfamily were systematically delineated through protein structuromics. Specifically, members of the αKGD superfamily were confirmed to be structurally conserved, attributable to the family‐conserved DSBH fold. Furthermore, extensive structural comparisons also revealed that the active pocket architecture within this superfamily is relatively conserved. Since the loops constituting the active pocket govern its accessibility through conformational changes (Figure 4A and Figure S2 and Movie S1), this architecture was defined as half‐open active pocket and proposed as a characteristic structural feature of the αKGD superfamily. Moreover, mutagenesis experiments guided by enhanced sampling simulations unveiled the multifaceted roles of loop 2 within the half‐open active pocket of the IDO, a member of the αKGD superfamily, including (1) governing the open and closed states of the half‐open active pocket through conformational changes, (2) underlying substrate recognition and anchoring, and (3) participating in substrate/product transport. These results indicate that the loop‐associated half‐open active pocket should not be viewed solely as a flexible structural element for substrate binding. Rather, it functions as an integrated catalytic module that couples conformational regulation, substrate positioning, and molecular transport.

Notably, a similar half‐open configuration was described two decades ago in studies of another metal‐dependent enzyme, peptide deformylase [35]. Intriguingly, both αKGDs and peptide deformylase serve as critical metabolic enzymes in living organisms. For instance, proline hydroxylase, a member of αKGDs, rapidly executes hydroxylation reactions in the oxygen‐sensing pathway to maintain normal cellular function [36], whereas the latter performs deformylation of nascent peptide chains within the narrow temporal window of protein co‐translational processing to ensure subsequent protein maturation and functionalization [37]. Consequently, a question meriting further exploration is whether the half‐open active pocket represents one of the structural foundations underlying the capacity of these enzymes to rapidly execute metabolic tasks.

Additionally, research into the structure‐function relationships of the αKGD superfamily prior to the era of AI‐based protein structure prediction has been limited by the absence of high‐throughput methods capable of comprehensively mapping the structural landscape of these enzymes. Protein structuromics overcomes this challenge by leveraging large‐scale AI‐predicted structural data to uncover family‐wide features, complementing sequence‐based approaches. This methodology can prove particularly effective in cases of significant sequence divergence, such as PF10014 family, where insights into the structural landscape enhance understanding of structure–function relationships. Similar protein structuromics analyses have been applied to discover gene editing tool enzymes [38, 39, 40] and understand the functions of viral proteins [41], highlighting the potential of protein structuromics for protein resource mining, functional evolution of protein, and protein engineering. Nevertheless, current structural comparison methodologies are primarily applied at the level of global protein structure to infer structural classification relationships, and further development of alignment algorithms is required to address diverse structural relationship classification needs, such as the local structural comparison and classification of half‐open active pockets studied in this work.

In summary, this work identified a conserved half‐open active pocket architecture within the αKGD superfamily and elucidated the multifaceted functions of the loop constituting this structural motif. These findings expand the current understanding of the structure‐function relationships within the αKGD superfamily and underscore the potential of protein structuromics in elucidating such relationships, while promising to facilitate the development of αKGD superfamily‐based biocatalytic platforms for C−H functionalization.

## 4 Experimental Section

### 4.1 Protein Structuromics Analysis: (1) Structure Extraction and Prediction

The Pfam entry for the family to which IDO belongs is PF10014. By querying PF10014 in the InterPro database [26], a total of 4486 protein sequences belonging to this family were extracted (UniProt IDs detailed in Table S1). The corresponding 3D structures were obtained on the basis of the extracted protein sequence information. Specifically, if the experimentally determined or computationally predicted 3D structures were available in the PDB [42] or AFDB [43], they were extracted from these databases. Otherwise, the remaining structures were predicted using AlphaFold‐based ColabFold [18, 44]. In addition, Foldseek [21] was employed to search structural homologs of IDO against the PDB database, yielding 495 hits (PDB IDs detailed in Table S1). Collectively, a total of 4981 structures were compiled from two different approaches.

### 4.2 Protein Structuromics Analysis: (2) Structure Alignment and Structure‐based Classification

Pairwise structural alignments of the collected protein structures were executed using the TM‐align algorithm [22]. The structural similarity matrix of all proteins was constructed on the basis of the structural alignment results (taking the *max*[TM‐score]), which was subsequently normalized using the min‒max method. Furthermore, the structural similarity matrix was clustered by Unweighted Pair Group Method with Arithmetic mean (UPGMA) to construct structure‐based relationships and visualized using tvBOT (https://www.chiplot.online/tvbot.html) [45].

### 4.3 Conventional MD Simulations

The crystal structure of IDO (PDB ID: 6LNH) was extracted from the PDB, and the missing residues were reconstructed using MODELLER [46]. The cofactor αKG and substrate were subsequently docked stepwise into the repaired IDO structure using AutoDock Vina [47].

For the docked complex structure, the protein was protonated using the *H*++ tool [48], and ligand topology parameters were generated using ACPYPE [49]. In MD simulations, the AMBERff14SB force field [50] was employed to describe the atomic dynamics within the system. A box with periodic boundary conditions in the X, Y, and Z dimensions was constructed, ensuring a minimum distance of 1.2 nm between the periodic boundaries and protein atoms. The system was solvated using the TIP3P water model, with counterions (Na^+^ and Cl^−^) added to neutralize the total charge. After system preparation, energy minimization was performed using the steepest descent method to eliminate unreasonable atomic contacts. NVT equilibration was subsequently conducted at 300 K, with the LINCS algorithm applied for bond constraints on H‐bonds, particle mesh Ewald for long‐range electrostatic effects treatment, and the V‐rescale method for temperature coupling. This was followed by NPT equilibration at 300 K, specifically utilizing the Berendsen method for pressure coupling, while the parameters for bond constraints, long‐range electrostatic effects, and temperature coupling remained identical to those employed during the NVT equilibration step. Upon completion of both equilibration phases, production MD simulations were executed for 100 ns at 300 K. In this step, the simulation time step was set at 2 fs, with trajectory files written every 1000 steps. Each simulation was performed in triplicate, and all simulation‐related analyses were averaged. All cMD simulations were executed using the GPU version of GROMACS 2020.5.

### 4.4 VsREMD Simulations

The temperature distribution for vsREMD [51] was set from 300 to 400 K, with exchanges attempted at intervals of 1000 steps between adjacent replicas to achieve an average exchange probability of ∼30%. On the basis of these two parameters, the desired temperature distribution was generated using the temperature predictor [52], with a total of 16 replicates (Table S6). For each replica, the corresponding initial configuration was prepared according to the temperature distribution. The parameters and settings for energy minimization and the NVT and NPT steps remained consistent with those employed in the cMD simulations. The parallel production simulation was conducted for 100 ns. All vsREMD simulations were performed using a modified version of GROMACS 5.1.4 [51].

### 4.5 RAMD Simulations

In the RAMD simulations, the protein‒ligand complex topology was prepared as described about for cMD simulations, and the parameters and settings for energy minimization and the NVT and NPT steps were consistent with those employed in cMD simulations. During the production simulation phase, a force of 14 kcal·mol^−1^·Å^−1^ was applied to the ligand, with its direction chosen at random. Every 100 fs of simulation time, the displacement of the ligand's center of mass was evaluated, wherein the force direction was preserved if this displacement exceeded 0.025 Å or otherwise randomly altered. The RAMD simulation was terminated once the displacement of the ligand's center of mass exceeded 40 Å from its initial position. To ensure sufficient sampling, each complex was subjected to 16 independent simulations, each initiated with randomly assigned velocities and repeated four times, resulting in a total of 64 simulations. All the RAMD simulations were executed using the GPU version of GROMACS 2020.5.

### 4.6 Simulation Trajectory Analysis

Analyses of the RMSF, the free energy distribution for each replica, the free‐energy landscape of the RMSD versus the R_g_, and the residue distance statistics were performed using the built‐in modules of GROMACS. Moreover, clustering analysis was performed using the built‐in clustering module in GROMACS with the GROMOS method, employing an RMSD cutoff of 0.25 nm. The binding free energy of the enzyme–ligand complex was calculated through the gmx_MMPBSA tool [53]. Pearson correlation analysis between residue distances and R_g_ was conducted via CONAN [29]. Noncovalent interactions within the enzyme–ligand complex were assessed using PyContact [31], wherein contact frequency was defined as the ratio of frames exhibiting contact formation to the total number of frames. Collision event was defined when the distance between any atom of the ligand and any atom of a residue in the enzyme was less than 3 Å, following the methodology established by Cheng et al. [54], with determinations conducted using the Python package MDAnalysis [55]. The collision frequency was calculated as the ratio of frames containing collision events to the total number of frames. Protein structural visualization was carried out with PyMOL (https://github.com/schrodinger/pymol‐open‐source) or ChimeraX [56].

### 4.7 Genes, Plasmids, and Strains

The sequence of the wild‐type *ido* gene was extracted from *Bacillus thuringiensis* strain 2e2 AKU 0251 (GenBank: HM358019.1). The expression vector and strain used for this study were pET‐28a(+) and *E. coli* BL21(DE3), respectively. The recombinant strain *E. coli* BL21/pET28a‐IDO was constructed in a previous work [15] and preserved in our laboratory.

### 4.8 Genetic Constructions

Site‐directed mutagenesis of the “gate buckle” and substrate‐anchoring residues was carried out by whole‐plasmid PCR according to the following procedure. First, the template plasmid was extracted from cultured *E. coli* BL21/pET28a‐IDO. The wild‐type IDO recombinant strain, obtained from plate cultivation, was inoculated into 5 mL of kanamycin‐containing medium (final concentration of 50 mg/L) in tubes, followed by cultivation at 37°C and 200 rpm for 12 h. Bacterial cells were then collected by centrifugation at 12 000 rpm for 1 min (Centrifuge 5420, Eppendorf, Germany), after which the pET‐28a‐IDO plasmid was extracted as a mutagenesis template using the FastPure Plasmid Mini Kit (Vazyme Biotech Co., Ltd., China) according to the manufacturer's instructions. Subsequently, whole‐plasmid PCR was carried out using PrimeSTAR Max DNA Polymerase (TaKaRa Bio, Japan) with primers targeting the “gate buckle” residues (Table S7) or the substrate anchoring residues (Table S8). Finally, the PCR product was treated with QuickCut *Dpn* I (TaKaRa Bio, Japan) at 37°C for 1 h to digest the plasmid template, and the reaction mixture was transformed into *E. coli* BL21(DE3) competent cells by heat shock methodology [57].

A combinatorial mutation library targeting tunnel residues was designed with gene fragments synthesized by Twist Bioscience (Table S4). These mutant gene fragments and the pET‐28a(+) plasmid were separately double digested at 37°C for 20 min with the restriction endonucleases QuickCut *Sal* I (TaKaRa Bio, Japan) and QuickCut *Xba* I (TaKaRa Bio, Japan). Following digestion, both the gene fragments and the plasmid were separated by agarose gel electrophoresis and subsequently recovered using the FastPure Gel DNA Extraction Mini Kit (Vazyme Biotech Co., Ltd., China). The purified gene fragments and template were then ligated using T4 DNA Ligase (TaKaRa Bio, Japan) at 16°C for 30 min to facilitate recombination. Finally, the recombinant plasmids were transformed into *E. coli* BL21(DE3) competent cells by heat shock.

### 4.9 Protein Expression and Purification

After overnight culture of the recombinant strain on LB plate, a single colony was selected and transferred to 5 mL of LB medium supplemented with kanamycin (final concentration 50 mg/L) and cultivated at 37°C and 200 rpm for 6‒8 h. Subsequently, the culture was transferred to 30 mL of LB medium in a conical flask supplemented with kanamycin (final concentration 50 mg/L) and cultivated at 37°C and 200 rpm until the optical density measured at a wavelength of 600 nm (OD_600_) reached 0.6‒0.8. At this point, 1‰ (v/v) IPTG (isopropyl β‐D‐1‐thiogalactopyranoside) was added to induce overexpression of the target protein, and cultivation was continued at 200 rpm and 20°C for 12‒16 h. After induction, the bacterial suspension was centrifuged at 8,000 rpm for 10 min at 4°C (Centrifuge 5804 R, Eppendorf, Germany). The collected bacterial pellet was subsequently resuspended in protein purification buffer A (20 mM Tris‐HCl, 150 mm NaCl, pH 7.5). The bacterial cells were disrupted using an ultrasonic homogenizer (JY92‐IIN, Scientz Biotechnology Co., Ltd., China) under the following conditions: 35% power, 2 sec on/3 sec off cycles, for a total duration of 10 min. During cell disruption, the suspension was maintained in an ice‒water bath. The disrupted bacterial suspension was centrifuged at 10 000 rpm for 30 min at 4°C (Centrifuge 5804 R, Eppendorf, Germany) to remove insoluble cellular debris. Finally, the collected supernatant was filtered through a 0.45 µm microporous membrane for subsequent purification procedures.

Protein affinity purification was performed using an ÄKTA avant chromatography system (Cytiva, USA) with a 1 mL HisTrap HP nickel affinity column (Cytiva, USA). First, the column was equilibrated with protein purification buffer A equivalent to 10 column volumes. The previously collected supernatant was subsequently loaded onto the pre‐equilibrated column to facilitate binding of the target protein to the chromatography matrix. Following sample loading, the column was washed with 6% protein purification buffer B (20 mm Tris‐HCl, 150 mm NaCl, 500 mm imidazole, pH 7.5) equivalent to 15 column volumes to remove nonspecifically bound proteins. The target protein was then eluted from the column using 56% protein purification buffer B, with fractions collected in 2.5 mL volume units. Buffer exchange to eliminate high concentrations of imidazole from the affinity chromatography fractions was accomplished using PD‐10 desalting columns (Cytiva, USA). The desalting column was first equilibrated with low‐salt buffer (10 mm Tris‐HCl, 100 mm NaCl, 5 mm dithiothreitol, pH 7.5) equivalent to 5 column volumes. Subsequently, 2.5 mL of the affinity chromatography fraction was loaded onto the pre‐equilibrated desalting column. Following the sample application, the target protein was collected in 3.5 mL of low‐salt buffer. The final desalted target protein was verified by SDS‒PAGE, and its concentration was determined by a NanoDrop 8000 (Thermo Fisher Scientific, USA).

### 4.10 Product Detection and Characterization

The precolumn derivatization of samples was performed using Fmoc chloride (Fmoc‐Cl) for HPLC analysis. Specifically, following centrifugation of the enzymatic reaction mixture at 8000 rpm, 250 µL of the supernatant was transferred to a 2 mL tube. An equivalent volume of 20 mm borate buffer (pH 9.2) was added, followed by the addition of 500 µL of a 10 mm Fmoc‐Cl solution. The mixture was homogenized by pipetting and incubated in a thermo shaker incubator (MTC‐100, MIULAB, China) at 25°C and 800 rpm for 10 min. The reaction was quenched by the addition of 500 µL of 40 mm L‐adamantane, and the resulting mixture was filtered through a 0.22 µm organic membrane (BKMAM, China) into a brown vial. Chromatographic analysis was conducted using an E2695 system (Waters, USA) equipped with a Hypersil ODS‐2 column (250 × 4.6 mm, 5 µm; Thermo Fisher Scientific, USA). The measurement wavelength was set to 263 nm, and mobile phases A [50 mm NaAc‐HAc buffer (pH 4.2)/acetonitrile = 90:10 (v/v)] and B (acetonitrile) were used to separate the different components of the reaction mixture using gradient elution at a flow rate of 1 mL/min.

LC‒MS analysis was carried out using an ACQUITY UPLC‒MS/MS system (Waters, USA) equipped with an ACQUITY UPLC HSS C18 reverse‐phase column (1.8 µm, Waters, USA). Separation was achieved via gradient elution with mobile phases A [20 mm NH_4_Ac‐HAc buffer (pH 4.2)] and B (acetonitrile). The flow rate was maintained at 200 µL/min, the column temperature was stabilized at 25°C, and an injection volume of 5 µL was utilized.

### 4.11 Enzyme Activity Assays and Kinetic Measurements

Enzyme activity assays were performed in a 500 µL reaction system comprising 10 mm substrate, 10 mm αKG, 10 mm L‐ascorbic acid, 1.5 mm FeSO_4_·7H_2_O, and 50 mm Tris‐HCl buffer (pH 7.0), with a final enzyme concentration of 0.1 mg/mL. Following thorough mixing, the reaction mixture was incubated in the thermo shaker incubator (MTC‐100, MIULAB) at 30°C and 800 rpm for 10 min. Upon completion of the reaction, the tubes were immediately placed in a boiling water bath to inactivate the enzyme, after which the amount of product generated was measured by HPLC as described above. One enzyme activity unit (U) was defined as the amount of enzyme required to generate 1 µm product in 1 min at 30°C.

To measure kinetic parameters, the enzyme activity assay procedure was adopted, except that the enzyme concentration was adjusted to 25 mg/L and the substrate concentration gradient was set from 0.001 to 10 mm. The product and by‐product (succinic acid) are produced at equal stoichiometry in the IDO reaction (Figure S6). Therefore, succinate production was measured at different substrate concentrations to characterize enzyme activity following the procedure of the Succinate‐Glo JmjC Demethylase/Hydroxylase Assay Kit (Promega, USA). The kinetic parameters for each mutant were then derived by fitting the data to the Michaelis–Menten model using GraphPad Prism 9.0.0 software.

### 4.12 Screening of the Combinatorial Mutation Library

The information of combinatorial mutation library (Tunnel‐Lib) targeting tunnel‐associated residues in loop 2 was detailed in Table S4, with the corresponding DNA sequences were synthesized by Twist Bioscience. Then, the recombinant bacteria harboring the combinatorial mutation were constructed via double enzyme digestion following the workflow shown in Figure S9.

Recombinant bacterial colonies were inoculated into a 48‐deep‐well plate containing 1.5 mL of LB medium supplemented with kanamycin at a final concentration of 50 mg/L and cultivated at 37°C with shaking at 200 rpm for 8 h. Then, IPTG was added at 1‰ (v/v) to induce overexpression of the mutated protein for 16 h. Subsequently, the cell culture was centrifuged at 4°C and 5000 rpm for 10 min (Centrifuge 5804 R, Eppendorf, Germany) to pellet the cells, which were then resuspended in 20 mm Tris‐HCl buffer (pH 7.5). The resuspended cells were lysed by incubation with 6 units of DNase I (TaKaRa Bio, Japan) and 1 mg/mL lysozyme (Vazyme Biotech Co., Ltd., China) for 1 h at 30°C with shaking. Next, after the lysate was centrifuged at 4°C and 5 000 rpm for 30 min (Centrifuge 5804 R, Eppendorf, Germany), 100 µL of clarified supernatant was taken for the reaction in which an equimolar mixture of 5 substrates was used as the reaction substrate for high‐throughput screening. Finally, enzyme activity was assayed using the Succinate‐Glo JmjC Demethylase/Hydroxylase Assay Kit (Promega, USA). To ensure maximal coverage of the theoretical 1,728 variants in the Tunnel‐Lib library (Table S4), high‐throughput screening of 5000 mutant strains—approximately threefold the theoretical number—was performed.

## Author Contributions

L.W., H.L., and J.G. constructed mutants and cultivated expression hosts; Z.D. also performed organism cultivation. L.W., H.L., S.Z., L.Q., J.L., and Z.D. purified the protein, performed biochemical assays, high‐throughput screening, and activity characterization. L.W. and J.W. performed molecular dynamics simulations. L.W., H.L., and J.L. conducted structural bioinformatic analyses. L.W., Y.X., and Y.N. conceptualized and designed the study. L.W., H.L., F.L., and Y.N. wrote and revised the manuscript with contributions and final approval of all coauthors. Y.N. supervised the project and secured funding.

## Conflicts of Interest

The authors declare no conflicts of interest.

## Supporting information




**Supporting File 1**: advs75853‐sup‐0001‐SuppMat.pdf.

**Supporting File 2**: advs75853‐sup‐0002‐MovieS1.avi.

## Data Availability

The data that support the findings of this study are available from the corresponding author upon reasonable request.
