# Supplementary material for: Protein Structuromics Reveals a Loop‐Controlled Half‐Open Active Pocket Conformation Throughout Fe(II)/α‐ketoglutarate‐Dependent Dioxygenase Catalytic Cycle
Source: Adv Sci (Weinh). 2026 May 26:e75853. Online ahead of print. doi: 10.1002/advs.75853 (PMC13336071; doi:10.1002/advs.75853)
Supplement: Supplementary file 1 — Supporting File 1: advs75853‐sup‐0001‐SuppMat.pdf. [file ADVS-9999-e75853-s002.pdf]

Supporting Information

Protein structuromics reveals a loop-controlled half-open active pocket conformation throughout Fe(II)/α-ketoglutarate-dependent dioxygenase catalytic cycle

*Lunjie Wu, Huan Liu, Songyin Zhao, Lei Qin, Jiarui Li, Jun Wang, Zixuan Dai, Jie Gu, Yan Xu, Feiran Li, Yao Nie**

L. Wu, H. Liu, S. Zhao, J. Li, J. Wang, Z. Dai, J. Gu, Y. Xu, Y. Nie
Laboratory of Brewing Microbiology and Applied Enzymology, School of Biotechnology and Key Laboratory of Industrial Biotechnology, Ministry of Education, Jiangnan University, Wuxi 214122, China
E-mail: ynie@jiangnan.edu.cn (Y. Nie); Tel.: +86-510-85197760; Fax: +86-510-85918201

L. Qin
Key Laboratory of Biocatalysis & Chiral Drug Synthesis of Guizhou Province, Generic Drug Research Center of Guizhou Province, Green Pharmaceuticals Engineering Research Center of Guizhou Province, School of Pharmacy, Zunyi Medical University, Zunyi 563000, China

L. Wu, F. Li
Institute of Biopharmaceutical and Health Engineering, Tsinghua Shenzhen International Graduate School, Tsinghua University, Shenzhen 518055, China

Y. Nie
Key Laboratory of Industrial Synthetic Biology of Jiangsu Province, Jiangnan University, Wuxi 214122, China

Content

Supplementary Figures 1

Figure S1. Residues missing in loops within active pocket of IDO homologs in the PDB database. 1

Figure S2. Solvent accessible surface of IDO in closed and open conformations. 2

Figure S3. A comparative analysis of the free-energy landscapes of IDO as derived from classical MD and vsREMD simulations. 3

Figure S4. vsREMD simulation profile of IDO. 4

Figure S5. SDS-PAGE analysis of purified saturation mutants on “gate buckle” residues. 5

Figure S6. Reactions catalyzed by IDO. 6

Figure S7. Binding modes of IDO with different substrates. 7

Figure S8. SDS-PAGE analysis of purified dominant mutants on anchored residues. 8

Figure S9. Ligand and receptor profiles in docked complexes. 9

Figure S10. High-throughput screening procedure against tunnel residues mutation library. 10

Supplementary Table 11

Table S1. Data sets for αKGDs structuromics analysis. 11

Table S2. Hydrophobicity values for 20 amino acids. 24

Table S3. Kinetic parameters of wild-type IDO and dominant mutants on anchoring residues. 25

Table S4. Tunnel residues mutation library (Tunnel-Lib) design. 26

Table S5. Kinetic parameters of wild-type IDO and dominant mutants on tunnel residues. 27

Table S6. Temperature distribution for the vsREMD simulation. 28

Table S7. Primers for single-point saturation mutagenesis at the Y100 and W168 sites. 29

Table S8. Primers for iterative mutation of anchoring residues. 31

Supplementary Movie 33

Movie S1. Conformational change of loop2 from open to closed. 33

Supplementary References 34

Supplementary Figures

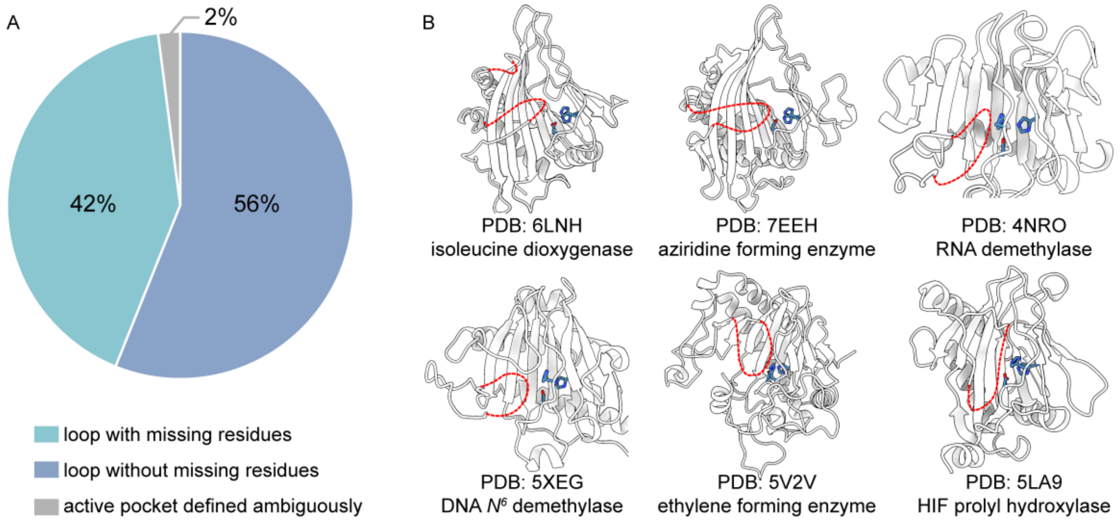

Figure S1. Residues missing in loops within active pocket of IDO homologs in the PDB database.

(A) Statistics of missing residues in loops within active pocket. (B) Structural examples of missing residues in loops within the active pocket. Red dashed lines indicate missing residues.

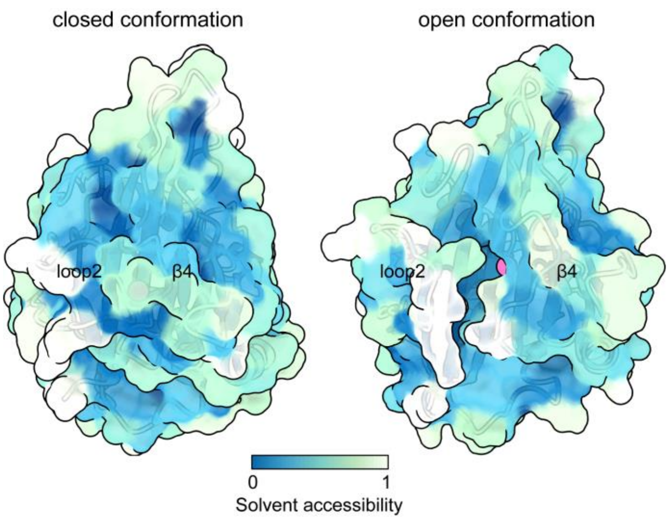

Figure S2. Solvent accessible surface of IDO in closed and open conformations.

The closed and open conformations of IDO were extracted from its MD trajectories. Solvent accessibility values were calculated by GETAREA[1] with default parameters and normalized, wherein high values indicate easier access. Loop2 and β4 show a clear cleft in the open conformation. The ferrous ion is represented by the pink spheres in the structure, and ferrous ion is more accessible in the open conformation compared to that in the closed conformation.

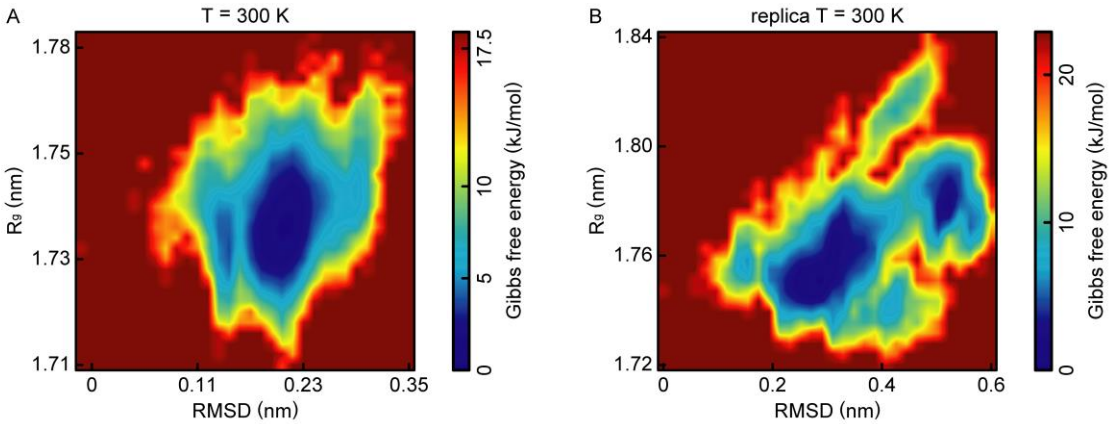

Figure S3. A comparative analysis of the free-energy landscapes of IDO as derived from classical MD and vsREMD simulations.

(A) The free-energy landscape of IDO extracted from classical MD simulations. The temperature was set to 300 K throughout the simulation. (B) The free-energy landscape of IDO extracted from vsREMD simulations. The temperature settings for each replica are detailed in Table S6. Here, the free energy landscape was extracted only from the simulation trajectories corresponding to the replica at T = 300 K.

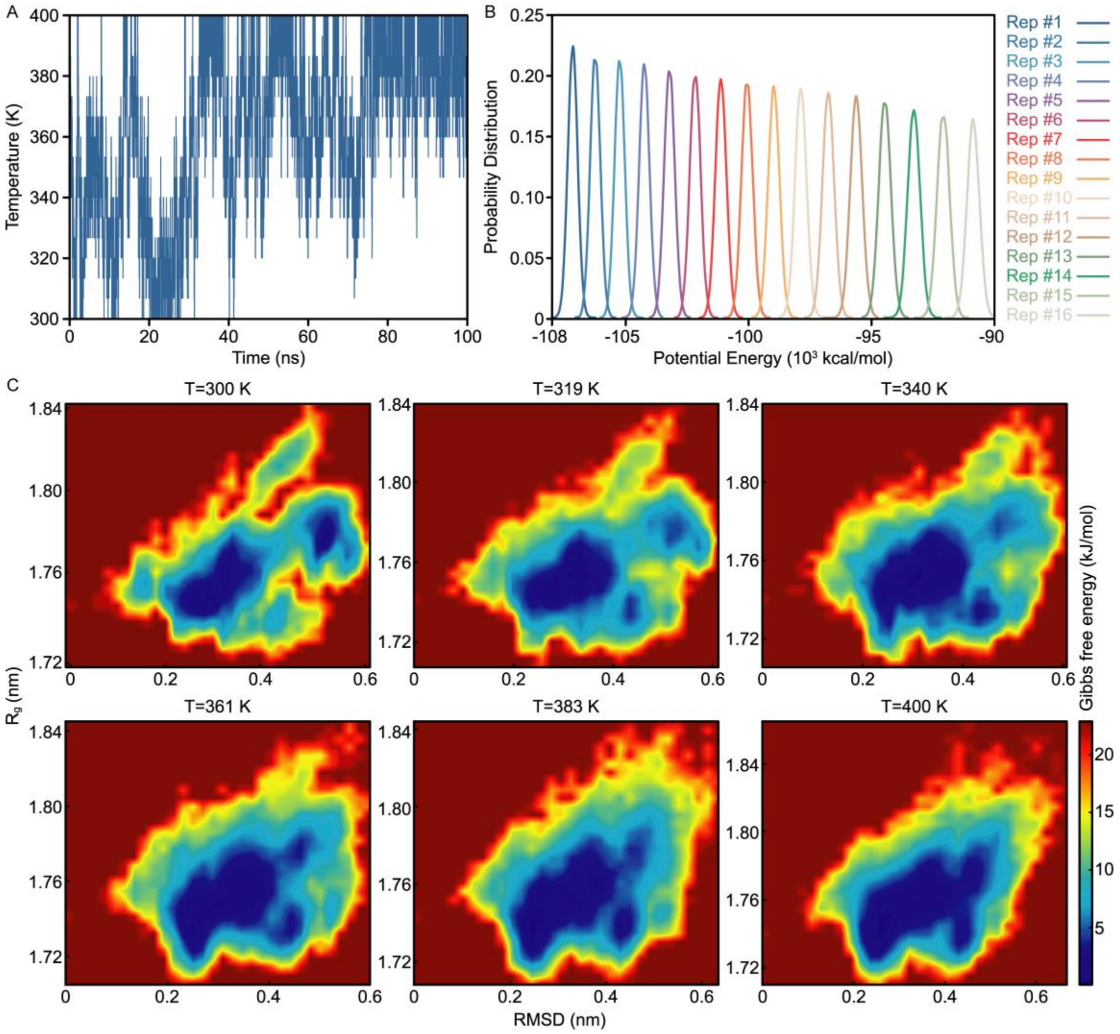

Figure S4. vsREMD simulation profile of IDO.

(A) Temperature history of an example replica in the vsREMD simulation of IDO. The replica of vsREMD traverses the set temperature range. (B) Distributions of potential energy of each replica. The probability distributions of potential energy observed in each replica follows a normal distribution, with significant overlap between the probability distributions of adjacent replicas. (C) Free energy landscape of IDO at selected temperature from vsREMD. The root mean square deviation (RMSD) has been widely employed as a standard metric for quantifying structural similarities between protein conformations[2]. A lower RMSD value indicates less conformational deviation from the reference structure. Meanwhile, the Radius of Gyration (R_g) serves as a physical parameter that characterizes the compactness of protein structures[3], whereby reduced R_g values typically correspond to more condensed protein conformations. Through the exchange of replica states, vsREMD simulations under high temperature conditions (e.g., 400 K) enable the surmounting of energy barriers, thereby comprehensively sampling the diverse conformational states of IDO.

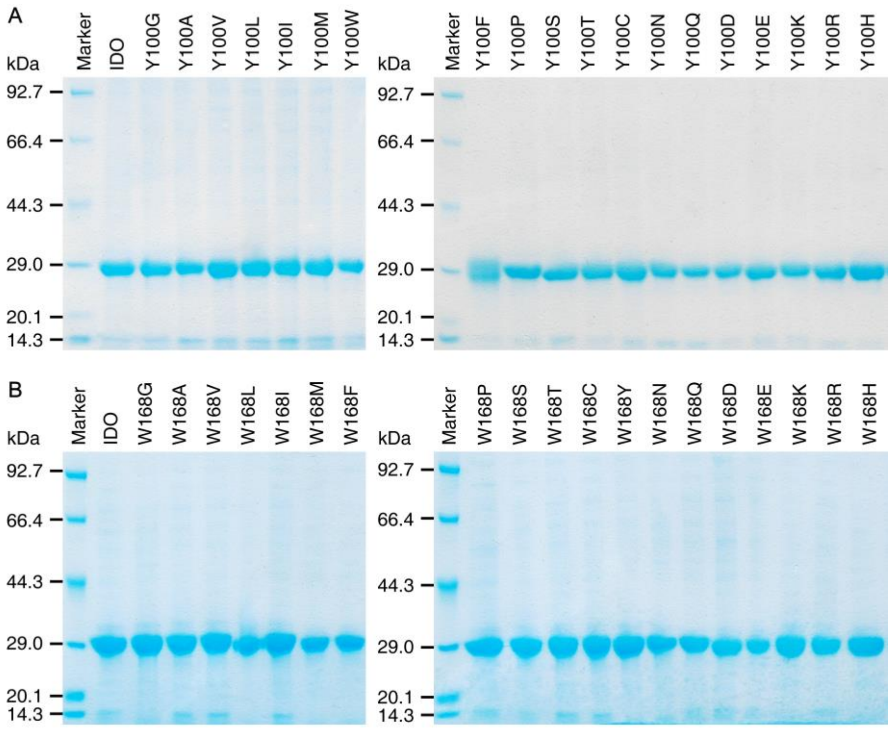

Figure S5. SDS-PAGE analysis of purified saturation mutants on "gate buckle" residues.

(A) SDS-PAGE analysis of purified saturated mutants on Y100 site. (B) SDS-PAGE analysis of purified saturated variants on W168 site. The theoretical molecular weights of IDO and its variants were calculated to be approximately 29.8 kDa.

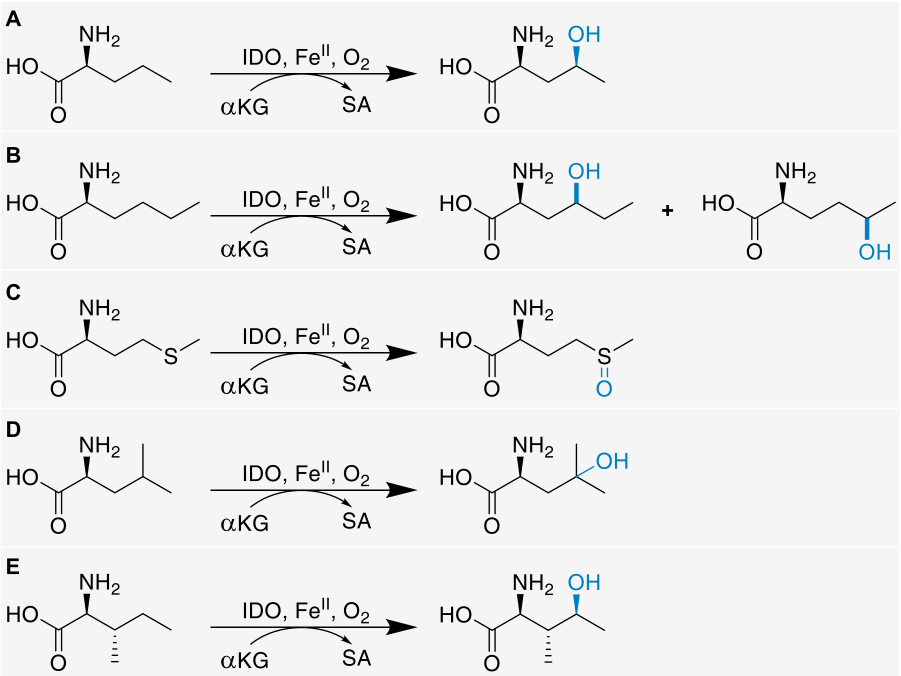

Figure S6. Reactions catalyzed by IDO.

C−H bond functionalization of L-Nva (A), L-Nle (B), L-Met (C), L-Leu (D), and L-Ile (E) catalyzed by IDO. αKG: α-ketoglutarate, SA: succinic acid. Products and by-products are produced in equal stoichiometric amounts in each reaction.

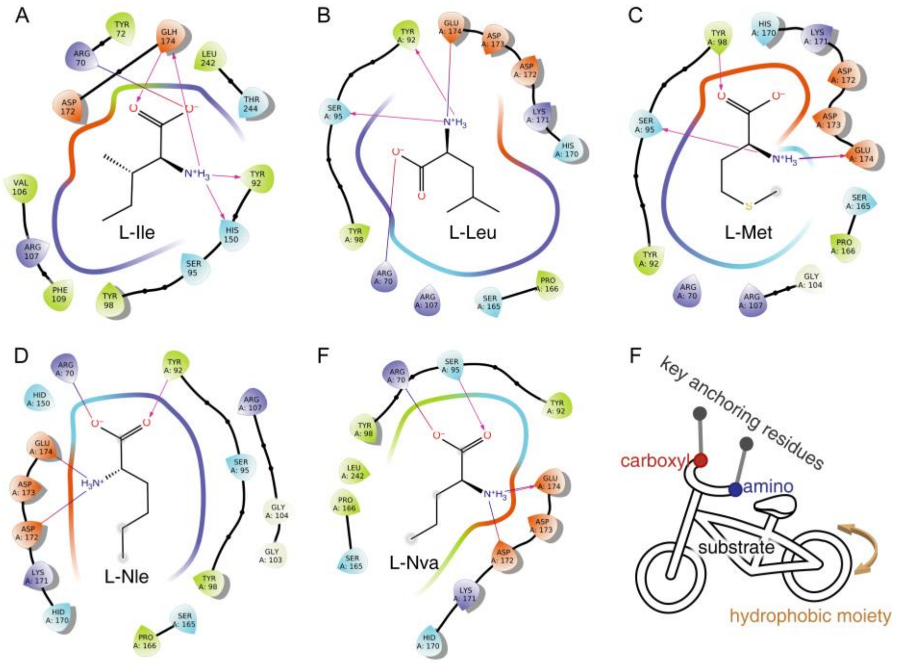

Figure S7. Binding modes of IDO with different substrates.

Bonding model of IDO with L-Ile (A), L-Leu (B), L-Met (C), L-Nle (D), and L-Nva (E). (F) The "handlebar" binding mode of IDO. The amino and carboxyl groups of the substrate are firmly anchored by key residues (such as R70 and E174), whereas its hydrophobic moiety features some degrees of conformational freedom that is typically regulated by van der Waals forces such as steric hindrance[4]. This binding mode is consistent with the "handlebar" binding mode of *cis*-P3H, another αKGDs member, that we previously reported[5].

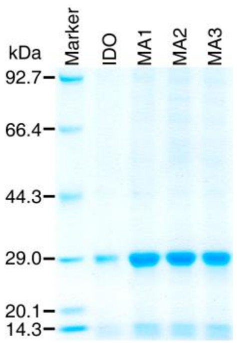

Figure S8. SDS-PAGE analysis of purified dominant mutants on anchored residues.

MA1: K96L/D102Q/K105I, MA2: D101Q/D102H/K105I, MA3: K96L/D102H/K105I. The theoretical molecular weights of IDO and its variants were calculated to be approximately 29.8 kDa.

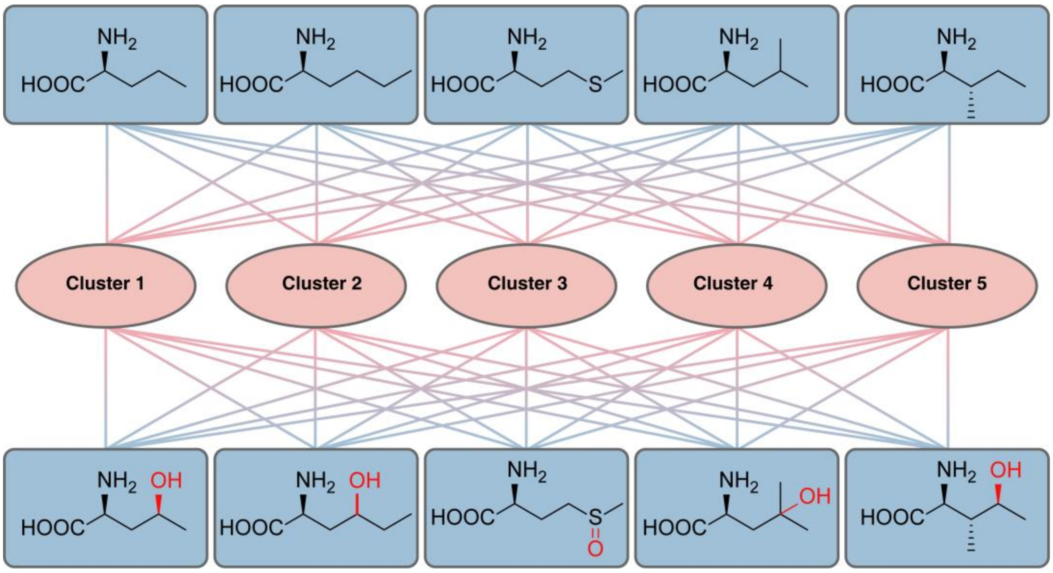

Figure S9. Ligand and receptor profiles in docked complexes.

Five representative conformations of IDO extracted from vsREMD simulations were selected as receptors (Fig. 4a), while five substrates and their corresponding products served as ligands. The docked 50 complexes were individually executed RAMD simulations.

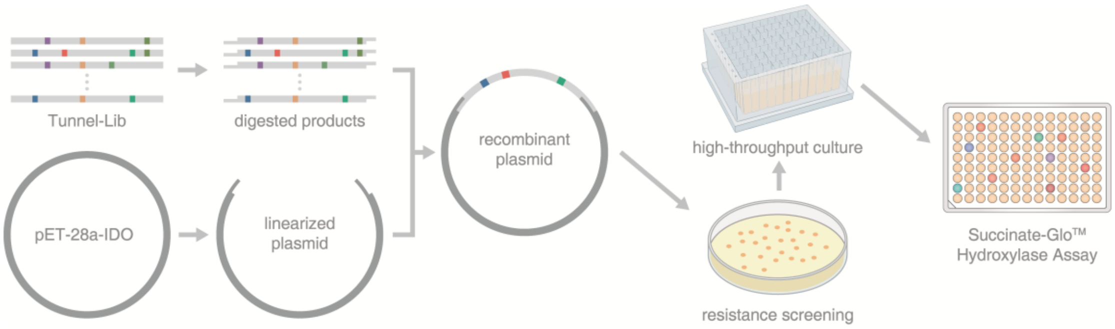

Figure S10. High-throughput screening procedure against tunnel residues mutation library.

Mutation library targeting tunnel residues were synthesized by Twist Bioscience.

Supplementary Table

Table S1. Data sets for αKGDs structuromics analysis.

Database	Protein ID
UniProt	A0A013XVH5, A0A014MD40, A0A014MUV5, A0A016XFB9, A0A017SJI2, A0A022PMX0, A0A022WD04, A0A022Y2R5, A0A023BU76, A0A023D7X1, A0A023WRE0, A0A024ED66, A0A024S2C9, A0A031FYV8, A0A031GY14, A0A031LG59, A0A031LW08, A0A059JH04, A0A059KIV2, A0A060NHZ7, A0A060NQF7, A0A060QCN9, A0A061ACP4, A0A066PSW8, A0A066RHC2, A0A066RQ75, A0A066U9W4, A0A066UI68, A0A066V171, A0A067B7Y0, A0A067Z3L6, A0A068L8C4, A0A068QP36, A0A068YXK4, A0A072NP64, A0A073K6T2, A0A075JVL9, A0A075LTJ7, A0A076PSN8, A0A077F8T9, A0A077LDK8, A0A077NE19, A0A077NKT2, A0A077NXM6, A0A077NXQ1, A0A077P373, A0A077P8M0, A0A077PLV3, A0A077PTB7, A0A077PVS9, A0A077PYZ3, A0A077Q9C6, A0A078BFV3, A0A078BL42, A0A081J7I9, A0A081KCW3, A0A081RHZ9, A0A084AT68, A0A084EL86, A0A084IG37, A0A084QNV9, A0A084SJ80, A0A084T440, A0A085F230, A0A085KAF6, A0A085PZ40, A0A085WB52, A0A086P5Q9, A0A086TGU8, A0A086WFJ3, A0A087I4T7, A0A087I726, A0A087NEB7, A0A089WMM9, A0A089YJE4, A0A090ID50, A0A090ILD5, A0A090KBF4, A0A090S2L8, A0A090T2J9, A0A091ALX7, A0A094B1M5, A0A094YF18, A0A096H5T7, A0A098BTZ1, A0A098UG23, A0A099CZ99, A0A099LLM7, A0A099W4W5, A0A099WRK0, A0A0A0DUN7, A0A0A0ELN3, A0A0A0EW23, A0A0A0N373, A0A0A0SK67, A0A0A0SUD1, A0A0A1I014, A0A0A1MN32, A0A0A1VL96, A0A0A2WEW3, A0A0A5HY70, A0A0A5I1J7, A0A0A5L6P2, A0A0A6FM51, A0A0A7EEQ3, A0A0A8F0L8, A0A0A8K3L4, A0A0A8XEZ3, A0A0B1YYA4, A0A0B5IBB6, A0A0B6S2H0, A0A0B6X6K3, A0A0B6XB10, A0A0B7D328, A0A0B7IWN7, A0A0B7JAN9, A0A0B7JN30, A0A0B7KIK5, A0A0B8P7Z9, A0A0B8P9M9, A0A0B8QLX4, A0A0C1NH27, A0A0C1QDZ4, A0A0C1ZLJ5, A0A0C2JN78, A0A0C2MDZ0, A0A0C2QLT4, A0A0C3DHR9, A0A0C3GTV6, A0A0C3IIJ5, A0A0C3IJW4, A0A0C5GB57, A0A0C5VEX8, A0A0C5XWU9, A0A0C9LGN4, A0A0D0L2B8, A0A0D0PD99, A0A0D5M1G8, A0A0D6E3T7, A0A0D6EB04, A0A0D6KR72, A0A0D6MHG6, A0A0D6MU23, A0A0D6N5N6, A0A0D6P4X9, A0A0D6Q3R0, A0A0D6Q652, A0A0D7CKK1, A0A0D7CQ51, A0A0D7K8Z3, A0A0D9MYA4, A0A0E1W0C4, A0A0E3BG99, A0A0E3BUD9, A0A0E3CJV8, A0A0E3V216, A0A0F0GN99, A0A0F0GPS5, A0A0F0IGQ7, A0A0F2HCP2, A0A0F2TA82, A0A0F3K1N9, A0A0F4INU9, A0A0F4NG75, A0A0F4NP33, A0A0F4PQ68, A0A0F4QN22, A0A0F4QQY0, A0A0F4XLQ9, A0A0F5ALM2, A0A0F5K1Y9, A0A0F5VFL7, A0A0F5ZXX7, A0A0F6A1C9, A0A0F6A6Q0, A0A0F7D3P5, A0A0F7HAI4, A0A0F7LT90, A0A0F9NEJ0, A0A0F9WHP2, A0A0F9ZXU9, A0A0G3BLL9, A0A0G3GDX7, A0A0G4I9E1, A0A0G4IG35, A0A0G4Q9J5, A0A0G9H507, A0A0G9HAC6, A0A0H0YI33, A0A0H1ANJ9, A0A0H1R5P2, A0A0H2LYU5, A0A0H2MRB5, A0A0H2MX09, A0A0H2WD51, A0A0H2Y0G5, A0A0H3AG97, A0A0H3CAW2, A0A0H3DBN0, A0A0H3E7Y2, A0A0H3HUM6, A0A0H3L116, A0A0H3NYK8, A0A0H4A2B8, A0A0H4BZV8, A0A0H4WIH5, A0A0H4X6F9, A0A0H4X9X6, A0A0H5GLD5, A0A0H5MC47, A0A0J0UKW4, A0A0J1DI52, A0A0J6H370, A0A0J7Y299, A0A0J8FRR8, A0A0J8SPS6, A0A0J8VSL9, A0A0J9D3E4, A0A0K0XUY0, A0A0K1JUY5, A0A0K1K3V0, A0A0K1PRZ8, A0A0K6I526, A0A0K6I9T2, A0A0K6IQF7, A0A0K8NYZ7, A0A0K8PK62, A0A0K8QPJ2, A0A0K9USN1, A0A0L0AGR9, A0A0L0EV75, A0A0L0HK97, A0A0L0NDJ4, A0A0L1JA31, A0A0L1MPW6, A0A0L6CM68, A0A0L6T442, A0A0L7N2V8, A0A0L7T4G3, A0A0L8KS13, A0A0L8LN16, A0A0L8LWB3, A0A0L8M219, A0A0M0EI04, A0A0M0HNG5, A0A0M0I1S3, A0A0M2DL94, A0A0M2SPU2, A0A0M2WJT6, A0A0M3AV05, A0A0M3QKF6, A0A0M4D751, A0A0M5J3M0, A0A0M8RIA3, A0A0M8SRM1, A0A0M8W6Y3, A0A0M8W703, A0A0M8XTR6, A0A0M9WNP3, A0A0N0CI92, A0A0N0GYH0, A0A0N0ME53, A0A0N0XK80, A0A0N0YFC3, A0A0N1AA95, A0A0N1B485, A0A0N1EJH0, A0A0N1EN85, A0A0N1L8I1, A0A0N8HW02, A0A0N8Q5D0, A0A0N8SQY1, A0A0N9I1R9, A0A0N9WE68, A0A0N9WGL9, A0A0P0LP29, A0A0P0P1D3, A0A0P4R5Z5, A0A0P7BC35, A0A0P7DN46, A0A0P7DWQ0, A0A0P7KN44, A0A0P7WFR8, A0A0P7Z3J0, A0A0P8A324, A0A0P8ZTN4, A0A0P9JB36, A0A0P9JQF0, A0A0P9PJE2, A0A0P9TNI4, A0A0P9TS37, A0A0P9W4Z3, A0A0Q0CIC1, A0A0Q0FIQ5, A0A0Q0J6E5, A0A0Q2M9X3, A0A0Q4ESF4, A0A0Q4GT77, A0A0Q4J5Z0, A0A0Q4P205, A0A0Q4RW41, A0A0Q4YF78, A0A0Q5D1W5, A0A0Q5HA70, A0A0Q5MF94, A0A0Q5ZE00, A0A0Q6A6T7, A0A0Q6MM13, A0A0Q6N8T6, A0A0Q6S794, A0A0Q6TG43, A0A0Q6TMD7, A0A0Q6TQG8, A0A0Q6VA42, A0A0Q6VUL3, A0A0Q6W6S5, A0A0Q6WNI9, A0A0Q6X0V7, A0A0Q7A392, A0A0Q7AQ05, A0A0Q7B1A5, A0A0Q7CZ03, A0A0Q7DGJ8, A0A0Q7DJ92, A0A0Q7F085, A0A0Q7F471, A0A0Q7IMJ2, A0A0Q7L8F3, A0A0Q7RZE5, A0A0Q7T4F9, A0A0Q7VAY2, A0A0Q7WHV0, A0A0Q7XIN6, A0A0Q8CUR3,

Database	Protein ID
UniProt	A0A0Q8DCY0, A0A0Q8EL36, A0A0Q8F129, A0A0Q8FK93, A0A0Q8GFG2, A0A0Q8I6X2, A0A0Q8ISI2, A0A0Q8LYU8, A0A0Q8RF48, A0A0Q8RF77, A0A0Q8SFA2, A0A0Q8SH53,
	A0A0Q8U4N7, A0A0Q9DIV6, A0A0Q9ETF3, A0A0Q9PDK6, A0A0Q9QM31, A0A0Q9XKB6, A0A0R0CT20, A0A0R0MA47, A0A0R3AI30, A0A0S2DAT2, A0A0S2FGS0, A0A0S2K577,
	A0A0S3F4N7, A0A0S3TSZ3, A0A0S4HU25, A0A0S4I5S8, A0A0S6XFE3, A0A0S9F4L4, A0A0S9KP31, A0A0S9MT62, A0A0T1Q5J9, A0A0T1SQB9, A0A0T2YQW7, A0A0T7BM46,
	A0A0T9LDS6, A0A0T9LHC1, A0A0T9NMW3, A0A0T9NWJ5, A0A0T9P9P8, A0A0T9T0W8, A0A0T9TX77, A0A0U0WGT3, A0A0U1DMW1, A0A0U2IRQ2, A0A0U2VW70,
	A0A0U3MEV1, A0A0U5B904, A0A0U5BFV3, A0A0W0HXU6, A0A0W0MZM7, A0A0W0SXY6, A0A0W1L3E9, A0A0W1LHR3, A0A0W7WX67, A0A0W7YVI8, A0A0W8JAD9,
	A0A0W8JGE8, A0A0X1KVZ0, A0A0X3V0W2, A0A0X3VRU7, A0A0X8GMS9, A0A0X8LMI7, A0A101LTU4, A0A103E7E5, A0A108U9J5, A0A109RVJ6, A0A117JN70, A0A120DH00,
	A0A120FQP2, A0A124PAA4, A0A126R726, A0A126RNC3, A0A126Z8Y4, A0A127JWP3, A0A127PEI6, A0A127QIC0, A0A128EKR7, A0A128EX36, A0A128F5H6, A0A128F812,
	A0A132CH55, A0A135I9A1, A0A135LAJ2, A0A137SP75, A0A139XB27, A0A140KA05, A0A143BYK3, A0A143G2W0, A0A147GX93, A0A149QH31, A0A149QR45, A0A149RVH9,
	A0A149SMP3, A0A149T107, A0A149TW62, A0A149UAG6, A0A149UJX1, A0A149UXJ1, A0A149V505, A0A149VC44, A0A150PN03, A0A150Q261, A0A150R0A4, A0A150R2Y2,
	A0A151GLW5, A0A151KXF3, A0A154QM29, A0A154R4Q5, A0A157QL66, A0A160A2J9, A0A160N4V7, A0A161Q7M1, A0A161VNA8, A0A162AT49, A0A162CY66, A0A164N198,
	A0A165IYG0, A0A166A4P2, A0A166HN65, A0A166UWB0, A0A167D0L3, A0A167DJS6, A0A167HBB6, A0A167KQU0, A0A167LAE9, A0A173KSH0, A0A176NMQ8, A0A177GDI0,
	A0A177JRA6, A0A177Q2Z5, A0A177XTT3, A0A177XXM0, A0A178AAP0, A0A178F7V7, A0A178FIW3, A0A178J5G9, A0A178JES1, A0A178K1L1, A0A179G3H7, A0A179HAN7,
	A0A181CDK8, A0A191UGL2, A0A191VA72, A0A193BUS2, A0A196MV68, A0A198FQM8, A0A198ULE8, A0A1A0CEY9, A0A1A0F9W8, A0A1A0JMR7, A0A1A0M041, A0A1A0MZL9,
	A0A1A0U960, A0A1A1WII7, A0A1A1YQK5, A0A1A2AY43, A0A1A2CJG0, A0A1A2E7I5, A0A1A2GE34, A0A1A2GIL4, A0A1A2JH70, A0A1A2M6Y3, A0A1A2M9S1, A0A1A2N1Q5,
	A0A1A2PEK1, A0A1A2PTL4, A0A1A2V0I9, A0A1A2WBF1, A0A1A2WDG0, A0A1A2X690, A0A1A2XQL6, A0A1A2YUE2, A0A1A2Z8E9, A0A1A2ZUV3, A0A1A3CDX0, A0A1A3CM82,
	A0A1A3EG26, A0A1A3FFH9, A0A1A3HWQ3, A0A1A3KFC7, A0A1A3ML95, A0A1A3NK50, A0A1A3QI96, A0A1A3SP81, A0A1A6B8J9, A0A1A6DVV5, A0A1A6M1U8, A0A1A7C671,
	A0A1A8TFP6, A0A1A8TH88, A0A1A9DHY6, A0A1A9HMF5, A0A1A9R7T2, A0A1A9T212, A0A1B1CLD5, A0A1B1KCF7, A0A1B3E099, A0A1B3LI32, A0A1B3M1Z8, A0A1B3MYW6,
	A0A1B3PMN9, A0A1B3W7S9, A0A1B3ZHH5, A0A1B4FW69, A0A1B4SJJ6, A0A1B5CVM9, A0A1B6AMF7, A0A1B6VLY7, A0A1B7U983, A0A1B7UWY3, A0A1B7V0F9, A0A1B7WLB8,
	A0A1B7WLV1, A0A1B8P3L3, A0A1B8QAG3, A0A1B8QBV9, A0A1B8YG73, A0A1B9P311, A0A1B9PLY6, A0A1B9PWV0, A0A1B9QBK4, A0A1B9R1U1, A0A1C0TLY1, A0A1C0U731,
	A0A1C0U7Q0, A0A1C0VNI2, A0A1C3EAL1, A0A1C3EBN1, A0A1C3EKV3, A0A1C3HIL2, A0A1C3J3I2, A0A1C3JIR4, A0A1C3JP08, A0A1C4IZZ3, A0A1C5AXA9, A0A1C5E854,
	A0A1C6M852, A0A1C6N998, A0A1C6PT11, A0A1C6Q4M5, A0A1C6VGZ7, A0A1C7F9L3, A0A1C7WD65, A0A1D2QN39, A0A1D2QQW5, A0A1D2S069, A0A1D2SBB0, A0A1D2SDV3,
	A0A1D2U7J2, A0A1D2ULQ8, A0A1D2XCY7, A0A1D4MHF8, A0A1D4NG63, A0A1D7VFZ8, A0A1D7VPL8, A0A1D7YH54, A0A1D7YK56, A0A1D8G9R4, A0A1D8QYF3, A0A1D8URG7,
	A0A1D9LF30, A0A1E1F0H8, A0A1E2WG86, A0A1E3GLR5, A0A1E3TIK2, A0A1E3VNY7, A0A1E3VPA3, A0A1E3WD75, A0A1E3WPA9, A0A1E3YU47, A0A1E4EI60, A0A1E4G0X2,
	A0A1E4H3I4, A0A1E4IT06, A0A1E4K9W9, A0A1E4MFM0, A0A1E4PZB0, A0A1E5A6G6, A0A1E5BAE4, A0A1E5BY19, A0A1E5C8K2, A0A1E5CXK1, A0A1E5E2X7, A0A1E5FUA5,
	A0A1E5P028, A0A1E5P2I6, A0A1E5P4Z4, A0A1E7EP99, A0A1E7K0U8, A0A1E7KWT7, A0A1E7LHE5, A0A1E7NGM2, A0A1E7WI15, A0A1E8PUQ2, A0A1F2U0Q8, A0A1F3AFG6,
	A0A1F4GAQ1, A0A1F4I3C8, A0A1F4MRN9, A0A1F5LY63, A0A1F7ZQ21, A0A1F8F309, A0A1F8VFT7, A0A1F8VUW4, A0A1G0CG38, A0A1G0I829, A0A1G0IIT8, A0A1G2KVX8,
	A0A1G3CT55, A0A1G3ILA9, A0A1G3JR32, A0A1G3ZF18, A0A1G4Q0H1, A0A1G4R647, A0A1G4Z536, A0A1G5J4C6, A0A1G5Q9R4, A0A1G5U4N1, A0A1G5X5M4, A0A1G6HZ92,
	A0A1G6IHY8, A0A1G6KRI5, A0A1G6PV41, A0A1G6SV10, A0A1G6UWM4, A0A1G6XHE9, A0A1G7L755, A0A1G7QCL7, A0A1G7R6Z1, A0A1G7USU5, A0A1G7XXV2, A0A1G7ZP14,
	A0A1G8AQW5, A0A1G8FMJ0, A0A1G8HHC5, A0A1G8IKB2, A0A1G8NS44, A0A1G8QVH2, A0A1G8R372, A0A1G8R9L2, A0A1G8T7K0, A0A1G8TUW2, A0A1G9A7A0,
	A0A1G9DKW9, A0A1G9IDA0, A0A1G9N352, A0A1G9P7I5, A0A1G9QVZ0, A0A1G9UY85, A0A1G9Y640, A0A1H0CQ97, A0A1H0EYW0, A0A1H0G0B6, A0A1H0ITM4, A0A1H0JXD9,
	A0A1H0M8R9, A0A1H0SGN9, A0A1H1ABP5, A0A1H1EI42, A0A1H1V661, A0A1H2BPR5, A0A1H2GEJ7, A0A1H2I8Z7, A0A1H2T3B4, A0A1H3DE33, A0A1H3H4L0, A0A1H3HEF3,

Database	Protein ID
UniProt	A0A1H3R0Q0, A0A1H3RUL7, A0A1H3V1S4, A0A1H3ZBW9, A0A1H4DH48, A0A1H4EII5, A0A1H4RL23, A0A1H4W1W5, A0A1H4YEX6, A0A1H4ZWT5, A0A1H5EBF5, A0A1H5EUK1, A0A1H5N735, A0A1H5YMC7, A0A1H6F3E9, A0A1H6VTL9, A0A1H6YNY5, A0A1H6ZH28, A0A1H7GVN7, A0A1H7HFN2, A0A1H7R6H3, A0A1H7R984, A0A1H7S592, A0A1H7SCU1, A0A1H7TB06, A0A1H7TIE0, A0A1H7VIB7, A0A1H8E376, A0A1H8MPB9, A0A1H8UB60, A0A1H8W138, A0A1H9EM32, A0A1H9F2I9, A0A1H9HXJ9, A0A1H9IY14, A0A1H9PRV7, A0A1H9SHR1, A0A1H9U000, A0A1H9WHJ8, A0A1H9WVF0, A0A1I0K3W4, A0A1I0MQT8, A0A1I0WPU3, A0A1I0XC41, A0A1I0YUC4, A0A1I1ECP8, A0A1I1ILV7, A0A1I1KEW4, A0A1I1LBE1, A0A1I1MPW8, A0A1I1U0J3, A0A1I2DHM8, A0A1I2F0D2, A0A1I2F897, A0A1I2GDU2, A0A1I2J992, A0A1I2PER7, A0A1I3EU63, A0A1I3ICQ6, A0A1I3NFF4, A0A1I3RP80, A0A1I3SKS9, A0A1I3VSS0, A0A1I3Y663, A0A1I4FZI3, A0A1I4I5I9, A0A1I4JUP5, A0A1I4Q3U9, A0A1I4RWT9, A0A1I5DRX7, A0A1I5HMK9, A0A1I5KDU0, A0A1I5N725, A0A1I5RCH5, A0A1I5TTB1, A0A1I6L856, A0A1I6LVL6, A0A1I7CRN9, A0A1I7F9I0, A0A1I7FM19, A0A1I7J1E5, A0A1I7LXE9, A0A1I9XTH4, A0A1J0VJ08, A0A1J5LDK5, A0A1J5P6A4, A0A1J5QZH8, A0A1J5RMZ7, A0A1J5RUL6, A0A1J5SHM2, A0A1J8P8R3, A0A1K1MVH6, A0A1K1Q6M5, A0A1K1RXV7, A0A1K2BMD0, A0A1K9ZFW1, A0A1L1PEV9, A0A1L1PTW6, A0A1L3ZP98, A0A1L5BMH3, A0A1L7AEK4, A0A1L9B9P5, A0A1L9GLP4, A0A1L9VWX9, A0A1L9VWY0, A0A1L9WQN7, A0A1M2V7M3, A0A1M2ZJG5, A0A1M3HNW7, A0A1M3N1Y3, A0A1M3NW10, A0A1M3PGU4, A0A1M4SU16, A0A1M4VRZ2, A0A1M5BMB3, A0A1M5JJU4, A0A1M5N1F3, A0A1M5ZF99, A0A1M6F418, A0A1M6U6F4, A0A1M7BQE0, A0A1M7JBW7, A0A1M7KHY9, A0A1M7R687, A0A1N6IQA8, A0A1N6M507, A0A1N6M6K6, A0A1N6M8M9, A0A1N6MWA9, A0A1N6N7F4, A0A1N6Q3C0, A0A1N6VAF9, A0A1N7AV75, A0A1N7E260, A0A1N7F171, A0A1N7J7Z9, A0A1N7LB52, A0A1P8FLP0, A0A1P8JVV6, A0A1P8KFF8, A0A1P8R4C7, A0A1Q2GXZ3, A0A1Q2LJ75, A0A1Q2YVF1, A0A1Q3HHZ7, A0A1Q3KFU3, A0A1Q3V6G1, A0A1Q3YGE4, A0A1Q4E3R2, A0A1Q4FQM3, A0A1Q4HFE7, A0A1Q4NXQ8, A0A1Q4WDI9, A0A1Q4ZD48, A0A1Q5I5S6, A0A1Q5IU70, A0A1Q5LCP2, A0A1Q5LR57, A0A1Q5TQP2, A0A1Q5TT97, A0A1Q5VIP2, A0A1Q7HJL8, A0A1Q7JDS3, A0A1Q8AUD0, A0A1Q8ILK5, A0A1Q8TC54, A0A1Q9AAK1, A0A1Q9G8S6, A0A1Q9HIZ6, A0A1Q9NB79, A0A1R0FQ43, A0A1R0KG34, A0A1R0TWJ0, A0A1R0VHB5, A0A1R1C9S0, A0A1R3F928, A0A1R3FBF6, A0A1R3FJ56, A0A1R4ECP8, A0A1R4GFT0, A0A1R4IV86, A0A1R4LJS6, A0A1S1MSV1, A0A1S1NAE1, A0A1S1UDP7, A0A1S1X656, A0A1S1XBN3, A0A1S2K1T6, A0A1S2KCW4, A0A1S2NCC2, A0A1S2NYZ1, A0A1S2PFM3, A0A1S2PJ57, A0A1S2Q3Q3, A0A1S2SVQ2, A0A1S7FYX5, A0A1S8D5V8, A0A1S8FKU1, A0A1S8GPL4, A0A1S9AC02, A0A1S9DPL5, A0A1S9E0N9, A0A1S9TM57, A0A1S9ZJ24, A0A1S9ZL53, A0A1T0ABI2, A0A1T0CKY0, A0A1T1IGZ0, A0A1T3CKE1, A0A1T4RI66, A0A1T4RLX8, A0A1T4UXL3, A0A1T4WIR4, A0A1T4ZVD6, A0A1T5AKJ0, A0A1T5I0E2, A0A1T5LDU4, A0A1U7H5C4, A0A1U7HAH2, A0A1U7IGC4, A0A1U9K3Z3, A0A1U9KEL2, A0A1U9KNL7, A0A1U9LI50, A0A1V0A054, A0A1V0QVE4, A0A1V0TKQ2, A0A1V2GUJ3, A0A1V2Q0G5, A0A1V2QRX7, A0A1V2RDG6, A0A1V3GQG7, A0A1V3H6P0, A0A1V3NYH8, A0A1V3PHP8, A0A1V3PIX1, A0A1V3PYV5, A0A1V3Q922, A0A1V3QQG6, A0A1V3S310, A0A1V3S5Z3, A0A1V3WKJ8, A0A1V3XXI5, A0A1V4CJH4, A0A1V4DTI1, A0A1V5G2H7, A0A1V6UG47, A0A1W1XFI9, A0A1W2B576, A0A1W5XNE4, A0A1W5XX35, A0A1W5YVT3, A0A1W6AHP4, A0A1W6K6S1, A0A1W6LC50, A0A1W6R581, A0A1W6W3A1, A0A1W6WIS9, A0A1W7M629, A0A1W9ZAU8, A0A1X0BUA5, A0A1X0IE74, A0A1X0JID1, A0A1X1MNB3, A0A1X1NAA1, A0A1X1NFC4, A0A1X1R8M8, A0A1X1RT09, A0A1X1UQY7, A0A1X1VD20, A0A1X1XYU3, A0A1X1ZHI4, A0A1X2ALB3, A0A1X2E334, A0A1X3D4S4, A0A1X7FJI7, A0A1X7FUA3, A0A1X7HI02, A0A1X7V5M9, A0A1Y0EST2, A0A1Y0G749, A0A1Y0N716, A0A1Y0RJ85, A0A1Y0UWU8, A0A1Y0XX49, A0A1Y1QDM4, A0A1Y1QL03, A0A1Y1QLH0, A0A1Y1SDM8, A0A1Y2NTJ2, A0A1Y2QJD0, A0A1Y2QPQ6, A0A1Y2R635, A0A1Y2SI40, A0A1Y2SQH6, A0A1Y3G189, A0A1Y3G5D6, A0A1Y5GWH4, A0A1Y5GY15, A0A1Y5HR79, A0A1Y5JYR0, A0A1Y6IP29, A0A1Y6JSR7, A0A1Y6KTW8, A0A1Y6M598, A0A1Y6M9N9, A0A1Y6MLP0, A0A1Z1SZF1, A0A1Z2KX62, A0A1Z2SAS7, A0A1Z4GPT3, A0A1Z4IC97, A0A1Z4JQR2, A0A1Z4KIA2, A0A1Z4MY84, A0A1Z4QPE4, A0A1Z4V1U5, A0A1Z4V2N2, A0A1Z5YUB0, A0A1Z8P1R8, A0A1Z9Z1T4, A0A202BA89, A0A208ZXP4, A0A210RW42, A0A210W6U7, A0A212T2U2, A0A212TV14, A0A212U3F8, A0A218Q7H3, A0A218QCI5, A0A218QQA2, A0A221KHH7, A0A222VJ74, A0A223MYC8, A0A223S0Q9, A0A227J285, A0A227JCG8, A0A228HMQ1, A0A229FXV5, A0A229REE8, A0A229S1V8, A0A233HD17, A0A235B471, A0A235EJL2, A0A235HUL5, A0A235IKN5, A0A235J6P4, A0A238D4M1, A0A238DM84, A0A238GZM0, A0A238Z8G2, A0A239DTU4, A0A239I313, A0A240CA26, A0A240CCC8, A0A240DY44, A0A240EK81, A0A240U3Q8, A0A240UDW3, A0A240UKV9, A0A240UTB7, A0A241NF82, A0A242V6S7, A0A243PDH9, A0A244CPD9, A0A244EFK7, A0A246JE06, A0A246PI45, A0A246RW41, A0A248VYP9, A0A249MV42,

**Database**	**Protein ID**
UniProt	A0A249W769, A0A250DPK8, A0A250I8S1, A0A250J4P8, A0A250K4L7, A0A250KYP7, A0A250VKW1, A0A252ARX2, A0A252B3C9, A0A252BKF9, A0A252BRJ9, A0A252BZZ0, A0A252C4D9, A0A252DGF9, A0A252E2Z5, A0A252EI63, A0A254NJV8, A0A254PSP8, A0A254PXS2, A0A255HZC6, A0A255PWT9, A0A255XJW8, A0A255ZNJ5, A0A257CB46, A0A257CLL3, A0A257DCC4, A0A257EF23, A0A257FZX9, A0A257H621, A0A257J0A8, A0A257LFP9, A0A257NVI9, A0A257P1U4, A0A257PJ98, A0A257PRF1, A0A257QCJ9, A0A257QD55, A0A257QXP8, A0A257R7E2, A0A257SCR2, A0A257UW58, A0A257WYK4, A0A258CW07, A0A258IP37, A0A258M9M7, A0A258MW73, A0A258PAQ1, A0A258QL71, A0A258S3P5, A0A258XBU7, A0A259BCX7, A0A259CY63, A0A259CZB1, A0A259DQJ0, A0A259FRU6, A0A259IAH1, A0A259INY5, A0A259ISI7, A0A259KQ11, A0A259MKU7, A0A259NBP2, A0A259NLE9, A0A259RNN3, A0A259S058, A0A261KJZ5, A0A261WPA8, A0A263NJY7, A0A263NTK7, A0A265DZI6, A0A268AEB3, A0A268BW36, A0A268HIB4, A0A269Y1M8, A0A270BFJ3, A0A271VUT8, A0A285N5C1, A0A285QC45, A0A285QUU7, A0A285U9B7, A0A285VXA7, A0A286B542, A0A286CF60, A0A286D6T5, A0A289GA42, A0A290N122, A0A290RWT4, A0A290SZV9, A0A290X189, A0A290XHY9, A0A291MYF4, A0A291N763, A0A291P846, A0A291PEN9, A0A291PKH3, A0A291QD02, A0A291RGU6, A0A292Z9E6, A0A2A2DB61, A0A2A2DCJ8, A0A2A2DN67, A0A2A2EPU6, A0A2A2NDC3, A0A2A2PKQ4, A0A2A2UVZ5, A0A2A3APM2, A0A2A3BN72, A0A2A3IKA8, A0A2A3IQN1, A0A2A3JKB3, A0A2A3LLI5, A0A2A3MXU8, A0A2A3VJM6, A0A2A4EQ81, A0A2A4G8C1, A0A2A4KK33, A0A2A4X3X9, A0A2A4ZZ11, A0A2A5D8Z6, A0A2A5DSL5, A0A2A5JQH6, A0A2A7T7K4, A0A2A7UWN6, A0A2A8HPY7, A0A2A9FEE2, A0A2A9FZT8, A0A2B2LV23, A0A2B7XUK6, A0A2B8BFR3, A0A2B9EE96, A0A2C5W8D2, A0A2C5Y911, A0A2C5YFF3, A0A2C6E3Y7, A0A2C6E7V4, A0A2C7AE99, A0A2C9PEQ0, A0A2D0HQT9, A0A2D0IFQ6, A0A2D0J4F6, A0A2D0JNI3, A0A2D0JRH0, A0A2D0K6M9, A0A2D0KI55, A0A2D0KTL7, A0A2D0L063, A0A2D2B429, A0A2D2DKC8, A0A2D2H356, A0A2D4VGU1, A0A2D5H908, A0A2D5T5V4, A0A2D5ZCS7, A0A2D6X2J1, A0A2D6X2U0, A0A2D6XC76, A0A2D7F8U4, A0A2D8MBY5, A0A2D8YCX0, A0A2D9TFL3, A0A2E1AHP4, A0A2E2JD10, A0A2E3EKH5, A0A2E3K8H3, A0A2E3PTW6, A0A2E4CH35, A0A2E4GWG4, A0A2E4QV56, A0A2E4S652, A0A2E4XXC6, A0A2E5CCU6, A0A2E5PIT8, A0A2E6QG68, A0A2E6W3P7, A0A2E7V2N3, A0A2E9AN19, A0A2E9C2B7, A0A2E9CU09, A0A2E9RW75, A0A2G0Q8Q8, A0A2G0QG31, A0A2G1LSF3, A0A2G1LUT4, A0A2G1XKW1, A0A2G2ABW7, A0A2G2IKQ3, A0A2G3J5F5, A0A2G3K350, A0A2G3LGG4, A0A2G4J9R2, A0A2G4KDW8, A0A2G4RDE3, A0A2G4U7T2, A0A2G5LM50, A0A2G5LW93, A0A2G5PDQ5, A0A2G5QWA6, A0A2G5RF67, A0A2G6DGG6, A0A2G6S8E4, A0A2G6SWK1, A0A2G6TU90, A0A2G6WUP9, A0A2G6Y5X1, A0A2G6ZPT9, A0A2G7G393, A0A2G7TC15, A0A2G8MVC3, A0A2G8RBV9, A0A2G8T074, A0A2G8TEU4, A0A2G9C762, A0A2G9L3B5, A0A2H1YJU1, A0A2H2ZS64, A0A2H3N3U5, A0A2H5DT43, A0A2H5DXF7, A0A2H6LJS1, A0A2I0FGK3, A0A2I0SYE5, A0A2I2M6V6, A0A2I3BZ72, A0A2I5HI46, A0A2I5T832, A0A2I5TAZ9, A0A2I8A5X4, A0A2J6UXE4, A0A2J7TJ31, A0A2J7V9D0, A0A2J7W8X1, A0A2J7YR40, A0A2J7ZI37, A0A2J7ZJZ3, A0A2J7ZW09, A0A2J8A3C2, A0A2J8ADE4, A0A2J8HU70, A0A2J9V2R1, A0A2K0SYM2, A0A2K0TNJ6, A0A2K0W8V3, A0A2K0Y3F4, A0A2K1Q2C4, A0A2K4FV25, A0A2K4IIJ2, A0A2K4MQU4, A0A2K4XD81, A0A2K8PV49, A0A2K8QIF9, A0A2K8SY12, A0A2K8UTF8, A0A2K9JSP8, A0A2K9K495, A0A2L0RUC1, A0A2L0WH35, A0A2L1WBC8, A0A2L2N1T8, A0A2L2NRF1, A0A2M6US31, A0A2M6VCU4, A0A2M6VI24, A0A2M6VLQ7, A0A2M6VRP3, A0A2M8K4U4, A0A2M8QIP6, A0A2M8QVA4, A0A2M8QVL4, A0A2M8VZL2, A0A2M8WPP5, A0A2M8X3S7, A0A2M8YE77, A0A2M9E0B3, A0A2M9EBD8, A0A2M9EKW9, A0A2M9EPR2, A0A2M9EU72, A0A2M9JNV0, A0A2N0HSM4, A0A2N0VXB7, A0A2N0WB29, A0A2N0XWG2, A0A2N0Y0C6, A0A2N1A4Z4, A0A2N1DUP9, A0A2N1H8N6, A0A2N2Q430, A0A2N2RPA3, A0A2N2SIC6, A0A2N2TP20, A0A2N2VKM7, A0A2N3CFU7, A0A2N3TF22, A0A2N4SS58, A0A2N4UTM2, A0A2N5CTF9, A0A2N5D863, A0A2N5E455, A0A2N5XAE6, A0A2N6IDV4, A0A2N6JUJ4, A0A2N6KBL2, A0A2N6LBQ6, A0A2N6MHE0, A0A2N7D0V5, A0A2N7EL25, A0A2N7F7W5, A0A2N7G9M1, A0A2N7GQ27, A0A2N7I6U3, A0A2N7IB70, A0A2N7J1X2, A0A2N7JCD2, A0A2N7JM48, A0A2N7KM19, A0A2N7L3I8, A0A2N7L463, A0A2N7MA03, A0A2N7N098, A0A2N7NPA6, A0A2N7QUR2, A0A2N7RD41, A0A2N7RHE9, A0A2N7TJ51, A0A2N7UFQ9, A0A2N7YCT5, A0A2N7YYV1, A0A2N8C5F7, A0A2N8DZE5, A0A2N8KUI5, A0A2N8MDP4, A0A2N8P164, A0A2N8Z932, A0A2N9B266, A0A2P1NKM1, A0A2P1PYU0, A0A2P2HE62, A0A2P4ZKC4, A0A2P6NXM9, A0A2P8KFQ2, A0A2P8L5V6, A0A2P8QJQ6, A0A2P8QNM2, A0A2R2IZB7, A0A2R3QFQ5, A0A2R4CFB3, A0A2R4TX06, A0A2R5FS35, A0A2R7L9Z3, A0A2R7N4N7, A0A2R7NRW8, A0A2R7SBS3, A0A2R8CH72, A0A2R8CLA5, A0A2S0MJX6, A0A2S0N5A3, A0A2S0Q5U2, A0A2S0VX37, A0A2S2DLJ8, A0A2S3R0X0, A0A2S3R816, A0A2S3VYQ6, A0A2S3X1P2, A0A2S3Y7F5, A0A2S4K7K7, A0A2S4KWH3, A0A2S4SZ15, A0A2S4X516, A0A2S4Z6I2, A0A2S5DFY4, A0A2S5DG43,

Database	Protein ID
UniProt	A0A2S5SYF5, A0A2S5T9B1, A0A2S5TFP1, A0A2S6AB74, A0A2S6B237, A0A2S6CVQ6, A0A2S6NMN6, A0A2S6TT79, A0A2S6UUL2, A0A2S6WCR3, A0A2S7J788, A0A2S7JJ13,
	A0A2S7JQT8, A0A2S7VDI0, A0A2S7VJG6, A0A2S7X2D2, A0A2S7X938, A0A2S7XKA5, A0A2S8H4N3, A0A2S8Q0D7, A0A2S8VN38, A0A2S8Z0W6, A0A2S9BGL2, A0A2T0JVL3,
	A0A2T0LQ57, A0A2T0MQG0, A0A2T0PPV1, A0A2T0QF20, A0A2T0RIY2, A0A2T0T7K2, A0A2T0VND8, A0A2T0XE82, A0A2T1C0M5, A0A2T1CUM5, A0A2T1E7F6, A0A2T1F5A0,
	A0A2T1F5C7, A0A2T2YR45, A0A2T2ZGN3, A0A2T3HSL8, A0A2T3HSN7, A0A2T3IM01, A0A2T3IPS9, A0A2T3JBV2, A0A2T3JP04, A0A2T3KAH3, A0A2T3LCE1, A0A2T3LJ59,
	A0A2T3MNF8, A0A2T3P2N3, A0A2T3QBF3, A0A2T3QEC7, A0A2T3YVM1, A0A2T4A445, A0A2T4AZS3, A0A2T4BU08, A0A2T4NP93, A0A2T4VS77, A0A2T5BQB3, A0A2T5ES82,
	A0A2T5LHC4, A0A2T5MCG7, A0A2T5R1V0, A0A2T6FB75, A0A2T6JEL8, A0A2T7AUG0, A0A2T7B171, A0A2T7K957, A0A2T7LXU7, A0A2T7SU05, A0A2T7T0H2, A0A2T7UE63,
	A0A2T9JIT2, A0A2T9JU49, A0A2T9K5N6, A0A2U0T3A2, A0A2U1DZF7, A0A2U1F089, A0A2U1RE36, A0A2U1RF87, A0A2U1V5H2, A0A2U1W161, A0A2U1WFH2, A0A2U1XSA9,
	A0A2U1YL87, A0A2U2I6W0, A0A2U3HDI6, A0A2U3N4E0, A0A2U3NAZ7, A0A2U8FVX3, A0A2U8I5S2, A0A2U8TKX8, A0A2U8VP89, A0A2U9TGF8, A0A2V0MX28, A0A2V2AC65,
	A0A2V2AT31, A0A2V2BE80, A0A2V2PVS3, A0A2V4NN02, A0A2V4R2Z3, A0A2V5HDL2, A0A2V5I9N0, A0A2V7ZRT0, A0A2V8B2G6, A0A2V8BNI2, A0A2V8CTL5, A0A2V8DP14,
	A0A2V8EHK6, A0A2V8EP10, A0A2V8FDM6, A0A2V8ZG54, A0A2V9T411, A0A2V9U1J9, A0A2V9U7M1, A0A2V9VPW1, A0A2V9WM52, A0A2W0F8T9, A0A2W1JG21, A0A2W2DAC0,
	A0A2W2I5C3, A0A2W4LYR8, A0A2W4MD31, A0A2W4VS56, A0A2W5DS54, A0A2W5PEV1, A0A2W5WWX7, A0A2W6TFJ7, A0A2W6XL26, A0A2W6YWL0, A0A2W7HXN1,
	A0A2W7ILI2, A0A2W7JME6, A0A2X2C7E5, A0A2X2D419, A0A2X2H4Z6, A0A2Z2H618, A0A2Z2H824, A0A2Z2LF58, A0A2Z3X9D0, A0A2Z4JR85, A0A2Z4PRL9, A0A2Z4V2Z7,
	A0A2Z4ZFL6, A0A2Z5UJI5, A0A2Z5YZ04, A0A2Z6ACX4, A0A2Z6CYV5, A0A2Z6D3R0, A0A315ATF6, A0A315AUS8, A0A315B5K5, A0A315BME3, A0A315C417, A0A315C8I6,
	A0A315CCY8, A0A315CEG6, A0A315CEN3, A0A315D6Q5, A0A315D7P7, A0A315DZ60, A0A315EFF1, A0A315ENQ9, A0A315EW83, A0A315F1S4, A0A315FFB9, A0A315TAT3,
	A0A316I6X0, A0A317FM42, A0A317ILA5, A0A317NC95, A0A317RFR2, A0A317WQ63, A0A318A5X9, A0A318B4R9, A0A318DQJ5, A0A318GYF2, A0A318JV07, A0A318JZJ3,
	A0A318LW36, A0A318PIM2, A0A318PR91, A0A318QET2, A0A318QP74, A0A318QYQ8, A0A318SEQ3, A0A318T2Q1, A0A318VAY0, A0A318VEP1, A0A318ZSY4, A0A319DNZ4,
	A0A327M6F7, A0A327N2K3, A0A327UE88, A0A327UIB2, A0A328M8I4, A0A328NC07, A0A328NWJ0, A0A328SWZ1, A0A328XYM6, A0A328YTM4, A0A329AFV5, A0A329E709,
	A0A329VN34, A0A329W251, A0A329W2P4, A0A329WHH8, A0A329XBG5, A0A330LUI0, A0A344J6S6, A0A344SCI9, A0A344U7Y7, A0A344UFL3, A0A345DBN6, A0A345RIT7,
	A0A345Y008, A0A345ZGK3, A0A346DR49, A0A346N3M6, A0A346R0D5, A0A346R3G9, A0A347WBG9, A0A349JH40, A0A349KYH3, A0A350CVQ0, A0A350XMD2, A0A350ZW68,
	A0A351L4Z1, A0A353HRP0, A0A353XUC7, A0A353Y572, A0A354IZA4, A0A354V9A8, A0A356TCC7, A0A357A6G4, A0A358BF61, A0A358SQ02, A0A363RPB5, A0A365YZA5,
	A0A365ZJ34, A0A366A526, A0A366AHC2, A0A366DMI5, A0A366JF39, A0A366W7X2, A0A366XGL3, A0A367EER2, A0A367F2L0, A0A367QF44, A0A367RD06, A0A367RRN9,
	A0A368KHS2, A0A368L7Y3, A0A368LMK5, A0A368NZ48, A0A368TWI1, A0A368XZV0, A0A369A550, A0A369T7T8, A0A369VMD8, A0A370FFW8, A0A370G955, A0A370HBY1,
	A0A370HXR7, A0A370K504, A0A370KDZ9, A0A370QNV3, A0A370SYY1, A0A370UBX1, A0A370WW86, A0A370X257, A0A370X9U1, A0A371JWZ1, A0A371PP98, A0A371Z0P6,
	A0A372DM62, A0A372EFD4, A0A372LYX9, A0A372M3W9, A0A372ZN04, A0A373FRU3, A0A377RS18, A0A378JI86, A0A378MIW3, A0A378PJR8, A0A378Q3K5, A0A378R5V6,
	A0A379F7I5, A0A379N383, A0A379U0K0, A0A379YPW3, A0A380A2L9, A0A380MT41, A0A380PXJ8, A0A380ZFM3, A0A381EY72, A0A382EDY9, A0A382KLU5, A0A386HB25,
	A0A395NJV6, A0A397HH63, A0A398B1N6, A0A398C1I8, A0A399GZP5, A0A399PV80, A0A3A1P4L1, A0A3A2I8F2, A0A3A2Z7L7, A0A3A3D335, A0A3A3EYV7, A0A3A3FGA8,
	A0A3A3G9W6, A0A3A4KNZ2, A0A3A6R037, A0A3A6TNP5, A0A3A8FD45, A0A3A8GN17, A0A3A8KR35, A0A3A8NCX7, A0A3A8PZN7, A0A3A9J1I2, A0A3A9JJ33, A0A3A9W7C6,
	A0A3A9X2K1, A0A3B0AT61, A0A3B0UUR4, A0A3B1AEV6, A0A3B7L227, A0A3B8JDA7, A0A3B9BBI2, A0A3B9BBI6, A0A3B9GZI3, A0A3B9IEF9, A0A3B9NXQ3, A0A3C0HLM0,
	A0A3C0KCF0, A0A3C0NFZ3, A0A3D0DW80, A0A3D0EV27, A0A3D1PB74, A0A3D1PCN0, A0A3D1Z3M1, A0A3D2HBF5, A0A3D2W268, A0A3D4ARJ9, A0A3D4TT12, A0A3D5VT53,
	A0A3D6AGJ8, A0A3D8I3J6, A0A3D8T4P5, A0A3D8VF24, A0A3D9DWU7, A0A3D9DY67, A0A3D9UGT3, A0A3E0DQ56, A0A3E0HKK4, A0A3E0M481, A0A3E0M4G0, A0A3E1RHW0,
	A0A3E2EBR6, A0A3E2H8J6, A0A3F3IXL3, A0A3G2EEL2, A0A3G2JLS5, A0A3G2PVD0, A0A3G3GIH6, A0A3G7Y8S1, A0A3G8C7T9, A0A3J6B5Q5, A0A3L0YF21, A0A3L9YNY1,

Database	Protein ID
UniProt	A0A3M2I0C4, A0A3M3EIL1, A0A3M3F728, A0A3M3RP38, A0A3M3Z945, A0A3M4DKV4, A0A3M4M5M2, A0A3M4QH99, A0A3M4W0P4, A0A3M4W6L7, A0A3M4YSV5, A0A3M5SHE7, A0A3M5UV07, A0A3M5VIQ0, A0A3M6A6C9, A0A3M6ACY7, A0A3M8K3R3, A0A3M8QCQ4, A0A3M8SNL9, A0A3M8TYW0, A0A3M9Z8V8, A0A3N0V1D7, A0A3N0V776, A0A3N1A245, A0A3N1PKC9, A0A3N1V8F0, A0A3N1WV41, A0A3N2DPG6, A0A3N2R010, A0A3N2RNQ8, A0A3N3DUN7, A0A3N4UIQ2, A0A3N4V1E1, A0A3N4YK64, A0A3N4ZHX0, A0A3N5GX03, A0A3N6PG98, A0A3N7BFB3, A0A3N7CCY8, A0A3N7CV90, A0A3N7DAI2, A0A3N7FPH8, A0A3N7JV57, A0A3N8GK46, A0A3N9RQ25, A0A3N9TGT3, A0A3N9UB70, A0A3N9UIR8, A0A3N9WQC7, A0A3N9WZ83, A0A3P3F407, A0A3Q0L0D6, A0A3Q0L6D1, A0A3Q8I3W5, A0A3Q8Z932, A0A3Q9AI25, A0A3Q9FVT6, A0A3Q9IFK2, A0A3R7IVM2, A0A3R7TYE0, A0A3R7V6K7, A0A3R8NW70, A0A3R8SBR9, A0A3R9BHY7, A0A3R9CJL1, A0A3R9NZ83, A0A3R9P837, A0A3R9VPX5, A0A3R9W7T4, A0A3R9WV81, A0A3R9XHI0, A0A3S0D549, A0A3S0MPX2, A0A3S0PG44, A0A3S0Y3R3, A0A3S0YLM6, A0A3S0ZTA2, A0A3S1C8W2, A0A3S1FEL8, A0A3S1FT15, A0A3S1KED7, A0A3S1M0V5, A0A3S1P8L0, A0A3S1T2M9, A0A3S1XSB3, A0A3S1Y8P6, A0A3S2BL84, A0A3S2BW55, A0A3S2TSW3, A0A3S3CZS0, A0A3S3G3P2, A0A3S3GXN4, A0A3S3RRW5, A0A3S4FUX2, A0A3S4GNI4, A0A3S4QT63, A0A3S5JH60, A0A3S8W4N1, A0A3S8YDE0, A0A3S8YIC6, A0A3S9PTJ2, A0A3S9Y5U1, A0A3T0JMM4, A0A3T0VZP1, A0A3V0PQ28, A0A401J3H1, A0A401KBJ2, A0A401M8N6, A0A401VZE1, A0A401WGF9, A0A401WWX7, A0A401X6J0, A0A401Z1S9, A0A402NRL6, A0A403FLK9, A0A403T324, A0A410UFK6, A0A410UPP2, A0A410XAH5, A0A411HF89, A0A411MQU5, A0A411WT92, A0A418Y6Q2, A0A418YT81, A0A419R4B6, A0A419R4C8, A0A419V4J0, A0A420E9Z5, A0A420EA77, A0A420ENZ4, A0A420ERV5, A0A420WZ00, A0A421D7G3, A0A422QGB7, A0A423GEI8, A0A423HF05, A0A423HWG4, A0A423IJR5, A0A423J2M9, A0A423K2E8, A0A423KST6, A0A423LUH1, A0A423MH24, A0A423N657, A0A423N704, A0A423NB21, A0A423NJH3, A0A423NQP2, A0A423PBG7, A0A423XRB7, A0A426VDN4, A0A426VS48, A0A427KBZ5, A0A427TZD8, A0A428XXU9, A0A429I0X9, A0A429J678, A0A429QFK5, A0A429TF37, A0A429TP01, A0A429TR01, A0A429URL1, A0A429WQL8, A0A430C6A2, A0A430GYK0, A0A430HEU6, A0A431JM65, A0A431K2C4, A0A431LPT1, A0A431TFW8, A0A431V7L9, A0A432F2H7, A0A432LYI4, A0A432RWS7, A0A433BQA6, A0A433MQC9, A0A433ULV2, A0A433WUB3, A0A434TNP4, A0A434WX35, A0A435A0X8, A0A436DK15, A0A436SAX2, A0A437LUF4, A0A437M6P9, A0A437MPC6, A0A437PEH5, A0A437RHA4, A0A440GMG8, A0A440IJI3, A0A440KE86, A0A441XRY6, A0A441Y986, A0A442I8L8, A0A442S2I3, A0A444KF22, A0A447QH91, A0A447R674, A0A448DJJ3, A0A448S742, A0A448SUW0, A0A454DCW3, A0A479ZXI0, A0A480AHY2, A0A480AX88, A0A481QQV0, A0A482Z8V1, A0A494UZF4, A0A494WAL5, A0A495FCW9, A0A495HXW7, A0A495T2B0, A0A495TK87, A0A497VHM8, A0A498E1W3, A0A498PPN9, A0A498QLJ5, A0A4D4JWG9, A0A4D4KRS9, A0A4D4KSQ4, A0A4D6XEN8, A0A4D7DGQ2, A0A4D7Z0D0, A0A4P5PBE5, A0A4P6FW15, A0A4P6H8C1, A0A4P6KZY9, A0A4P6QM36, A0A4P6UDV4, A0A4P6UNH2, A0A4P7F570, A0A4P7H5N7, A0A4P7PAJ3, A0A4P7R7D0, A0A4P7WC11, A0A4P7WFM8, A0A4P7YZJ9, A0A4P8HR11, A0A4Q0YNQ0, A0A4Q0YST5, A0A4Q2RH32, A0A4Q2UB84, A0A4Q2UDC8, A0A4Q3J7B6, A0A4Q3KVU8, A0A4Q3LJ96, A0A4Q3MY95, A0A4Q3NH34, A0A4Q4L692, A0A4Q4T3A5, A0A4Q4TJP6, A0A4Q4TQY9, A0A4Q4U388, A0A4Q5KNK8, A0A4Q5KRJ1, A0A4Q5KUP5, A0A4Q5PEX2, A0A4Q5PSK2, A0A4Q6BME9, A0A4Q6C3H2, A0A4Q6GS34, A0A4Q6JAW0, A0A4Q6XCP9, A0A4Q6Y9J9, A0A4Q7CRP8, A0A4Q7EJZ5, A0A4Q7ILT4, A0A4Q7J3C1, A0A4Q7JML5, A0A4Q7KK01, A0A4Q7LT18, A0A4Q7W037, A0A4Q8LI15, A0A4Q8LQI2, A0A4Q9G862, A0A4Q9GYS3, A0A4Q9KMK5, A0A4Q9MID1, A0A4Q9P7I1, A0A4R0Y8I1, A0A4R0YEA1, A0A4R0YXL2, A0A4R1EVY1, A0A4R1HPN9, A0A4R2B133, A0A4R2JNL6, A0A4R2JUY9, A0A4R2M6G8, A0A4R2MWX5, A0A4R2YM88, A0A4R3H2I7, A0A4R3HXX0, A0A4R3L8D2, A0A4R3LFC9, A0A4R3NDZ5, A0A4R3PQL3, A0A4R3VL63, A0A4R3XR09, A0A4R3YP04, A0A4R3ZY90, A0A4R4DA01, A0A4R4EXY0, A0A4R4JHA8, A0A4R4QMC1, A0A4R4Y0N7, A0A4R4Y7H2, A0A4R5HE76, A0A4R5K096, A0A4R5QBV1, A0A4R5TRC0, A0A4R5U6R3, A0A4R6HTX0, A0A4R6MB70, A0A4R6N3R7, A0A4R6QKB0, A0A4R6TJS2, A0A4R6TV40, A0A4R6WYR5, A0A4R6Y8S0, A0A4R6YN12, A0A4R6ZGA3, A0A4R6ZGP9, A0A4R7FMP2, A0A4R7GAC2, A0A4R7NFK5, A0A4R7SF27, A0A4R7V6N0, A0A4R7VCD7, A0A4R7VTC8, A0A4R8KVT6, A0A4R8LZ88, A0A4R9W734, A0A4S0G2D0, A0A4S0KBW8, A0A4S0N717, A0A4S1FDA8, A0A4S1M451, A0A4S2CU23, A0A4S2QXM0, A0A4S2RGV4, A0A4S2T9W8, A0A4S3FXD0, A0A4S3K2H3, A0A4S3KKN7, A0A4S3KLU7, A0A4S4AB98, A0A4S5EU55, A0A4U0GBE7, A0A4U0NRK2, A0A4U0NW68, A0A4U1ISJ1, A0A4U1WHM2, A0A4U1YQT1, A0A4U2A640, A0A4U2D2W7, A0A4U2EK94, A0A4U2EVE2, A0A4U2H235, A0A4U2MXL7, A0A4U3B9V0, A0A4U5JKQ4, A0A4U6GL86, A0A4U6PMQ7, A0A4U8SGQ7, A0A4U8UBH7, A0A4U8Z514, A0A4U9HNP5, A0A4U9U1D0, A0A4V1ABE8, A0A4V1BHR6,

Database	Protein ID
UniProt	A0A4V1G7F4, A0A4V1N0V2, A0A4V1TV60, A0A4V1UN65, A0A4V1WAQ1, A0A4V1XGE1, A0A4V2BJX8, A0A4V3CV98, A0A4V3FXM2, A0A4V5PJD7, A0A4V5TUX3, A0A4V5UIF9, A0A4V6IBV0, A0A4Y1MZ30, A0A4Y3M0L0, A0A4Y3QV83, A0A4Y3QXU8, A0A4Y3RDX9, A0A4Y3RTT5, A0A4Y3THX5, A0A4Y3TT87, A0A4Y4EXS4, A0A4Y5Z3G8, A0A4Y6RE80, A0A4Y6U9R4, A0A4Y6ULS2, A0A4Y6V2Q1, A0A4Y7ZN21, A0A4Y8WCL5, A0A4Y8WKM6, A0A4Y8Y7D7, A0A4Y9MHY6, A0A4Y9S878, A0A4Y9S930, A0A4Y9SSP7, A0A4Z0BFX1, A0A4Z0C1A8, A0A4Z0C3S2, A0A4Z1PK61, A0A4Z1R9I7, A0A502CHN5, A0A502E0D7, A0A502GHD4, A0A502HR76, A0A502VQB0, A0A506UME1, A0A507BHZ5, A0A507DWK2, A0A507FKE6, A0A508ARW0, A0A508AT44, A0A510I5Z2, A0A510UFC1, A0A510XGM8, A0A510XWU5, A0A510Y2K3, A0A511B3Z6, A0A511B586, A0A511BNC5, A0A511HFB9, A0A511MGS3, A0A511MSN1, A0A511SZB0, A0A511UST0, A0A511X9S1, A0A511XK79, A0A511ZNG6, A0A512DN64, A0A512DWX3, A0A512NHX1, A0A514BN79, A0A515CYQ0, A0A515DCY0, A0A515ER74, A0A516V7A4, A0A517L681, A0A517WAZ7, A0A518BN64, A0A518CV69, A0A518EZ93, A0A518N352, A0A519EGV9, A0A519EWA8, A0A519FNH7, A0A519GJG9, A0A519GWW4, A0A519H0W9, A0A519H3B4, A0A519IGW5, A0A519IN33, A0A519ITF4, A0A519J5F6, A0A519LX54, A0A519Z0Y1, A0A519ZGK0, A0A520DI12, A0A520DIU6, A0A520DZQ7, A0A520FUM7, A0A520GAV0, A0A520GWX7, A0A520H4G8, A0A520HCU1, A0A520J695, A0A520QVV1, A0A521KZ89, A0A521LW75, A0A521NCK2, A0A521QE24, A0A521QIZ0, A0A521R4Z6, A0A521TFV8, A0A521V4W6, A0A521XT95, A0A521YA10, A0A521YF32, A0A521ZAH1, A0A522C486, A0A522FW34, A0A522FZ06, A0A522H1D6, A0A522LQA7, A0A522N559, A0A522SCU1, A0A522SM68, A0A522TNK1, A0A522ZRF2, A0A523HAJ7, A0A523JU73, A0A523KVV3, A0A523LMA6, A0A527HS47, A0A527TZG1, A0A528BEE8, A0A528DCG7, A0A528H043, A0A528W5X1, A0A529B4S1, A0A529HZI4, A0A529I8I6, A0A529MD50, A0A531LH55, A0A534AAX1, A0A534C9A8, A0A534D8T9, A0A534FLN3, A0A534HHA8, A0A535EU09, A0A536UZV8, A0A536VJT1, A0A536WRS3, A0A537AZ68, A0A537MJP2, A0A537MYZ3, A0A537WPN7, A0A540W072, A0A540X6W5, A0A542BJL9, A0A542M1M4, A0A542UI05, A0A542WJ67, A0A543E747, A0A543L1V7, A0A547L2D5, A0A547NP64, A0A551Y4Q9, A0A551Y4R9, A0A552R781, A0A553GU30, A0A554RZ50, A0A554W5L0, A0A554WN62, A0A554WWB3, A0A554WXP7, A0A554X0P4, A0A554XBM0, A0A554XPW3, A0A557PEN3, A0A558HWZ8, A0A558IP30, A0A558J608, A0A559M3X1, A0A560P8L8, A0A560SZ86, A0A560WW69, A0A560X3N0, A0A560YW05, A0A561BFF5, A0A561XQT9, A0A562BQZ3, A0A562KQA0, A0A562L5R4, A0A562LCI8, A0A562PW84, A0A562QIE2, A0A562R5Q8, A0A562XKR5, A0A562ZXL0, A0A5A7MF63, A0A5A7VV85, A0A5A9F961, A0A5A9GD35, A0A5B0FSY3, A0A5B0KJN0, A0A5B0TMK2, A0A5B0X6U3, A0A5B1B971, A0A5B1BQL4, A0A5B2TG56, A0A5B7ZPU2, A0A5B8CCZ1, A0A5B8RSM2, A0A5B9D206, A0A5B9D2B7, A0A5B9GSJ7, A0A5C0DVV3, A0A5C0ZY65, A0A5C1DGX2, A0A5C1DM11, A0A5C1PZQ7, A0A5C1YQQ7, A0A5C4L5E0, A0A5C4NN28, A0A5C4PA19, A0A5C5R2D6, A0A5C5TUQ5, A0A5C5TY29, A0A5C6JPZ8, A0A5C6U3J3, A0A5C6VQI2, A0A5C7C8X3, A0A5C7G267, A0A5C7J5B3, A0A5C7L6K7, A0A5C7MI47, A0A5C7NZ45, A0A5C7QXQ3, A0A5C7RA91, A0A5C7RLE0, A0A5C7S7Z0, A0A5C7SJG3, A0A5C7UGJ5, A0A5C7UX45, A0A5C7YHN8, A0A5C7ZIM3, A0A5C8IUV9, A0A5C8MCR0, A0A5C8P3Y0, A0A5C9CP82, A0A5D0PHJ3, A0A5D3G1T4, A0A5D3G3E7, A0A5D4JJP9, A0A5D4XW62, A0A5D8YVF6, A0A5D9DCG3, A0A5E4YZW3, A0A5E6UL31, A0A5E6YE75, A0A5E7DJX2, A0A5E7HNK9, A0A5E7I9E6, A0A5E7KG71, A0A5E7N159, A0A5E7SSK7, A0A5E7T7E4, A0A5E7URB9, A0A5H2Y3B2, A0A5J4E5P2, A0A5J4ED50, A0A5J4LCS7, A0A5J5I0R4, A0A5J6FV51, A0A5J6GFE6, A0A5J6JGY6, A0A5J6WGC7, A0A5J6WHT2, A0A5K7Y0E1, A0A5L4UHB4, A0A5M8ZHW7, A0A5M9P0F9, A0A5N0E7F0, A0A5N0EBS8, A0A5N0ELU1, A0A5N0TI37, A0A5N3SDU0, A0A5N5EPX8, A0A5N5VZW8, A0A5N5W346, A0A5N5W4I5, A0A5N5X0L5, A0A5N6DES9, A0A5N6E5X9, A0A5N6EYD0, A0A5N6FU52, A0A5N6GV82, A0A5N6H0M0, A0A5N6IV93, A0A5N6TVC2, A0A5N6UUG5, A0A5N6VU39, A0A5N6X169, A0A5N6XNM2, A0A5N6YJ53, A0A5N6Z5S1, A0A5N6ZJI3, A0A5N7APE8, A0A5N7BEK9, A0A5N7C0S3, A0A5N7CXF9, A0A5N7DB68, A0A5N7W949, A0A5N7WME6, A0A5N7X627, A0A5N8T6U6, A0A5N8VKL3, A0A5N8VVK8, A0A5N9AFV9, A0A5N9I0Q3, A0A5P0YZH0, A0A5P2AVS6, A0A5P2B3R4, A0A5P2CXK9, A0A5P3MNH3, A0A5P8VXY2, A0A5P9B8K6, A0A5P9CQ04, A0A5P9J7U9, A0A5P9PBH1, A0A5Q0G954, A0A5Q0LMS2, A0A5Q0M3Q1, A0A5Q0S137, A0A5Q2VCQ2, A0A5Q2VFL6, A0A5Q3HAI2, A0A5Q3S2H2, A0A5Q4GHF4, A0A5Q5BKD4, A0A5Q5CH62, A0A5Q8CR61, A0A5R2N4K6, A0A5R8MES2, A0A5R8NF53, A0A5R8YIK7, A0A5R9JCJ4, A0A5R9PHC0, A0A5R9PW09, A0A5S3RF04, A0A5S3RQ54, A0A5S3SGP2, A0A5S3TCZ1, A0A5S3UNU2, A0A5S3UZA1, A0A5S3V1E3, A0A5S3VKT3, A0A5S3W027, A0A5S3WFI8, A0A5S3WQF5, A0A5S3WW04, A0A5S3WZC5, A0A5S3XUS7, A0A5S3XZ85, A0A5S3YNA9, A0A5S4T546, A0A5U3EN85, A0A5Y2SLJ0, A0A609CEN3, A0A627X3W7, A0A635J7C6, A0A640S205, A0A640SSW9,

Database	Protein ID
UniProt	A0A640TS69, A0A640VUZ8, A0A643FBI0, A0A645G5N6, A0A652JZF9, A0A652KE86, A0A653LNR3, A0A653VY07, A0A653Y3Y7, A0A653ZWN9, A0A654A4T5, A0A655UQ08, A0A655ZGA6, A0A656A8A5, A0A656HCW7, A0A656TEW4, A0A656THK1, A0A656UTR1, A0A656VDM0, A0A656VEZ5, A0A658B1P8, A0A658IB76, A0A659V102, A0A679J894, A0A6A1R6W3, A0A6A5QZX2, A0A6A5YGM8, A0A6A5ZPS3, A0A6A6S6W5, A0A6A7M437, A0A6A7N5R6, A0A6A8QCN0, A0A6B1KHY0, A0A6B2QZR7, A0A6B2RV70, A0A6B2SIR3, A0A6B2TLK6, A0A6B3A9J8, A0A6B3I594, A0A6B3IC41, A0A6B3MHF5, A0A6B3NFB8, A0A6B3PA98, A0A6B9QHM3, A0A6C0AZX1, A0A6C0BUY9, A0A6C1C2C4, A0A6D1T1V8, A0A6G0UEF0, A0A6G2RAV8, A0A6G2SZ84, A0A6G2TC84, A0A6G2VZ73, A0A6G3C5V4, A0A6G3RF10, A0A6G3S0Y8, A0A6G4BAT7, A0A6G4QVI9, A0A6G4R416, A0A6G4XX28, A0A6G6D033, A0A6G6SEB1, A0A6G6SQU2, A0A6G6TLV0, A0A6G7CHF7, A0A6G7PBM1, A0A6G7SS35, A0A6G7YUL4, A0A6G7ZYH1, A0A6G8C3N3, A0A6G8CM91, A0A6G8IDD1, A0A6G9FRQ1, A0A6G9SNK5, A0A6G9Y011, A0A6H0WT96, A0A6H1C9L0, A0A6H1U1X9, A0A6H2C5H9, A0A6H2H5B5, A0A6I1HJP7, A0A6I1HWI2, A0A6I1ICU8, A0A6I1MY43, A0A6I1W1C6, A0A6I2L1X5, A0A6I3SZH4, A0A6I3XJ07, A0A6I4N0U2, A0A6I4T0E7, A0A6I4YTT9, A0A6I4Z5I1, A0A6I5EAV7, A0A6I5F0F1, A0A6I5FUL3, A0A6I5HFJ9, A0A6I5YP26, A0A6I6FRT7, A0A6I6HAF1, A0A6I6HKS7, A0A6I6SIL8, A0A6I6WLW1, A0A6I6WUW6, A0A6I6Y4D3, A0A6I7D9I6, A0A6J4IC21, A0A6J4IZP6, A0A6L5BU57, A0A6L5QCJ8, A0A6L6PM36, A0A6L6PUP1, A0A6L6QFF9, A0A6L6V602, A0A6L6WV71, A0A6L8KAU8, A0A6L8L171, A0A6L8MEL4, A0A6L9FEI6, A0A6L9IHD3, A0A6L9JR39, A0A6L9ZT85, A0A6M0C1Z9, A0A6M0EN07, A0A6M0EWG5, A0A6M0FAQ8, A0A6M0G5U1, A0A6M0JA68, A0A6M0RSF4, A0A6M0SB30, A0A6M1RIA9, A0A6M1RK72, A0A6M2B667, A0A6M2BTL5, A0A6M4FH56, A0A6M4G5A5, A0A6M4GV28, A0A6M4P8B7, A0A6M5JKR9, A0A6M6IIB4, A0A6M8HS04, A0A6M8HU18, A0A6M9PQH8, A0A6M9PRT1, A0A6M9PZ77, A0A6N0BL93, A0A6N0LZR3, A0A6N1AN05, A0A6N1CHQ8, A0A6N1X386, A0A6N2JML6, A0A6N3LYU2, A0A6N3Z048, A0A6N4B1U1, A0A6N6L3V1, A0A6N6RWV1, A0A6N6VW38, A0A6N7B6Q5, A0A6N7D1J3, A0A6N7PRT9, A0A6N7Z741, A0A6N8EJ59, A0A6N8IRW0, A0A6N8S1D1, A0A6N9HRD1, A0A6N9RI47, A0A6N9VA04, A0A6P0I6K4, A0A6P0KAW3, A0A6P0ME42, A0A6P0NKQ4, A0A6P0PAL2, A0A6P0PXM4, A0A6P0RHQ4, A0A6P0TQN3, A0A6P0UZQ1, A0A6P0VB11, A0A6P0VQI8, A0A6P0WKI9, A0A6P0WWD2, A0A6P0XYM2, A0A6P0YAK5, A0A6P1GFK2, A0A6P1IJR2, A0A6P1IRZ2, A0A6P1KRU9, A0A6P1N9J2, A0A6P1NG26, A0A6P1ZP46, A0A6P2EFU7, A0A6P2ELR0, A0A6P2EU85, A0A6P2F2F8, A0A6P8AVB5, A0A6S6PM99, A0A6S6TXI9, A0A6S6U4D9, A0A6S7BJL0, A0A6V7R5U9, A0A6V7R897, A0A6V8I9B1, A0A6V8IDR8, A0A6V8R5H0, A0A735RK79, A0A7C2SCX0, A0A7C3PDR5, A0A7C4A155, A0A7C4TXL4, A0A7C4Y6V7, A0A7C5CNA9, A0A7C5GU01, A0A7C7G569, A0A7C7JE18, A0A7C7Y6C3, A0A7C8ICA5, A0A7C9G2C2, A0A7C9HTN9, A0A7C9PHS4, A0A7D3XPC4, A0A7D5DE90, A0A7D5YIS5, A0A7D6ZRQ1, A0A7D7QCJ0, A0A7D7T524, A0A7D8YN57, A0A7G3A3G9, A0A7G3GEE8, A0A7G5EIB5, A0A7G5Z7L4, A0A7G5ZHU9, A0A7G6ADU3, A0A7G6ULI2, A0A7G7Z7P7, A0A7G8K983, A0A7G8Q5V0, A0A7G8VJT7, A0A7G8VVK3, A0A7G9QXS2, A0A7G9RUB8, A0A7G9SU51, A0A7G9TGA2, A0A7H0FZP6, A0A7H0HIP2, A0A7H0I5P6, A0A7H0LM35, A0A7H1B981, A0A7H1J2X4, A0A7H1NNY3, A0A7H1NRJ9, A0A7H1RM74, A0A7H2Y461, A0A7H5FPX5, A0A7H8GDV4, A0A7H8HHH2, A0A7H8J612, A0A7H8MVV1, A0A7H8NEH1, A0A7I7M0W8, A0A7I7P8P9, A0A7I7PFS6, A0A7I7SRK7, A0A7J0D0I4, A0A7K0DP93, A0A7K1RHK2, A0A7K1W7I6, A0A7K2J6L5, A0A7K2K2A1, A0A7K2L476, A0A7K2PPY3, A0A7K2QK06, A0A7K2QT52, A0A7K2TDS9, A0A7K2Y9T3, A0A7K3D9K8, A0A7K3F2Q5, A0A7K3QZ09, A0A7L5T8V2, A0A7L5Y3G6, A0A7L6AR62, A0A7L7M792, A0A7L8SBL6, A0A7L9U901, A0A7M2GIW2, A0A7M2SMA9, A0A7M2XFE5, A0A7R6P737, A0A7R6SVZ4, A0A7S0NN38, A0A7S0PQY8, A0A7S1QKD1, A0A7S2D7C8, A0A7S2GXZ5, A0A7S2NKI2, A0A7S3F3S2, A0A7S3L7B8, A0A7S4AX46, A0A7S4H291, A0A7S4MB09, A0A7S7MY42, A0A7S7P1N3, A0A7S8E8L7, A0A7S9C6V8, A0A7S9Z0S7, A0A7T0E3H5, A0A7T0RBJ5, A0A7T1GQ15, A0A7T2S4J8, A0A7T2YTS0, A0A7T6XT23, A0A7T9XW21, A0A7U0NBA6, A0A7U2ESV4, A0A7U2MMS8, A0A7U2QYK2, A0A7U3ZF04, A0A7U4K2I5, A0A7U5Y377, A0A7U5YFG1, A0A7U6FZF3, A0A7U7J183, A0A7U9CLQ2, A0A7U9EI54, A0A7U9KTC4, A0A7V1CVW7, A0A7V2XLY5, A0A7V7KW61, A0A7V7RGT8, A0A7V7TF37, A0A7V7UED1, A0A7V7WYA9, A0A7V7ZBM6, A0A7V8FC26, A0A7V8FKJ4, A0A7V8G8X2, A0A7V8JK10, A0A7V8VZX3, A0A7V8ZVQ6, A0A7V9G4W4, A0A7V9LHZ3, A0A7W0GMG6, A0A7W0T398, A0A7W0ZTZ5, A0A7W1ALI3, A0A7W1F1Q3, A0A7W1JBD6, A0A7W1TD43, A0A7W1YGH2, A0A7W2ESZ2, A0A7W2F7N2, A0A7W2I8X8, A0A7W2ISS0, A0A7W2UTC2, A0A7W2YEH2, A0A7W2ZDL9, A0A7W3HN62, A0A7W3U1G4, A0A7W3Y619, A0A7W3ZPV3, A0A7W4A421, A0A7W4B5V8, A0A7W4CG50, A0A7W4F376, A0A7W4FJW2, A0A7W4G9J1, A0A7W4I5N2, A0A7W4ID41, A0A7W4IZD1, A0A7W4JFL6, A0A7W4JSM8, A0A7W4KBE8, A0A7W4NNC2,

Database	Protein ID
UniProt	A0A7W4NQ80, A0A7W4NUD1, A0A7W4NWW2, A0A7W4P7W6, A0A7W4PHC9, A0A7W4PJQ7, A0A7W4T8N3, A0A7W4ZQH2, A0A7W5B7H4, A0A7W5BWR9, A0A7W5D4U9, A0A7W5EMJ5, A0A7W5EQI2, A0A7W5GX09, A0A7W5J3H2, A0A7W5N663, A0A7W6AI86, A0A7W6GMI7, A0A7W6LN65, A0A7W6NVV9, A0A7W7K046, A0A7W7L8Z9, A0A7W7L905, A0A7W7L9N9, A0A7W7LGK5, A0A7W7R9N6, A0A7W8BCM5, A0A7W8D405, A0A7W8HJ33, A0A7W8K5N7, A0A7W8LIH8, A0A7W8NBY3, A0A7W8YHL0, A0A7W9PES5, A0A7W9QIM5, A0A7W9V2G8, A0A7X0AF42, A0A7X0AJV0, A0A7X0CG15, A0A7X0DGT3, A0A7X0QN92, A0A7X0TQK9, A0A7X0UDH8, A0A7X0XK45, A0A7X0XSL4, A0A7X0Y092, A0A7X0YJ81, A0A7X0Z4J4, A0A7X0ZRU0, A0A7X0ZS12, A0A7X1A3S8, A0A7X1CAK4, A0A7X1CQ85, A0A7X1EG36, A0A7X1IFG2, A0A7X1IPX9, A0A7X1KTA7, A0A7X1LXX2, A0A7X1LZN0, A0A7X1VMS0, A0A7X2I4R6, A0A7X2IKH0, A0A7X3FZU0, A0A7X3GLN4, A0A7X3KRR1, A0A7X4GSC7, A0A7X4H4B9, A0A7X4HFN8, A0A7X4RUA7, A0A7X4VWV4, A0A7X4XV36, A0A7X5ARU8, A0A7X5QSX3, A0A7X5TJ94, A0A7X5U890, A0A7X5ZW66, A0A7X6DR47, A0A7X6HRU2, A0A7X6I8N0, A0A7X7HJT3, A0A7X8TW37, A0A7X8YF72, A0A7X9TZV8, A0A7X9WXE6, A0A7X9YGG5, A0A7Y0C2A5, A0A7Y0CMD0, A0A7Y0D4M8, A0A7Y0G743, A0A7Y0M8P9, A0A7Y0P9W8, A0A7Y0W7N6, A0A7Y2KE28, A0A7Y2NZU6, A0A7Y3L1J1, A0A7Y3NE83, A0A7Y3ZZE0, A0A7Y4A2Z9, A0A7Y4A5A6, A0A7Y4A8P1, A0A7Y4CC86, A0A7Y4CM84, A0A7Y4DB90, A0A7Y4E3T2, A0A7Y4ERT8, A0A7Y4EWJ7, A0A7Y4G0K8, A0A7Y4G1Y8, A0A7Y4MQS3, A0A7Y4S3L8, A0A7Y5C6X7, A0A7Y5D6P3, A0A7Y5Z3Y9, A0A7Y6FGY9, A0A7Y6HBT1, A0A7Y6NQ06, A0A7Y6QM98, A0A7Y6WSF0, A0A7Y6XCB0, A0A7Y7HQD4, A0A7Y7I5G7, A0A7Y7ITY3, A0A7Y7IVH8, A0A7Y7IWW6, A0A7Y7J3K7, A0A7Y7TCS6, A0A7Y8GBG4, A0A7Y8GZQ3, A0A7Y8KA49, A0A7Y8WXG5, A0A7Y9HN93, A0A7Y9QTU3, A0A7Y9VV12, A0A7Z0LXJ0, A0A7Z0QP72, A0A7Z0WQY8, A0A7Z1A4I7, A0A7Z1BNY1, A0A7Z1HKJ8, A0A7Z1Q8M6, A0A7Z1S3G1, A0A7Z2KP96, A0A7Z2M3K6, A0A7Z2RNF8, A0A7Z2YD49, A0A7Z2ZSQ6, A0A7Z3C0M2, A0A7Z7HV53, A0A7Z7YH52, A0A7Z9U8B3, A0A7Z9UR00, A0A809SE00, A0A812FSM3, A0A812FZC7, A0A812G414, A0A817RM11, A0A817UUI8, A0A817VQZ8, A0A818E3W8, A0A818F3F1, A0A820VV75, A0A821BYH3, A0A821UDG3, A0A822ADC8, A0A822BUL4, A0A829R2T6, A0A829X9W0, A0A829YCE2, A0A831ESQ0, A0A831YSJ4, A0A832M4X3, A0A833AI76, A0A833ELL3, A0A833JD58, A0A836MN77, A0A836S454, A0A836V3K3, A0A837NP22, A0A837XX32, A0A838S560, A0A839HHC0, A0A839IAW6, A0A839LKA8, A0A839V512, A0A839WZ42, A0A839ZYB6, A0A840A8T8, A0A840BPX4, A0A840FHV5, A0A840HYP5, A0A840HZY0, A0A840LAZ4, A0A840P9X6, A0A840RCV7, A0A840RS91, A0A840S1X5, A0A840S8F0, A0A840Y7V7, A0A840YC80, A0A841G0A7, A0A841HMV6, A0A841QHD2, A0A841VHL9, A0A841WXI6, A0A841X6X2, A0A841XZC4, A0A841YNU4, A0A841Z2L9, A0A841Z965, A0A841ZZ31, A0A842AJE4, A0A842B3Z5, A0A842CTD2, A0A842F5C5, A0A843SDF1, A0A843SY16, A0A844AR83, A0A844D886, A0A844IF84, A0A844MHJ4, A0A844NXM8, A0A845AD58, A0A845B3Y4, A0A845GAQ0, A0A845GSS8, A0A845HGV6, A0A845HRI1, A0A845X8A3, A0A845XLQ6, A0A845Z074, A0A845ZZS7, A0A845ZZY0, A0A846AU24, A0A846B6M8, A0A846C6R6, A0A846D449, A0A846DK23, A0A846F379, A0A846G223, A0A846GUF8, A0A846HCP6, A0A846M7G3, A0A846TV60, A0A846UMR3, A0A846ZMD6, A0A847DYS9, A0A847KZ18, A0A847QX34, A0A847VK51, A0A847VLG7, A0A848EG92, A0A848F201, A0A848F8X4, A0A848HBK0, A0A848HJG2, A0A848I0H8, A0A848LTB9, A0A849MZ19, A0A849SUJ5, A0A849VCJ2, A0A850F685, A0A850NSG2, A0A850P490, A0A850P5N8, A0A850PCF8, A0A850QSI9, A0A853I5P3, A0A853ID39, A0A853IYI7, A0A853J9L4, A0A853T358, A0A854G2V1, A0A854X1W9, A0A855H1D4, A0A855ITV6, A0A855KVE6, A0A855LT05, A0A857EWF4, A0A857FMS1, A0A857GSN7, A0A857JC42, A0A858ZU05, A0A859D3B4, A0A8A1V0S7, A0A8A4KJS9, A0A8A6KJZ7, A0A8B0SP52, A0A8B3DAK0, A0A8B3N9M8, A0A8B4GHF4, A0A8B4GHV3, A0A8B4S5N3, A0A8B5WGC1, A0A8B6LPF0, A0A8B6MD23, A0A8D4B9R2, A0A8D5K214, A0A8E0QJU5, A0A8E1C4D6, A0A8E9Y9B1, A0A8G0LGQ8, A0A8G1S2T4, A0A8G1ZG63, A0A8G2CPA8, A0A8H3NKG5, A0A8H3VG56, A0A8H3YPM3, A0A8H4J4E6, A0A8H4L7X9, A0A8H4LUB2, A0A8H4X8L6, A0A8H5TEZ1, A0A8H5XUP0, A0A8H5ZZS1, A0A8H6IVL6, A0A8H6KIR2, A0A8H6MLA2, A0A8H6NI57, A0A8H6UFG9, A0A8H7A890, A0A8H7G261, A0A8H7G3D6, A0A8H7MME0, A0A8H7N467, A0A8H8RTV5, A0A8H8S663, A0A8H8TXY7, A0A8H9HXN2, A0A8H9N1L5, A0A8H9TC48, A0A8I0JJ53, A0A8I0L5F8, A0A8I0MTB6, A0A8I0MZ86, A0A8I0RN22, A0A8I1MWA9, A0A8I1NJK1, A0A8I1QRQ6, A0A8I1RCH7, A0A8I1RPZ6, A0A8I1V6A1, A0A8I2H820, A0A8J2WXB1, A0A8J2YW16, A0A8J2Z4W3, A0A8J2ZAN4, A0A8J3G092, A0A8J3JPI1, A0A8J3QHL8, A0A8J3ZXF1, A0A8J4HBR2, A0A8J5XVV4, A0A8J6MFK9, A0A8J6RIZ9, A0A8J6SAM3, A0A8J6URX1, A0A8J6VG32, A0A8J6VID9, A0A8J6VQ31, A0A8J6ZH56, A0A8J7A6Y2, A0A8J7B8K2, A0A8J7BQY3, A0A8J7CF29, A0A8J7DBX1, A0A8J7EQS5, A0A8J7G0S4, A0A8J7G7D7, A0A8J7HQ36,

**Database**	**Protein ID**
UniProt	A0A8J7HXW0, A0A8J7I6G8, A0A8J7PG39, A0A8J7SMC9, A0A8J7SQ81, A0A8J7TQM7, A0A8J7X381, A0A8J8FX68, A0A8K0M0U6, A0A8K0QK44, A0A8K0RUP8, A0A8K0VT01, A0A8S1MDB7, A0A8S1N1W9, A0A8S1P518, A0A8S1P9N7, A0A8S1QVI0, A0A8S1R9S1, A0A8S1VL66, A0A8S1VZE6, A0A8S1WMR8, A0A8S1WRJ7, A0A8S1X2H2, A0A8S1YA79, A0A8S4GLT9, A0A8S8ZXW5, A0A8T4HPQ7, A0A8T4IKP1, A0A8T5SVH2, A0A8T6IRD1, A0A8T7BP79, A0A8T7FYG4, A0A8T8WTQ9, A0A8T9MXK5, A0A914DGS7, A0A914DR31, A0A914DR68, A0A914DWR6, A0A914ESK6, A0A914FNA8, A0A914FXH1, A0A914PD96, A0A914R3I2, A0A914T618, A0A914U7D8, A0A914YHM4, A0A914YNM2, A0A914YNT5, A0A916F1L9, A0A916GQ43, A0A916REH1, A0A916SD94, A0A917NQJ7, A0A917Q8N6, A0A917RGI1, A0A918APW3, A0A918GX19, A0A918IGK1, A0A918L4A0, A0A918LMJ6, A0A918N618, A0A918PDG0, A0A918SWM4, A0A918TID7, A0A918WIM4, A0A918WYC9, A0A918YR99, A0A918ZDU7, A0A919B7G2, A0A919DG50, A0A919ED20, A0A919EEH3, A0A919GN06, A0A919GS66, A0A919SBB7, A0A919UXT3, A0A919WZE3, A0A921L4K3, A0A921NL14, A0A923C830, A0A923GLM7, A0A923JIC6, A0A923JKJ7, A0A923JSB3, A0A923MBH1, A0A923MT30, A0A923Q932, A0A923W8Q4, A0A923WM36, A0A923XHZ5, A0A923YCV9, A0A923YPD5, A0A924ABM1, A0A924E8D9, A0A924E9F2, A0A924H0I9, A0A924JXZ3, A0A924KGI7, A0A924M4G2, A0A924M5F4, A0A924MM11, A0A924MP28, A0A924PQ13, A0A924SAK7, A0A924SSF0, A0A924T9N5, A0A924TG12, A0A924TVW8, A0A924U3X1, A0A924ZE64, A0A925DKB4, A0A925JLW6, A0A925LBB1, A0A925N0Y5, A0A925N9P3, A0A925UFI5, A0A926LCS2, A0A926NBE3, A0A926PJ13, A0A926PS48, A0A926SQ13, A0A926T2V4, A0A926VAI3, A0A926VBR1, A0A926VQK1, A0A926WJM1, A0A926WY31, A0A926X851, A0A926XIT4, A0A926YAR3, A0A927FJH4, A0A928WL67, A0A929F6J9, A0A929FYB5, A0A929PSK0, A0A931CK14, A0A931J152, A0A931J1S8, A0A931JAT5, A0A931MJI6, A0A932BBS2, A0A932JUI6, A0A932SIU4, A0A932YZJ9, A0A933EZQ9, A0A933N5V4, A0A934JNV3, A0A934L9H8, A0A934Q0E1, A0A934TX54, A0A934YDS7, A0A934ZCD0, A0A934ZNK6, A0A935CGS7, A0A935DQ17, A0A935TUA9, A0A935ZBQ0, A0A936A9Z7, A0A936BXW7, A0A936CGK0, A0A936FDD6, A0A936K2V6, A0A936Z8Y4, A0A936ZSV5, A0A936ZXR8, A0A937BA71, A0A937EGP7, A0A937EIX2, A0A937ETW3, A0A938CAZ1, A0A938ZJA0, A0A938ZZU5, A0A939FLS7, A0A939H2J1, A0A939HKG0, A0A939IES4, A0A939K839, A0A939L335, A0A939SUK5, A0A940ENI5, A0A940LNH2, A0A940MXJ1, A0A940Y1F8, A0A940YEZ5, A0A941ART7, A0A941B821, A0A941BMD0, A0A941BVW0, A0A941M8E1, A0A941P3A4, A0A941Q454, A0A941QYT7, A0A941ST68, A0A941TH12, A0A941U4E1, A0A941UXX9, A0A941YQT0, A0A941YX93, A0A942F8S3, A0A942HG66, A0A942R126, A0A944DMA0, A0A944DXJ7, A0A944E4Z6, A0A944HYV0, A0A944I4Z8, A0A944J6N1, A0A944JID8, A0A944K3R8, A0A944K7S6, A0A944K9Q2, A0A944XVF1, A0A945I2K7, A0A945V6H8, A0A947BI71, A0A947DWB3, A0A947EAL1, A0A947GHM1, A0A947HXM4, A0A947PMC1, A0A947PVG0, A0A948IZL5, A0A948S254, A0A949D805, A0A949UBA7, A0A949ZRS6, A0A950B7A5, A0A950GEP2, A0A950L8E8, A0A950N7V5, A0A950PEX4, A0A950PH21, A0A950SLU6, A0A950UGC7, A0A950WP99, A0A951A5Z8, A0A951AYX0, A0A951E9Q9, A0A951ETV9, A0A951GD51, A0A951L7S9, A0A951M6S7, A0A951QQC1, A0A951UIT6, A0A952EVX5, A0A952FEB8, A0A952H017, A0A952YM38, A0A952YUB2, A0A953E8N7, A0A953H2F1, A0A953JKQ9, A0A953PIC2, A0A953T5E9, A0A954L1D1, A0A955ETR8, A0A956PIH9, A0A956TC97, A0A956Y6S7, A0A956YC42, A0A957B367, A0A957C3M7, A0A958Q7T4, A0A959HV87, A0A960TRZ2, A0A961LLH6, A0A961SIE8, A0A961Z5J2, A0A962C1P4, A0A962DC32, A0A962DNE6, A0A962E1E9, A0A962HCP7, A0A962HQ65, A0A962HXI0, A0A962I4H4, A0A962I8R5, A0A962IGD4, A0A962IIK6, A0A962N3K2, A0A963JDP7, A0A963L537, A0A963PV44, A0A963S7T9, A0A963YSI7, A0A963Z2L2, A0A963ZVF6, A0A964AQW4, A0A964CX17, A0A964EUT2, A0A964FL92, A0A964JV64, A0A965C719, A0A965KKQ7, A0A965S7E7, A0A965WH73, A0A965WJ03, A0A966IDN6, A0A966KEY5, A0A966KF32, A0A966N930, A0A966NCX8, A0A966NPT7, A0A966U2T8, A0A966WZN0, A0A966XS49, A0A966Y130, A0A967B5F7, A0A967EHY7, A0A968I253, A0A968S2Q8, A0A968UT51, A0A968VEF1, A0A968VN90, A0A969CLC3, A0A969J2Q4, A0A969JKT9, A0A969P9U1, A0A969QQB0, A0A969SHC1, A0A969U595, A0A969UQK8, A0A969VCF4, A0A970B4S8, A0A973B608, A0A973CCB8, A0A973DCG3, A0A973HF30, A0A973TPG6, A0A973XVR4, A0A973Y1Q4, A0A974Y169, A0A975CLP2, A0A975D0N6, A0A975E3P1, A0A975E9R4, A0A975FCG0, A0A975HK97, A0A975MW17, A0A975T497, A0A975UCN4, A0A975VHB9, A0A975ZHQ1, A0A976CKB9, A0A976DHG6, A0A976HKB1, A0A976HWG0, A0A976J1J0, A0A976PRU6, A0A978DV36, A0A978S5G0, A0A978SMI1, A0A978U5V1, A0A9C7FK47, A0A9C7QX54, A0A9C8U252, A0A9D0Q296, A0A9D0Y3L7, A0A9D1CAI8, A0A9D1QE37, A0A9D5EZB7, A0A9D5XIL5, A0A9D6BIL3, A0A9D6BLU1, A0A9D6T4T0, A0A9D6WVU4, A0A9D7GMT1, A0A9D7NLD6, A0A9D7RYG8, A0A9D7VJZ3, A0A9D7Z6A4, A0A9D8D3Q4, A0A9D8GW53,

Database	Protein ID
UniProt	A0A9D8LTA7, A0A9D8Q5C2, A0A9D9QHQ7, A0A9D9SCQ6, A0A9D9SPE7, A0A9D9SUQ9, A0A9D9X1I5, A0A9E0H7Q6, A0A9E0LDQ3, A0A9E0MCX9, A0A9E0SM93, A0A9E0SUA1, A0A9E0TD18, A0A9E0W241, A0A9E0WT89, A0A9E2C6D2, A0A9E2FCS5, A0A9E2FSB1, A0A9E2S1L4, A0A9E2U9H2, A0A9E2XD03, A0A9E2XTC1, A0A9E2Y3L6, A0A9E2ZPQ6, A0A9E3ALK1, A0A9E3EXI0, A0A9E3G4Y7, A0A9E3JSS5, A0A9E3JTM8, A0A9E3LT19, A0A9E4KZ32, A0A9E5BGC2, A0A9E5FS12, A0A9E5LQK8, A0A9E5PLM6, A0A9E5U568, A0A9E6DDD3, A0A9E6DGN7, A0A9E6E122, A0A9E6E339, A0A9E6JB03, A0A9E6NQA8, A0A9E6NUT9, A0A9E6NWL0, A0A9E6P256, A0A9E6RXW5, A0A9E6UZ18, A0A9E7ABP9, A0A9E8D8H7, A0A9J9FUK5, A0A9J9HDV1, A0A9J9Q788, A0A9N7CXV2, A0A9N8GW21, A0A9N9V869, A0A9N9XXA0, A0A9N9YT51, A0A9N9ZFG5, A0A9P0ELZ3, A0A9P1FNE8, A0A9P1PVU1, A0A9P2BIF8, A0A9P3B5Q7, A0A9P3HMM7, A0A9P4HJ05, A0A9P4LBG3, A0A9P4MDQ1, A0A9P4XJV5, A0A9P4YK05, A0A9P5HEK2, A0A9P5HFK0, A0A9P5U592, A0A9P5UEN3, A0A9P7ZDT9, A0A9P7ZRT2, A0A9P7ZUG0, A0A9P8KS63, A0A9P8QEQ6, A0A9P8XYT1, A0A9P8ZKB9, A0A9P8ZLS3, A0A9P9BLN3, A0A9P9DE35, A0A9P9EBP4, A0A9P9J520, A0A9P9U7X3, A0A9P9Y1Q4, A0A9P9Y2D7, A0A9Q0BGU2, A0A9Q2CYE4, A0A9Q2INX6, A0A9Q2K4V4, A0A9Q2WHV7, A0A9Q3UHL8, A0A9Q4E9U5, A0A9Q4XJ87, A0A9Q4XLP5, A0A9Q5MHX8, A0A9Q5P9P6, A0A9Q5SNC9, A0A9Q5YL33, A0A9Q6ELW1, A0A9Q6L9S0, A0A9Q8QEX4, A0A9Q8QSF5, A0A9Q8XJ14, A0A9Q8XM05, A0A9Q9IDD3, A0A9Q9MLZ8, A0A9R0P1G3, A0A9U5G6L5, A0A9W3J9W4, A0A9W3P1Z5, A0A9W3PEQ0, A0A9W3PPV0, A0A9W3S801, A0A9W3SQG0, A0A9W3UZT0, A0A9W3ZYW5, A0A9W4MJ38, A0A9W4QWV3, A0A9W4VY20, A0A9W5JJD5, A0A9W5KBN9, A0A9W5KX98, A0A9W5NVE2, A0A9W5PWE2, A0A9W5QXX1, A0A9W5RAB7, A0A9W5VGV3, A0A9W7AYK4, A0A9W7PM34, A0A9W7PQ72, A0A9W7QQC4, A0A9W7VNN1, A0A9W7VX51, A0A9W8RLJ9, A0A9W9CH54, A0A9W9E8B8, A0A9W9HF83, A0A9W9HXU8, A0A9W9MZY1, A0A9W9Q8A0, A0A9W9Q8T7, A0A9W9S6Y4, A0A9W9SA74, A0A9W9V577, A0A9X0EZ16, A0A9X0F2A2, A0A9X0HTP5, A0A9X0QUR8, A0A9X0SIT7, A0A9X0UGS4, A0A9X0UHM5, A0A9X0ULG4, A0A9X0UWG9, A0A9X0VZA7, A0A9X0XES7, A0A9X1BM01, A0A9X1GJN3, A0A9X1LA81, A0A9X1VT00, A0A9X1XRF8, A0A9X1Y4R4, A0A9X2AWI8, A0A9X2C3S0, A0A9X2C4Y4, A0A9X2E541, A0A9X2E634, A0A9X2J363, A0A9X2RK73, A0A9X2RZ74, A0A9X3B512, A0A9X3BDV8, A0A9X3CEK0, A0A9X3CLS2, A0A9X3EE58, A0A9X3FC10, A0A9X3GKX4, A0A9X3IZE8, A0A9X3RV81, A0A9X3UXZ0, A0A9X3UYX8, A0A9X3XFP0, A0A9X3YP09, A0A9X3ZTY0, A0A9X4C1V8, A0A9X4E0U7, A0A9X4F099, A0A9X4F4C0, A0A9X4L7N3, A0A9X4QYZ7, A0A9X4R7A6, A0A9X4S907, A0A9X5E8H5, A0A9X5EEK0, A0A9X5I490, A0A9X5MPF6, A0A9X5N6K3, A0A9X6B9U1, A0A9X6IFQ0, A0A9X6IHY5, A0A9X6JMA4, A0A9X6K1Q8, A0A9X6LEL3, A0A9X6LYU7, A0A9X6PHU5, A0A9X6Q236, A0A9X6QUV7, A0A9X6RBF5, A0A9X6RR35, A0A9X6SN55, A0A9X6SYM7, A0A9X6U0H2, A0A9X6UAD2, A0A9X6USG9, A0A9X6YYH2, A0A9X7AI36, A0A9X7BN70, A0A9X7C0Z4, A0A9X7CG69, A0A9X7CXT6, A0A9X7EWZ2, A0A9X7FLK9, A0A9X7G251, A0A9X7HKM4, A0A9X7JSW1, A0A9X7PJH1, A0A9X7RLU8, A0A9X7UBW4, A0A9X7V9N9, A0A9X8BXQ6, A0A9X8D0K9, A0A9X8MPU8, A0A9X8MRI2, A0A9X8NQ63, A0A9X8QCE2, A0A9X8QKF9, A0A9X8VD64, A0A9X8XML1, A0A9X8ZWQ2, A0A9X9C4Q8, A0A9X9C4X3, A0A9X9F5S7, A0A9X9G8V1, A0A9X9WQD7, A0A9X9WT10, A0A9X9XC13, A0A9Y2F360, A0AA34VG84, A0AA34XN74, A0AA35GHX6, A0AA35LQG7, A0AA35MDR0, A0AA35UNL7, A0AA35UYS3, A0AA36I106, A0AA36KJ76, A0AA36NXZ4, A0AA36PEY7, A0AA36ZKZ4, A0AA37B5V3, A0AA37KV52, A0AA37KXK7, A0AA37NRP3, A0AA37QL59, A0AA37S2H1, A0AA37SHB6, A0AA37WI26, A0AA37Z9I2, A0AA40JEY3, A0AA40SW57, A0AA41A4V5, A0AA41AJJ1, A0AA41HGP8, A0AA41XP73, A0AA41YHG4, A0AA42CNU1, A0AA42L440, A0AA42TVG5, A0AA42WRQ4, A0AA43AW95, A0AA43S514, A0AA44CKK7, A0AA44YE58, A0AA44YE68, A0AA44Z7X6, A0AA45K5A4, A0AA45MU80, A0AA45MZ04, A0AA46AFN6, A0AA46K1G1, A0AA46L5M3, A0AA46Q8H8, A0AA46SCH3, A0AA47JIE0, A0AA47LSK5, A0AA51LXT0, A0AA51MRB0, A0AA51XDY6, A0AA86WRX7, A0AA87Y418, A0AA90QGU7, A0AA90QQI8, A0AA90SV36, A0AA90XTZ5, A0AA90XUY6, A0AA91GV05, A0AA91I8C3, A0AA91KES9, A0AA91M1Y5, A0AA91U696, A0AA92LVH0, A0AA92MW37, A0AA92RA43, A0AA94EIU9, A0AA94EQ63, A0AA94LCN2, A0AA95E255, A0AA95H902, A0AA95HBU7, A0AA95SPI2, A0AA96EFI6, A0AA96JP41, A0AA96QR35, A0AA96WKZ1, A0AA96WZQ1, A0AA96X7D6, A0AA96XGY4, A0AA96XYJ1, A0AA97D1S5, A0AA97E456, A0AAC8QF69, A0AAC8RN91, A0AAC8T8Z8, A0AAC8TEG1, A0AAC8VTZ5, A0AAC8W2W4, A0AAC8Y359, A0AAC8ZN39, A0AAC9D8L3, A0AAC9FUM0, A0AAC9ITG8, A0AAC9MJM3, A0AAC9MT41, A0AAC9P9B5, A0AAC9UF36, A0AAC9WVQ3, A0AAC9X2P6, A0AAC9XD20, A0AAD0DS55, A0AAD0EPS9, A0AAD0EUJ9, A0AAD0F8D0, A0AAD0FWQ0, A0AAD0GKU6, A0AAD0J296, A0AAD0Q9A2, A0AAD0RER9, A0AAD0RLP1, A0AAD0RWI2, A0AAD0XAW4, A0AAD1CL19,

Database	Protein ID
UniProt	A0AAD1D5V4, A0AAD1ENL4, A0AAD1FAX7, A0AAD1H2M4, A0AAD1LTF8, A0AAD1MYD1, A0AAD1NE32, A0AAD2UW07, A0AAD2XU84, A0AAD3UCF3, A0AAD3WS55, A0AAD4AK02, A0AAD6C4L6, A0AAD6MW24, A0AAD7UE92, A0AAD7XQN5, A0AAD8PSY7, A0AAE0BQR4, A0AAE0F9Y6, A0AAE0JJV3, A0AAE0MDW0, A0AAE0PNS4, A0AAE0X5D6, A0AAE1ICK6, A0AAE2AP83, A0AAE2AV97, A0AAE2DM82, A0AAE2F0I9, A0AAE2ILH1, A0AAE2JLI3, A0AAE2RG98, A0AAE2S0Q5, A0AAE2U4N9, A0AAE3GGD4, A0AAE3N923, A0AAE4BXL5, A0AAE4EIR7, A0AAE4H5P1, A0AAE4M559, A0AAE4WTV3, A0AAE4WYJ7, A0AAE5ANE0, A0AAE5GMY4, A0AAE5JEN2, A0AAE5LHU8, A0AAE5SRF3, A0AAE6G632, A0AAE6J378, A0AAE6NEJ6, A0AAE6QVT1, A0AAE6VVR2, A0AAE7A967, A0AAE7DYI4, A0AAE7DYP9, A0AAE7EKY2, A0AAE7I3I1, A0AAE7K5J6, A0AAE7N731, A0AAE7YTI5, A0AAE8LGS9, A0AAE8MCS0, A0AAE8RMN4, A0AAE8S8J2, A0AAE9M0Z6, A0AAE9SLF1, A0AAF0HR14, A0AAF0VP77, A0AAF0YKW7, A0AAF1K2N2, A0AAF3J4J9, A0BVZ3, A0C0S9, A0DTE2, A0E184, A0KLH3, A0XX34, A0ZEI8, A1CCW3, A1JJ75, A1TRY0, A1UGJ0, A1VTD4, A1WFL9, A2RWQ2, A2SJH7, A3DAF2, A3P5Y4, A4CEP9, A4SLK5, A4SW04, A5FYL4, A5L075, A6F8H5, A6F8P0, A6FGN2, A6VVA9, A7MMZ3, A7MW68, A8GIM4, A8GIV7, A8KCI8, A8T1U3, A9BX03, A9FCV5, A9HAT3, A9IE29, A9IE89, A9VUH3, B0SYP1, B1WSQ6, B1XVV2, B1XZ18, B2J9R4, B4EWH0, B4WQU3, B4XYB9, B5EUS4, B5HTY5, B6ESE5, B6VN94, B7HGF2, B7IKD5, B7VMR6, B7WVQ8, B8ELX7, B8NHD6, B8R8R0, B9JT78, C1DJR0, C1EJF6, C1IC27, C1MGE9, C2XUB0, C3LLM9, C4RIV2, C5CU60, C5FUN2, C5T646, C6CD19, C6XBN8, C7BHG5, C7JGZ6, C7QK77, C9QEU4, C9Y3X4, C9YGW7, D0IFM2, D0KZG5, D0LPZ5, D0LTY3, D0SHI2, D0Z496, D2C0H9, D2YGA3, D3V0V6, D3VKH2, D3VLB7, D4B1D5, D4DCY3, D4G3R7, D4GDU4, D4I446, D4Z1R0, D5CR22, D5PCN7, D5QJ71, D5RHX8, D5VNA0, D5WYH0, D6A6P2, D6AI55, D6AU31, D6CL78, D6X6K6, D7C9E1, D7UX92, D8G0G4, D9SHM5, D9VX00, D9W5K1, D9WJ49, D9XUM7, E0SND3, E0ULH0, E1VB71, E2GIN1, E3BEU2, E3BM92, E4V1H2, E5B687, E6PKV8, E6QQT4, E6V835, E8LRH9, E8LSA8, E8M1B4, E8M6N9, F0IYM1, F0Q9H9, F0Q9V0, F1CWE1, F2K2V9, F2KBA8, F2PSU1, F2R7J2, F2RDL1, F2S319, F2SX76, F3BGN3, F3KSW6, F4GC75, F4N635, F4XKQ5, F5RAL5, F5Y1N5, F6EZV0, F7S300, F7VBE6, F7VU37, F8C7I6, F8FIA7, F8MAY6, F8S6W1, F9RF40, F9RXZ8, F9SU59, F9TE26, G0A9N9, G0RRD4, G2I6L7, G2PFA2, G3A1B5, G4CGD8, G4UE37, G6FWS5, G6XFV0, G7EEA6, G7EN59, G7UVL0, G8S7X7, G8SG27, G9N855, G9NNG9, G9Z6B9, H0A519, H0C0J0, H0IXF8, H0JNQ3, H2IWX2, H5WNV1, H5XCT1, H6NKA6, H8GLW1, I0BNI9, I0HP23, I0W5Z3, I4BRD0, I4CVY7, I4N8X6, I4VNB3, I4W148, I7ZGV1, I8TUM4, J0UD46, J2GXE0, J2KNF5, J2LEQ2, J2NVI0, J2PKP4, J2SW91, J2VJE8, J2W1P3, J2XP57, J2Y4A4, J2Y9S3, J3DSS3, J3FC61, J7FW05, J9HAB7, K0F7U8, K0Q1C9, K0SW65, K1JA31, K1WMF6, K2FHM3, K2J799, K2KDS1, K2RBV5, K8AHT7, K8XD71, K9CXS4, K9E0P1, K9G2S8, K9GUN1, K9PHN1, K9PTX0, K9PVR1, K9QCA0, K9QWK6, K9T654, K9TSB2, K9TZZ2, K9U2J8, K9UGH3, K9VR54, K9W497, K9WGQ0, K9WUM6, K9XYZ4, K9ZCH4, L0IWN2, L0ME00, L7UQ47, L8LRU3, M1QRU6, M1RU84, M2YJR8, M2ZN06, M3B3W7, M3B604, M3BK77, M4NE66, M7TDD3, M9YFW4, N0AYT7, N0D561, N1MK91, N1MKL2, N2JEF9, N6W0G3, N8S7D0, N8VLW5, N8W7M1, N8WG85, N8Y0C5, N9NXD2, N9PCE5, N9PLH5, N9PV56, N9Q883, N9QZS9, Q094T9, Q0BQA8, Q0GPY6, Q0V7I8, Q110S8, Q12FY8, Q1CXE3, Q1GXZ5, Q1K899, Q1NAE7, Q1YXB4, Q1ZUP4, Q2BZD2, Q2MFG9, Q2UF17, Q3IK57, Q3JLX1, Q3KJL6, Q3M3H9, Q48D70, Q48ER8, Q5DZF7, Q5FQD2, Q63KU4, Q6LG76, Q71BC1, Q73U85, Q79ZA2, Q7MFV2, Q7MLH0, Q7N5R2, Q7NHD5, Q7NSW1, Q81GX0, Q87Q98, Q8GGR3, Q8X0D9, Q8YSK1, Q93H45, Q9A3Z3, Q9KSS3, R0CVU2, R1C7W9, R1ELG8, R1F2H8, R1GQL7, R1HV36, R1IXJ7, R4LHF8, R7SUW9, R8NHP9, R8WXY3, R8Y302, R9B3C1, R9PMV2, S2LA86, S2LFZ3, S3C243, S3H6Q1, S3N2B2, S5SPJ1, S6GGA6, S6GTY0, S7IAF6, S9QCZ7, T0GW63, T0HDZ2, T0HK86, T0HQP4, T0KGI3, T0PM45, T0YC29, T1AX38, T1BWI1, T1XD40, T5AIG5, U1KQQ6, U1KQV7, U2ZX08, U3A4Z0, U4KAK7, U5QKM8, U5VS95, U5WWU5, U5YR81, U6SQE8, U7NNU5, U9VZZ2, V4JGM9, V4YM62, V5BKG2, V5F436, V5U651, V6K8J0, V6K9B2, V8RET9, W0V2V7, W1IM44, W1IN17, W1IZM0, W1RZU9, W1S8B6, W7BDE8, W7CFH5, W7D0Z0, W7PX59, W7PXY1, W7W415, W7W8D5, W8XZ39, X5PDV6, X7YKR0, X7ZNB6
PDB	1BK0, 1BLZ, 1DRT, 1DRY, 1DS0, 1DS1, 1E5H, 1E5I, 1E5R, 1E5S, 1GP4, 1GP5, 1GP6, 1GQW, 1GY9, 1H2K, 1H2L, 1H2M, 1H2N, 1JR7, 1NX4, 1NX8, 1OIH, 1OII, 1OIJ, 1OIK, 1VRB, 1W9Y, 1WA6, 2A1X, 2CSG, 2DBI, 2FCT, 2FCU, 2FCV, 2G19, 2G1M, 2HBT, 2HBU, 2IUW, 2JIG, 2JIJ, 2OG5, 2OG6, 2OG7, 2OPW, 2P5B, 2PXJ, 2R6S, 2RDN, 2RDQ, 2RDR, 2RDS, 2V4A, 2W2I, 2XDV, 2XML, 2XUE, 2Y33, 2Y34, 3AL5, 3AL6, 3BI3, 3BIE, 3BKZ, 3BTX, 3BTY, 3BTZ, 3BU0, 3BUC, 3DKQ, 3EAT, 3EMR, 3GJA, 3GJB, 3HQR, 3HQU, 3ITQ, 3K2O, 3K3N, 3K3O, 3KT1, 3KT4, 3KT7, 3KV4, 3KV5, 3KV6, 3KV9, 3KVA, 3KVB, 3MS5, 3N9L, 3N9M, 3N9N, 3N9O, 3N9P, 3N9Q, 3NNF, 3NNJ, 3NNL, 3NNM, 3O2G, 3ON7, 3OOX, 3OPT, 3OPW, 3OUH, 3OUI,

Database	Protein ID
PDB	3OUJ, 3PL0, 3PVJ, 3R1J, 3RZG, 3RZH, 3RZJ, 3RZK, 3RZL, 3RZM, 3S57, 3S5A, 3SWT, 3UYJ, 3W20, 3W21, 4ASK, 4BQW, 4BQX, 4BQY, 4BU2, 4BXF, 4CCJ, 4CCK, 4CCL, 4CCM, 4CCN, 4CCO, 4CSW, 4CUG, 4EYU, 4EZ4, 4EZH, 4FFA, 4IDZ, 4IE0, 4IE4, 4IE5, 4IE6, 4IE7, 4IGO, 4IGP, 4IGQ, 4IW3, 4J25, 4J5I, 4JZR, 4LIT, 4LIU, 4LIV, 4LT5, 4LXL, 4M23, 4M25, 4M26, 4M27, 4M2C, 4M2E, 4M2F, 4M2G, 4M2I, 4MG2, 4MHR, 4MHU, 4NAO, 4NE0, 4NHK, 4NHL, 4NHM, 4NHX, 4NHY, 4NID, 4NIG, 4NIH, 4NII, 4NM6, 4NPL, 4NPM, 4NRM, 4NRO, 4NRP, 4NRQ, 4O7X, 4P7W, 4P7X, 4Q5O, 4QKB, 4QKD, 4QKF, 4QKN, 4QU2, 4QWN, 4QX7, 4QX8, 4QXB, 4QXC, 4QXH, 4RGK, 4TN7, 4UF0, 4UWD, 4XAA, 4XAB, 4XAC, 4XAE, 4XBZ, 4XC9, 4XCA, 4XCB, 4Y0E, 4Y33, 4Y3O, 4Y4R, 4Y5S, 4Y5T, 4ZPI, 5A1F, 5A3P, 5A3T, 5A3U, 5A3W, 5APA, 5BK9, 5BKB, 5BKC, 5BKD, 5BKE, 5C3O, 5C3P, 5C3Q, 5C3R, 5C3S, 5C5T, 5C5U, 5DAP, 5DAQ, 5DAV, 5DAW, 5DAX, 5E6H, 5EP9, 5EPA, 5EQN, 5EQU, 5ERL, 5FPU, 5FPV, 5FUN, 5FUP, 5FV3, 5FWJ, 5FZO, 5GJ9, 5GJA, 5HSX, 5IQS, 5IQT, 5IQU, 5IQV, 5J4R, 5J92, 5JQY, 5JZ6, 5JZ8, 5JZA, 5JZU, 5KEU, 5L9B, 5L9R, 5L9V, 5LA9, 5LAS, 5LAT, 5LB6, 5LBB, 5LBC, 5LBE, 5LBF, 5LSQ, 5LUN, 5M0T, 5MOF, 5NCH, 5NCI, 5NCJ, 5NFO, 5O7Y, 5O9W, 5OA4, 5OA7, 5OA8, 5OP6, 5OP8, 5OPC, 5OX5, 5OX6, 5T22, 5TRQ, 5UQD, 5V18, 5V1B, 5V2T, 5V2U, 5V2V, 5V2X, 5V2Y, 5V2Z, 5V31, 5V32, 5V34, 5VKA, 5VKB, 5VN6, 5XEG, 5XOI, 5YBL, 5YBM, 5YBN, 5YBO, 5YBP, 5YBQ, 5YBR, 5YBS, 5YBT, 5YKN, 5YKO, 5YLB, 5ZM2, 5ZM3, 5ZM4, 6AEJ, 6AK4, 6AKZ, 6AX6, 6AX7, 6D0O, 6D1O, 6D3H, 6D3I, 6D3J, 6D3M, 6DAW, 6DAX, 6DAZ, 6DB2, 6DCH, 6EOZ, 6ETE, 6EUO, 6EUR, 6EXF, 6EXH, 6EY1, 6F0W, 6F2A, 6F2B, 6F2E, 6F6J, 6F9P, 6FXK, 6FXM, 6FXR, 6FXT, 6FXX, 6FXY, 6GEM, 6GPE, 6GPN, 6HL8, 6HL9, 6IE2, 6IE3, 6IMA, 6IMC, 6IP0, 6IP4, 6IUQ, 6JYV, 6KSF, 6KU3, 6KUN, 6KWA, 6KWB, 6L6W, 6L6X, 6L86, 6LNH, 6LSV, 6N1F, 6NIE, 6NMQ, 6NPB, 6NPC, 6NPD, 6OXH, 6OXJ, 6QGV, 6RK9, 6S0R, 6S0S, 6S0T, 6S0U, 6S0V, 6S0W, 6ST3, 6T8M, 6TE3, 6TEX, 6TEZ, 6TP5, 6TTM, 6TTN, 6TTO, 6VP4, 6VP5, 6VWQ, 6XJJ, 6YVW, 6YVX, 6YVZ, 6YW0, 6YW1, 6YW2, 6YW3, 6YW4, 6ZBN, 6ZBO, 6ZYK, 6ZYL, 7CY4, 7CY5, 7CY6, 7CY7, 7CY8, 7DE0, 7DE2, 7DT0, 7E00, 7E01, 7E05, 7E06, 7E07, 7E08, 7E37, 7E38, 7EEH, 7EKD, 7EMZ, 7ENB, 7ETK, 7ETL, 7EUS, 7EYR, 7EYS, 7EYT, 7EYU, 7EYW, 7FCB, 7JSD, 7N7V, 7OLK, 7OLL, 7OLM, 7OLO, 7OLP, 7OLQ, 7OLR, 7OLT, 7Q5V, 7Q5X, 7UMP, 7V4O, 7VBQ, 7VBR, 7VJS, 7VJV, 7VPN, 7W5S, 7W5T, 7W5V, 7WCV, 7YXG, 7YXH, 7YXI, 7YXJ, 7YXK, 7YXL, 8ACV

Table S2. Hydrophobicity values for 20 amino acids.

Amino acids	Hydrophobicity values [a]
Ile	4.5
Val	4.2
Leu	3.8
Phe	2.8
Cys	2.5
Met	1.9
Ala	1.8
Gly	-0.4
Thr	-0.7
Ser	-0.8
Trp	-0.9
Tyr	-1.3
Pro	-1.6
His	-3.2
Glu	-3.5
Gln	-3.5
Asp	-3.5
Asn	-3.5
Lys	-3.9
Arg	-4.5

[a] The hydrophobicity value of each amino acid was derived from the data of Kyte *et al*[6].

Table S3. Kinetic parameters of wild-type IDO and dominant mutants on anchoring residues.

Enzyme[a]	Substrate	K_M (μM)	k_{cat} (s^{-1})	k_{cat}/K_M ($s^{-1}\cdot mM^{-1}$)
IDO	L-Ile	10.12	0.37	36.56
MA1		9.21	0.35	38.00
IDO	L-Leu	13.05	0.28	21.46
MA2		14.93	0.37	24.78
IDO	L-Met	13.88	0.27	19.45
MA3		12.59	0.25	19.86
IDO	L-Nle	17.50	0.25	14.29
MA2		18.74	0.31	16.54
IDO	L-Nva	26.49	0.24	9.06
MA2		31.75	0.38	11.97

[a] MA1: K96L/D102Q/K105I, MA2: D101Q/D102H/K105I, MA3: K96L/D102H/K105I.

Table S4. Tunnel residues mutation library (Tunnel-Lib) design.

Mutant sites	Target residues
E66	E、M、R、Y
E97	D、T、Y
V106	Q、S、V、Y
R107	L、Y
F109	L、R
I146	I、N、Y
D173	E、Q、R

Table S5. Kinetic parameters of wild-type IDO and dominant mutants on tunnel residues.

Enzyme[a]	Substrate	K_M (μM)	k_{cat} (s^{-1})	k_{cat}/K_M ($s^{-1}\cdot mM^{-1}$)
IDO	L-Leu	13.05	0.28	21.46
	L-Nle	17.50	0.25	14.29
	L-Nva	26.49	0.24	9.06
M10	L-Nle	17.11	0.31	18.12
	L-Nva	29.48	0.35	10.26
M19	L-Leu	18.59	0.44	23.67

[a] M10: E66R/V106Y/D173E, M19: E66M/V106S/I146N/D173Q.

Table S6. Temperature distribution for the vsREMD simulation.

Replicas	Temperatures (K)
1	300
2	306.32
3	312.74
4	319.26
5	325.89
6	332.76
7	339.63
8	346.61
9	353.71
10	360.91
11	368.25
12	375.72
13	383.31
14	391.02
15	398.85
16	400

Table S7. Primers for single-point saturation mutagenesis at the Y100 and W168 sites.

Primers	Sequences (5’-3’) [a]
Y100G-F	TATAACggcGATGATGGCGGGAAAGTTAGACA
Y100G-R	CCATCATCgccGTTATATTCTTTAGATTGGAAGTAATCATTACTA
Y100A-F	TATAACgcgGATGATGGCGGGAAAGTTAGACA
Y100A-R	CCATCATCcgcGTTATATTCTTTAGATTGGAAGTAATCATTACTA
Y100V-F	ATAACgtgGATGATGGCGGGAAAGTTAGACAG
Y100V-R	GCCATCATCcacGTTATATTCTTTAGATTGGAAGTAATCATTACTA
Y100L-F	ATAACctgGATGATGGCGGGAAAGTTAGACAG
Y100L-R	GCCATCATCcagGTTATATTCTTTAGATTGGAAGTAATCATTACTA
Y100I-F	AACattGATGATGGCGGGAAAGTTAGACAGTT
Y100I-R	CCGCCATCATCaatGTTATATTCTTTAGATTGGAAGTAATCATTACTA
Y100M-F	TAACatgGATGATGGCGGGAAAGTTAGACAGT
Y100M-R	CGCCATCATCcatGTTATATTCTTTAGATTGGAAGTAATCATTACTA
Y100W-F	ATAACtggGATGATGGCGGGAAAGTTAGACAG
Y100W-R	GCCATCATCccaGTTATATTCTTTAGATTGGAAGTAATCATTACTA
Y100F-F	AACtttGATGATGGCGGGAAAGTTAGACAGTT
Y100F-R	CCGCCATCATCaaaGTTATATTCTTTAGATTGGAAGTAATCATTACTA
Y100P-F	TATAACccgGATGATGGCGGGAAAGTTAGACA
Y100P-R	CCATCATCcggGTTATATTCTTTAGATTGGAAGTAATCATTACTA
Y100S-F	ATAACagcGATGATGGCGGGAAAGTTAGACAG
Y100S-R	GCCATCATCgctGTTATATTCTTTAGATTGGAAGTAATCATTACTA
Y100T-F	ATAACaccGATGATGGCGGGAAAGTTAGACAG
Y100T-R	GCCATCATCggtGTTATATTCTTTAGATTGGAAGTAATCATTACTA
Y100C-F	ATAACtgcGATGATGGCGGGAAAGTTAGACAG
Y100C-R	GCCATCATCgcaGTTATATTCTTTAGATTGGAAGTAATCATTACTA
Y100N-F	TAACaacGATGATGGCGGGAAAGTTAGACAGT
Y100N-R	CGCCATCATCgttGTTATATTCTTTAGATTGGAAGTAATCATTACTA
Y100Q-F	ATAACcagGATGATGGCGGGAAAGTTAGACAG
Y100Q-R	GCCATCATCctgGTTATATTCTTTAGATTGGAAGTAATCATTACTA
Y100D-F	TAACcatGATGATGGCGGGAAAGTTAGACAGT
Y100D-R	CGCCATCATCatgGTTATATTCTTTAGATTGGAAGTAATCATTACTA
Y100E-F	TAACgaaGATGATGGCGGGAAAGTTAGACAGT
Y100E-R	CGCCATCATCttcGTTATATTCTTTAGATTGGAAGTAATCATTACTA
Y100K-F	AACaaaGATGATGGCGGGAAAGTTAGACAGTT
Y100K-R	CCGCCATCATCtttGTTATATTCTTTAGATTGGAAGTAATCATTACTA
Y100R-F	TATAACcgcGATGATGGCGGGAAAGTTAGACA
Y100R-R	CCATCATCgcgGTTATATTCTTTAGATTGGAAGTAATCATTACTA
Y100H-F	TAACcatGATGATGGCGGGAAAGTTAGACAGT
Y100H-R	CGCCATCATCatgGTTATATTCTTTAGATTGGAAGTAATCATTACTA
W168G-F	ACCTATTggcTTACATAAAGATGATGAACCAGTAGTATTTT
W168G-R	ATGTAAgccAATAGGTGAACTAAAAGATGGTTTTTCT

Primers	Sequences (5'-3') [a]
W168A-F	CACCTATTgcgTTACATAAAGATGATGAACCAGTAGTATTTT
W168A-R	ATGTAAcgcAATAGGTGAACTAAAAGATGGTTTTTCT
W168V-F	CACCTATTgtgTTACATAAAGATGATGAACCAGTAGTATTTT
W168V-R	ATGTAAcacAATAGGTGAACTAAAAGATGGTTTTTCT
W168L-F	CACCTATTctgTTACATAAAGATGATGAACCAGTAGTATTTT
W168L-R	ATGTAAcagAATAGGTGAACTAAAAGATGGTTTTTCT
W168I-F	CACCTATTattTTACATAAAGATGATGAACCAGTAGTATTTT
W168I-R	ATGTAAaatAATAGGTGAACTAAAAGATGGTTTTTCT
W168M-F	CACCTATTatgTTACATAAAGATGATGAACCAGTAGTATTTT
W168M-R	ATGTAAcatAATAGGTGAACTAAAAGATGGTTTTTCT
W168F-F	CACCTATTtttTTACATAAAGATGATGAACCAGTAGTATTTT
W168F-R	ATGTAAaaaAATAGGTGAACTAAAAGATGGTTTTTCT
W168P-F	CACCTATTccgTTACATAAAGATGATGAACCAGTAGTATTTT
W168P-R	ATGTAAcggAATAGGTGAACTAAAAGATGGTTTTTCT
W168S-F	CACCTATTagcTTACATAAAGATGATGAACCAGTAGTATTTT
W168S-R	ATGTAAgctAATAGGTGAACTAAAAGATGGTTTTTCT
W168T-F	CACCTATTaccTTACATAAAGATGATGAACCAGTAGTATTTT
W168T-R	ATGTAAggtAATAGGTGAACTAAAAGATGGTTTTTCT
W168C-F	CACCTATTtgcTTACATAAAGATGATGAACCAGTAGTATTTT
W168C-R	ATGTAAgcaAATAGGTGAACTAAAAGATGGTTTTTCT
W168Y-F	TCTTTTAGTTCACCTATTtatTTACATAAAGATGATGAACCAG
W168Y-R	GTTCATCATCTTTATGTAAATAAataGGTGAACTAAAAGATGG
W168N-F	CACCTATTaacTTACATAAAGATGATGAACCAGTAGTATTTT
W168N-R	ATGTAAgttAATAGGTGAACTAAAAGATGGTTTTTCT
W168Q-F	CACCTATTcagTTACATAAAGATGATGAACCAGTAGTATTTT
W168Q-R	ATGTAActgAATAGGTGAACTAAAAGATGGTTTTTCT
W168D-F	CACCTATTcatTTACATAAAGATGATGAACCAGTAGTATTTT
W168D-R	ATGTAAatgAATAGGTGAACTAAAAGATGGTTTTTCT
W168E-F	CACCTATTgaaTTACATAAAGATGATGAACCAGTAGTATTTT
W168E-R	ATGTAAttcAATAGGTGAACTAAAAGATGGTTTTTCT
W168K-F	ACCATCTTTTAGTTCACCTATTaaaTTACATAAAGATGATGAACC
W168K-R	CTGGTTCATCATCTTTATGTAAtttAATAGGTGAACTAAAAGATG
W168R-F	CACCTATTcgcTTACATAAAGATGATGAACCAGTAGTATTTT
W168R-R	ATGTAAgcgAATAGGTGAACTAAAAGATGGTTTTTCT
W168H-F	CACCTATTcatTTACATAAAGATGATGAACCAGTAGTATTTT
W168H-R	ATGTAAatgAATAGGTGAACTAAAAGATGGTTTTTCT

[a] Mutation sites are indicated by underlining in the sequence.

Table S8. Primers for iterative mutation of anchoring residues.

Primers	Sequences (5'-3') [a]
Y92-NDT-F	GTAATGATndtTTCCAATCTAAAGAATATAACTATGATGATG
Y92-NDT-R	TTGGAAahnATCATTACTATTATCTAAAATATAATCTGGTGAA
Y92-VHG-F	GTAATGATvhgTTCCAATCTAAAGAATATAACTATGATGATG
Y92-VHG-R	TTGGAAcdbATCATTACTATTATCTAAAATATAATCTGGTGAA
Y92-TGG-F	GTAATGATtggTTCCAATCTAAAGAATATAACTATGATGATG
Y92-TGG-R	TTGGAAccaATCATTACTATTATCTAAAATATAATCTGGTGAA
Q94-NDT-F	TTACTTCndtTCTAAAGAATATAACTATGATGATGGCG
Q94-NDT-R	CTTTAGAahnGAAGTAATCATTACTATTATCTAAAATATAATCTGG
Q94-VHG-F	TTACTTCvhgTCTAAAGAATATAACTATGATGATGGCG
Q94-VHG-R	CTTTAGAcdbGAAGTAATCATTACTATTATCTAAAATATAATCTGG
Q94-TGG-F	TTACTTCtggTCTAAAGAATATAACTATGATGATGGCG
Q94-TGG-R	CTTTAGAccaGAAGTAATCATTACTATTATCTAAAATATAATCTGG
S95-NDT-F	CTTCCAAndtAAAGAATATAACTATGATGATGGCGG
S95-NDT-R	ATTCTTTahnTTGGAAGTAATCATTACTATTATCTAAAATATAATC
S95-VHG-F	CTTCCAAvhgAAAGAATATAACTATGATGATGGCGG
S95-VHG-R	ATTCTTTcdbTTGGAAGTAATCATTACTATTATCTAAAATATAATC
S95-TGG-F	CTTCCAAtggAAAGAATATAACTATGATGATGGCGG
S95-TGG-R	ATTCTTTccaTTGGAAGTAATCATTACTATTATCTAAAATATAATC
K96-NDT-F	CTTCCAATCTndtGAATATAACTATGATGATGGCGGG
K96-NDT-R	ATTCahnAGATTGGAAGTAATCATTACTATTATCTAAAATAT
K96-VHG-F	CTTCCAATCTvhgGAATATAACTATGATGATGGCGGG
K96-VHG-R	ATTCcdbAGATTGGAAGTAATCATTACTATTATCTAAAATAT
K96-TGG-F	CTTCCAATCTtggGAATATAACTATGATGATGGCGGG
K96-TGG-R	ATTCccaAGATTGGAAGTAATCATTACTATTATCTAAAATAT
Y98-NDT-F	GAAndtAACTATGATGATGGCGGGAAAGTTAGACA
Y98-NDT-R	CCATCATCATAGTTahnTTCTTTAGATTGGAAGTAATCATTACTATTATC
Y98-VHG-F	GAAvhgAACTATGATGATGGCGGGAAAGTTAGACA
Y98-VHG-R	CCATCATCATAGTTcdbTTCTTTAGATTGGAAGTAATCATTACTATTATC
Y98-TGG-F	GAAtggAACTATGATGATGGCGGGAAAGTTAGACA
Y98-TGG-R	CCATCATCATAGTTccaTTCTTTAGATTGGAAGTAATCATTACTATTATC
D101-NDT-F	TAACTATndtGATGGCGGGAAAGTTAGACAGT
D101-NDT-R	CGCCATCahnATAGTTATATTCTTTAGATTGGAAGTAATCATTAC
D101-VHG-F	TAACTATvhgGATGGCGGGAAAGTTAGACAGT
D101-VHG-R	CGCCATCcdbATAGTTATATTCTTTAGATTGGAAGTAATCATTAC
D101-TGG-F	TAACTATtggGATGGCGGGAAAGTTAGACAGT
D101-TGG-R	CGCCATCccaATAGTTATATTCTTTAGATTGGAAGTAATCATTAC
D102-NDT-F	TATGATndtGGCGGGAAAGTTAGACAGTTCAA
D102-NDT-R	TTCCCGCCahnATCATAGTTATATTCTTTAGATTGGAAGTAATCA
D102-VHG-F	TATGATvhgGGCGGGAAAGTTAGACAGTTCAA
D102-VHG-R	TTCCCGCCcdbATCATAGTTATATTCTTTAGATTGGAAGTAATCA
D102-TGG-F	TATGATtggGGCGGGAAAGTTAGACAGTTCAA

**Primers**	**Sequences (5'-3')** [a]
D102-TGG-R	TTCCCGCCccaATCATAGTTATATTCTTTAGATTGGAAGTAATCA
G103-NDT-F	TGATGATndtGGGAAAGTTAGACAGTTCAATAGCAT
G103-NDT-R	CTTTCCCahnATCATCATAGTTATATTCTTTAGATTGGAAGT
G103-VHG-F	TGATGATvhgGGGAAAGTTAGACAGTTCAATAGCAT
G103-VHG-R	CTTTCCCcdbATCATCATAGTTATATTCTTTAGATTGGAAGT
G103-TGG-F	TGATGATtggGGGAAAGTTAGACAGTTCAATAGCAT
G103-TGG-R	CTTTCCCccaATCATCATAGTTATATTCTTTAGATTGGAAGT
G104-NDT-F	GATGGCndtAAAGTTAGACAGTTCAATAGCATAAATGAT
G104-NDT-R	CTAACTTTahnGCCATCATCATAGTTATATTCTTTAGATTG
G104-VHG-F	GATGGCvhgAAAGTTAGACAGTTCAATAGCATAAATGAT
G104-VHG-R	CTAACTTTcdbGCCATCATCATAGTTATATTCTTTAGATTG
G104-TGG-F	GATGGCtggAAAGTTAGACAGTTCAATAGCATAAATGAT
G104-TGG-R	CTAACTTTccaGCCATCATCATAGTTATATTCTTTAGATTG
K105-NDT-F	GGGndtTTAGACAGTTCAATAGCATAAATGATAGC
K105-NDT-R	AACTGTCTAACahnCCCGCCATCATCATAGTTATATTC
K105-VHG-F	GGGvhgTTAGACAGTTCAATAGCATAAATGATAGC
K105-VHG-R	AACTGTCTAACcdbCCCGCCATCATCATAGTTATATTC
K105-TGG-F	GGGtggGTTAGACAGTTCAATAGCATAAATGATAGC
K105-TGG-R	AACTGTCTAACccaCCCGCCATCATCATAGTTATATTC

[a] Mutation sites are indicated by underlining in the sequence.

Supplementary Movie

Movie S1. Conformational change of loop2 from open to closed.
Loop2 is colored as steel blue, β4 as dark salmon, and the ferrous ion as light pink. The MD trajectory clearly reveals loop2 undergoes a remarkable conformational change, whereas a little conformational fluctuation is observed in other regions of IDO.

Supplementary References

[1] Fraczkiewicz, R. and W. Braun, *J. Comput. Chem.* **1998**. *19*, 319-333, https://doi.org/10.1002/(SICI)1096-987X(199802)19:3%3C319::AID-JCC6%3E3.0.CO;2-W.

[2] Magala, P., R.E. Klevit, W.E. Thomas, E.V. Sokurenko, and R.E. Stenkamp, *Proteins: Struct. Funct. Bioinf.* **2020**. *88*, 593-603, https://doi.org/https://doi.org/10.1002/prot.25840.

[3] Lobanov, M.Y., N.S. Bogatyreva, and O.V. Galzitskaya, *Mol. Biol.* **2008**. *42*, 623-628, https://doi.org/10.1134/S0026893308040195.

[4] Zheng, C.N., W.Q. Wei, J. Wen, W. Song, J. Wu, R. Wang, D.J. Yin, X.L. Chen, C. Gao, J. Liu, and L.M. Liu, *Angew. Chem. Int. Edit.* **2024**. *63*, e202406060, https://doi.org/https://doi.org/10.1002/anie.202406060.

[5] Guan, J.J., Y.L. Lu, Z.X. Dai, S.Y. Zhao, Y. Xu, and Y. Nie, *Molecules* **2023**. *28*, 1854, https://doi.org/10.3390/molecules28041854.

[6] Kyte, J. and R.F. Doolittle, *J. Mol. Biol.* **1982**. *157*, 105-132, https://doi.org/https://doi.org/10.1016/0022-2836(82)90515-0.
